# Data-Driven Search Algorithm for Discovery of Synthesizable Zeolitic Imidazolate Frameworks

**DOI:** 10.1021/jacsau.5c00077

**Published:** 2025-03-07

**Authors:** Soochan Lee, Hyein Jeong, Sungyeop Jung, Yeongjin Kim, Eunchan Cho, Joohan Nam, D. ChangMo Yang, Dong Yun Shin, Jung-Hoon Lee, Hyunchul Oh, Wonyoung Choe

**Affiliations:** † Department of Chemistry, Ulsan National Institute of Science and Technology, Ulsan 44919, Republic of Korea; ‡ Computational Science Research Center, Korea Institute of Science and Technology (KIST), Seoul 02792, Republic of Korea; § KU-KIST Graduate School of Converging Science and Technology, Korea University, Seoul 02841, Republic of Korea; ∥ Graduate School of Carbon Neutrality, Ulsan National Institute of Science and Technology, Ulsan 44919, Republic of Korea; ⊥ Graduate School of Artificial Intelligence, Ulsan National Institute of Science and Technology, Ulsan 44919, Republic of Korea; # Department of Mechanical Engineering, Ulsan National Institute of Science and Technology, Ulsan 44919, Republic of Korea

**Keywords:** metal–organic frameworks, zeolitic imidazolate frameworks, zeolite analogues, adsorption, zeolite conundrum, chemical intuition

## Abstract

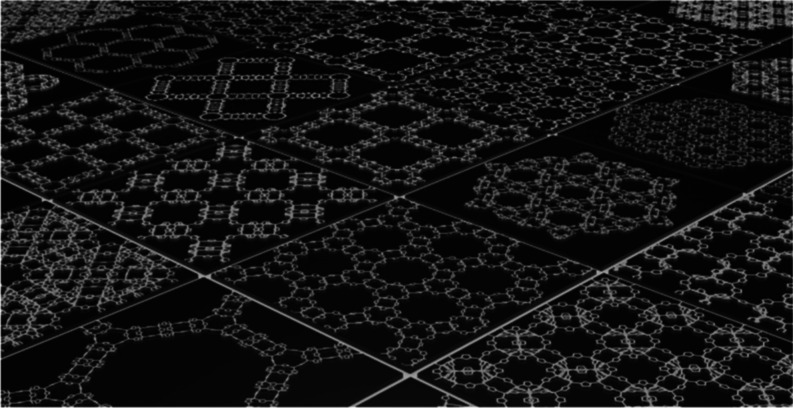

Zeolitic imidazolate frameworks (ZIFs), metal–organic analogues of zeolites, hold great potential for carbon-neutral applications due to their exceptional stability and porosity. However, ZIF discovery has been hindered by the limited topologies resulting from a mismatch between numerous predicted and few synthesized zeolitic networks. To address this, we propose a data-driven search algorithm using structural descriptors of known materials as a screening tool. From over 4 million zeolite structures, we identified potential ZIF candidates based on O–T–O angle differences, vertex symbols, and T–O–T angles. Energy calculations facilitated the ranking of ZIFs by their synthesizability, leading to the successful synthesis of three ZIFs with two novel topologies: UZIF-31 (*uft*1) and UZIF-32, -33 (*uft*2). Notably, UZIF-33 exhibited remarkable CO_2_ selective adsorption. This study highlights the synergistic potential of combining structural predictions with chemical intuition to advance material discovery.

## Introduction

Zeolitic materials, such as in zeolites^1−3^ and zeolite imidazolate frameworks (ZIFs)^4−6^—organic–inorganic hybrid cousins, are all assembled from tetrahedral structural building units and extremely useful in numerous areas,^3^ ranging from biomedical^7−9^ to petrochemical,^10−13^ and further to carbon neutrality applications.^14−16^ Despite their significant presence in both industry and academia for the past several decades, surprisingly, only 255 zeolites^17^ and about 50 ZIFs^6^ are known to exist to date. On the contrary, the possible zeolitic materials are well over few million accumulated in hypothetical zeolite databases.^18,19^ This striking mismatch between experiment and prediction outcomes has become a “zeolite conundrum”,^20^ demanding significant strategic breakthroughs in material discovery.

Advanced computational methodologies are such efforts to accelerate material discovery where digital tools such as large language models,^21−23^ materials informatics,^24−26^ and artificial intelligence,^27−29^ are commonly utilized. However, the vast number of hypothetical material candidates produced by these digital tools often face a significant hurdle–experimental verification.^30,31^ To overcome this, an integrated approach is essential where computational predictions are closely aligned with targeted experimental efforts.^32−35^ In this regard, incorporating the chemical intuition of expert chemists into structure prediction could accelerate the materials discovery^32−40^ by enhancing the screening process^41^ and reducing computational costs,^42^ similar to the advances in drug discovery.^43,44^

In this context, we have focused such an approach on ZIFs, to test its potential in materials discovery. ZIFs mimic the tetrahedral units (TO_4_, T = Si or Al) in zeolites through coordination bonds formed between four-coordinated metal cations and imidazolates (Im, hereafter).^4^ These materials hold the potential to supplement the zeolite ecosystems due to exceptional chemical stability and high porosity.^4,15^ Despite strong attention to utilizing ZIFs, there are only 38 known ZIF topologies,^6,45^ significantly fewer than 255 realized zeolites.^17^ Even with extensive efforts in high-throughput synthesis, the chances of discovering new ZIF crystals remain extremely low, as reported by Yaghi et al.^15^ Compared to zeolites, where synthesizability evaluation has advanced significantly,^46−49^ the targeted synthesis of ZIFs from predicted structures remains rare.^50,51^ ZIFs require an elaborate structural analysis and synthetic trials to evaluate their feasibility.

Here, we present a new data-driven search algorithm for potential ZIF candidates extracted from over 4 million zeolite structures. These feasible candidates were derived from intuitive structural descriptors such as O–T–O angle difference, vertex symbol, and T–O–T angle, all of which play a crucial role in the construction of zeolitic networks.^52−54^ Through energy calculations, we effectively ranked the synthesizability of affordable ZIFs for targeted synthesis. To our surprise, this approach led to the discovery of three previously unknown ZIFs, UZIF-31 (*uft*1), UZIF-32 (*uft*2), and UZIF-33 (*uft*2), featuring unprecedented topologies matched with our highly synthesizable data set. Structural analysis unveils the internal hydrogen bonding network for framework stabilization. Notably, UZIF-33 stands out for exceptional adsorption selectivity in CO_2_ over CH_4_, positioning it as a model material for methane purification,^55^ contributing to carbon neutrality.^56^

## Results and Discussion

### Target System from the Zeolite Database

Given the structural interchangeability between zeolites and ZIFs, the data-driven discovery for feasible ZIFs was conducted by considering both the International Zeolite Association (IZA)^17^ and the hypothetical zeolite database.^18^ In total, these databases have a sum of 4,450,797 zeolites (255 in IZA and 4,450,542 in the hypothetical database). The multitude of zeolitic networks, encompassing hypothetical zeolite databases, contain an extensive amount of structural data that complicates the pursuit of future targets for ZIF discovery (Table S1). In comparison to the successful reticular design of MOFs,^57,58^ limiting the topological system could prove efficient for pinpointing a subset of zeolite-like materials. Initially, our focus was directed toward analyzing the number of nodes (the number of symmetrically identical nodes; *n*-nodal) present in synthesized ZIF topologies (Figures S1 and S2). Notably, a major portion of ZIFs was either uninodal or binodal nets (79% or 30 out of 38). Of particular interest were the trinodal topologies in ZIFs, which were notably scarce among the 38 ZIF topologies (merely 5%, 2 out of 38), even in tetranodal ZIFs accounting for 10% (4 out of 38). We hypothesized that these trinodal ZIFs might serve as missing links and hold a high potential for introducing new ZIFs. Consequently, these trinodal topologies were identified as screening targets.

### Screening Process Using Structural Descriptors

Employing efficient descriptors to screen through vast entries in databases could prove to be an effective method for identifying synthesizable materials. In earlier work, our group developed structural descriptors for feasible ZIFs, such as node number, vertex symbol, and T–O–T angle, which are associated with the topological frameworks within zeolitic networks.^45^ In this study, we will utilize intuition-based structural descriptors, specifically the O–T–O angle difference, vertex symbol, and T–O–T angle, derived from common geometrical features in ZIFs.

**Figure 1:**
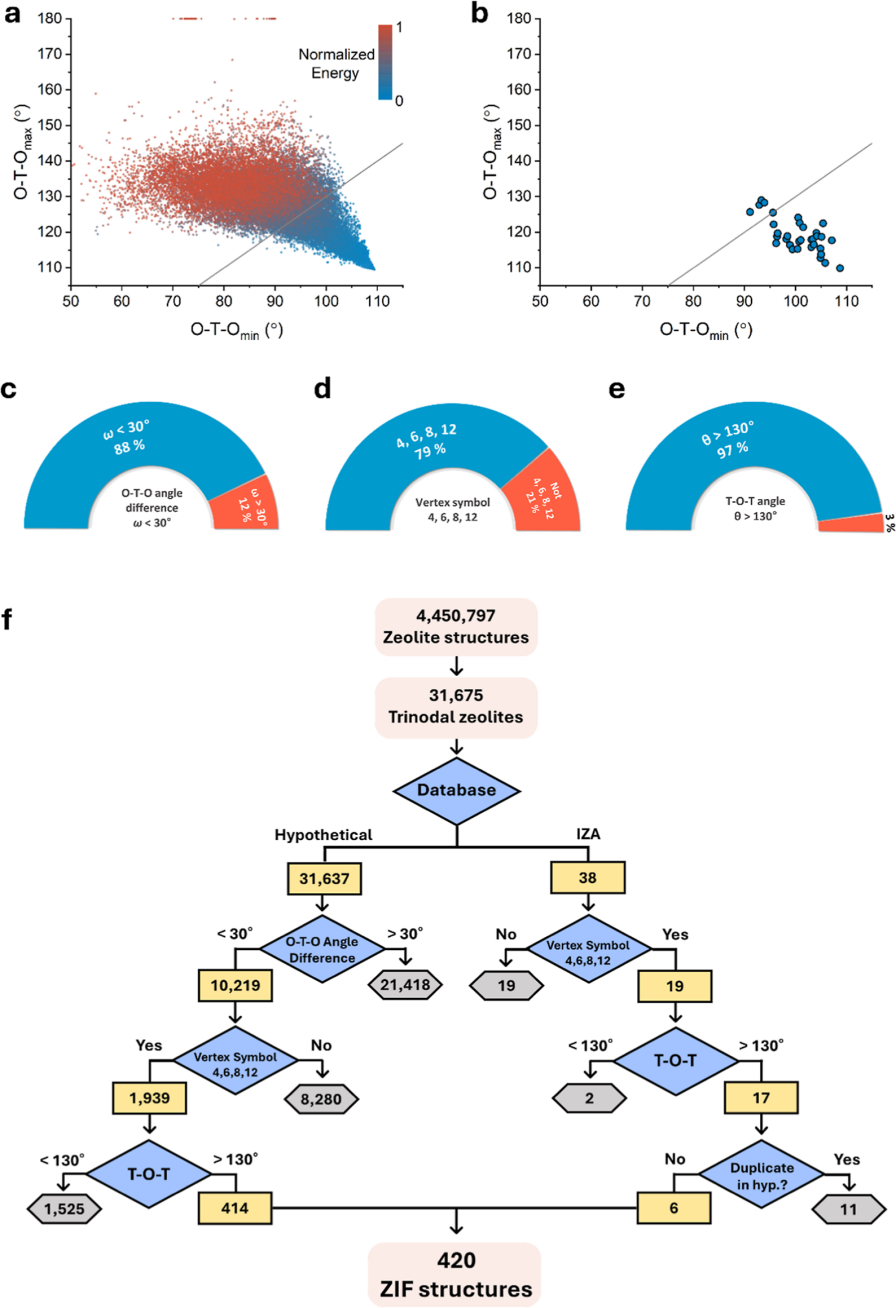
O–T–O angle difference (ω) in (a) 31,637 trinodal hypothetical zeolites and (b) 33 known ZIFs. The gray line indicated ω = 30°; under the line (ω < 30°) and above the line (ω > 30°). The percentage of known ZIFs with satisfying three structural descriptors. (c) First, O–T–O angle difference; ω < 30°. (d) Second, the vertex symbol; 4, 6, 8, and 12. (e) Third, T–O–T angle; >130°. (f) Flowchart for finding topology candidates for synthesizable ZIFs from the zeolite database. After applying the descriptors, the number of zeolites reduced from an initial count of 4,450,797 to 420 ZIF topologies.

In zeolites, the O–T–O angles are kept at the ideal angle for a 4-coordinated tetrahedral node (approximately 109.5°) to provide stability to the frameworks.^54^ Nevertheless, the hypothetical zeolite database contains numerous impractical zeolite structures marked by highly deviated tetrahedral geometries from the ideal O–T–O angle, particularly encompassing energetically unstable zeolites (Figure 1a). Avoiding the severely distorted geometry within the parent zeolite structures proves to be an effective approach when dealing with an extensive data set. To manage this, we defined a cutoff parameter, ω (the angle difference between the maximum and minimum O–T–O angles), with a threshold of <30° for ω in ZIFs. This selection was based on the observation that nearly all ZIFs (88%; 29 out of 33, the exclusion for those featuring partially 3-c nets; **moc** and **moz**, non-Zn ZIFs; **mog**, **ict**, and **dia-c**) exhibited ω values below 30° (Figures 1b, S3 and S4). The first descriptor is the limitation to the O–T–O angle difference (ω < 30°) in zeolitic topologies (Figure 1c).

The second and third descriptors, namely, the vertex symbol (4, 6, 8, or 12 combinations) and the T–O–T angle (θ > 130°), were adopted from our recent publication due to their proven efficacy in screening for reliable ZIF structures.^45^ Vertex symbols represent the connectivity and symmetry of nodes (or vertices) in each structure, which are essential for understanding the topological characteristics of the framework. In zeolites, vertex symbols indicate the size and number of the shortest rings at the angles of each 4-c vertex within the zeolite networks. Specifically, 79% (30 of 38) of ZIFs shared vertex symbols involving combinations of these ring sizes, 4, 6, 8, or 12 combinations (Figure 1d and Table S2). The distribution of T–O–T angles was notably narrow, with 97% (37 of 38) of ZIF topologies displaying angles that did not exceed 130° (Figures 1e and S5). Thus, our second and final descriptors are defined by the limitation of vertex symbols to 4, 6, 8, or 12 combinations and the T–O–T angle exceeding 130°, respectively.

Following the approach guided by the structural descriptors, we initiated data filtering in the following sequence: starting with O–T–O angle difference (ω < 30°), followed by vertex symbols (4, 6, 8, and 12), and then T–O–T angles (θ > 130°). The number of initial zeolites with trinodal nets drastically reduced to 31,675 from the total 4,450,797 zeolites. The first descriptor, the O–T–O angle difference (ω < 30°), was applied to 31,637 hypothetical zeolites and resulted in only 10,219 structures. Subsequently, after the applying second (vertex symbol; 4, 6, 8, and 12) and third (T–O–T angle; θ > 130°) descriptors, a total of 420 topologies, including hypothetical and IZA zeolites, remained in the pool for future ZIFs (f-ZIF), marking a remarkable reduction from over 4 million zeolite structures (Figure 1f). Out of 420 ZIF topologies, 23 topologies are found in IZA (BPH, AFS, ATS, SFW, AEI, AFT, SAV, PWN, OSI, MSO, UEI, AWO, AFV, OWE, AEL, THO, and AFG) and the reticular chemistry structure resource^58^ database (**reo-t**, **ucb**, **tdc**, **gcd**, **cfd**, and **cmc**), including a realized ZIF topology^50^ (**ucb**; ZIF-412, -413, and -414) (Table S3).

### Synthesizable Future ZIFs

Based on the 420 topologies that resulted from our structural descriptors, we proceeded to carry out in silico construction of future ZIFs (f-ZIFs) using a topologically based crystal constructor (ToBaCCo)^59^ (Figure S7). Once the initial structures were generated, we performed geometry optimization using the force field for ZIFs^60^ (ab initio-derived MOF–FF for ZIFs; hereafter FF) through the LAMMPS.^61^ For the assessment of cell volume changes in known ZIFs, we applied both FF and density functional theory (DFT) calculations, as demonstrated by testing against experimental cell volumes (Figures S8 and S9). The resulting volume change in known ZIFs with FF was found to be a mere −1.41% compared to the experimental cell volume, displaying minimal divergence from the DFT outcome, which offers greater computational precision but also consumes more time and registers at −0.93%. Several results have deliberated the synthesizability of porous crystalline materials^40,62,63^ using low-level calculations to calculate the framework energy instead of the more computationally intensive periodic DFT method.

**Figure 2:**
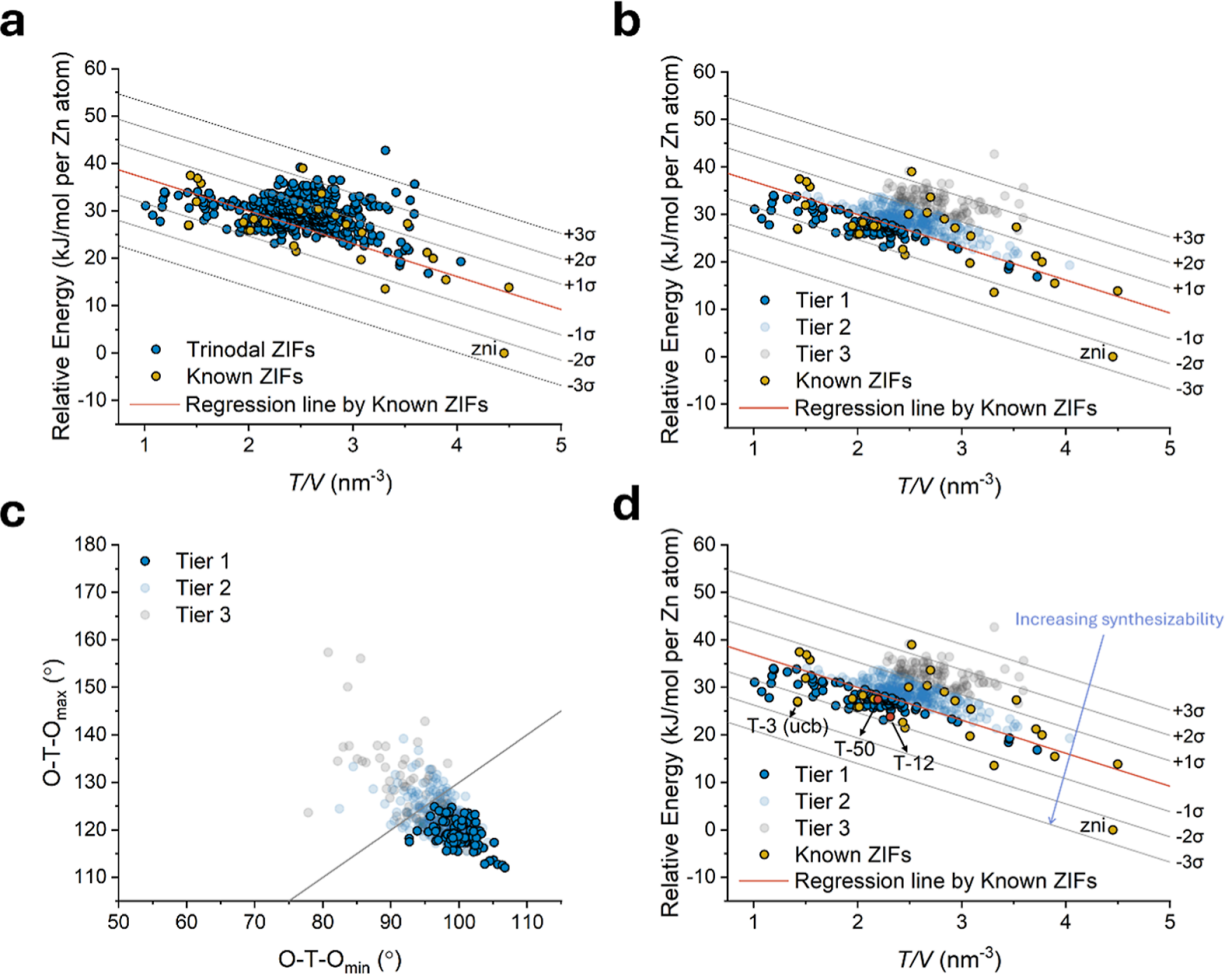
(a) The calculated total energy of trinodal ZIFs with density. The red line represents the regression line set by known ZIF topologies for prediction intervals. (b) Synthesizability tier of 420 f-ZIFs. Tier 1 (90/420; T-1 to T-90), Tier 2 (233/420; T-91 to T-323), and Tier 3 (97/420; T-324 to T-420) were divided in the section between the prediction intervals. (c) The O–T–O angle difference (ω) in 420 f-ZIFs with synthesizable Tiers. 90 Tier 1 ZIFs are involved in ω < 30°. (d) Synthesizability ranking was set by the distance from the −3σ line. Realized topologies in this study, T-12, and T-50, are denoted as red dots. T-3 is identical to the realized **ucb** topology.

**Figure 3:**
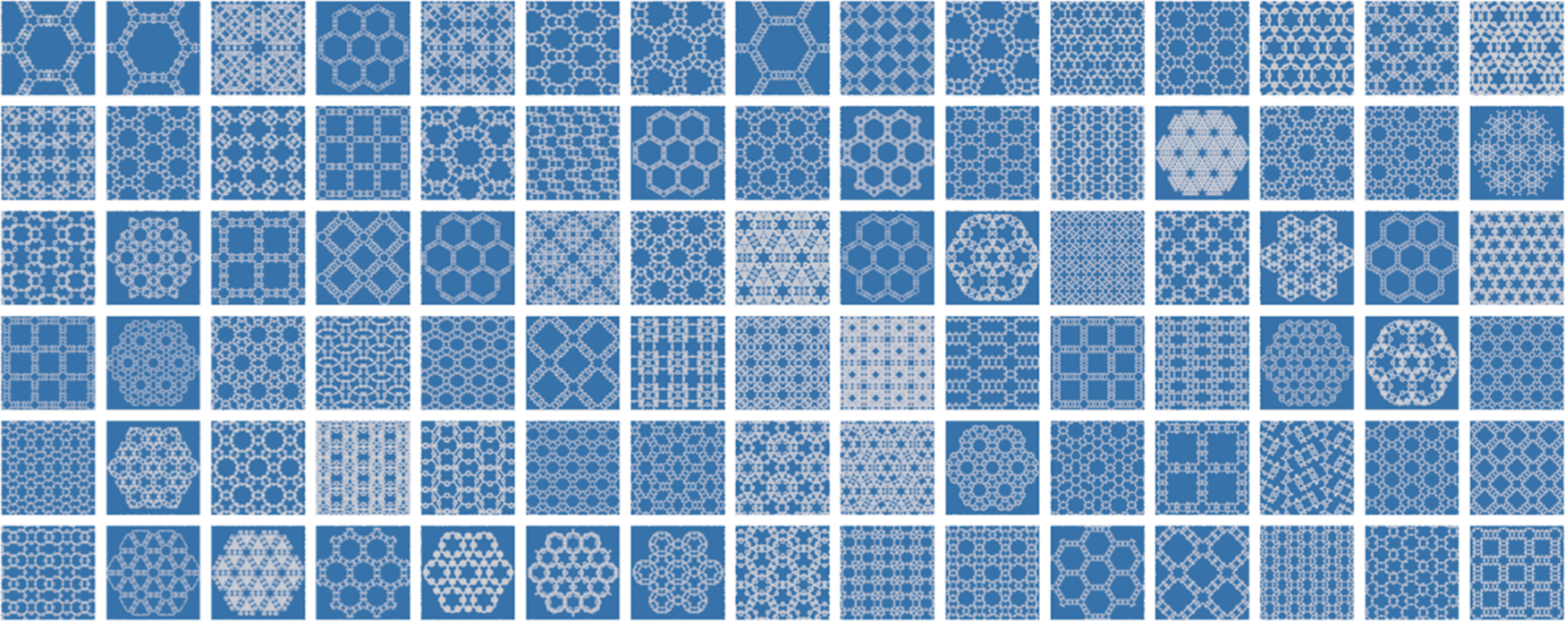
Schematic illustrations with tiling projections of the generated 90 f-ZIFs (from T-1 to T-90, left to right) belong to Tier 1 synthesizability (see Figure S14 and Table S4 for topology label and structural information on each tile, respectively).

**Figure 4:**
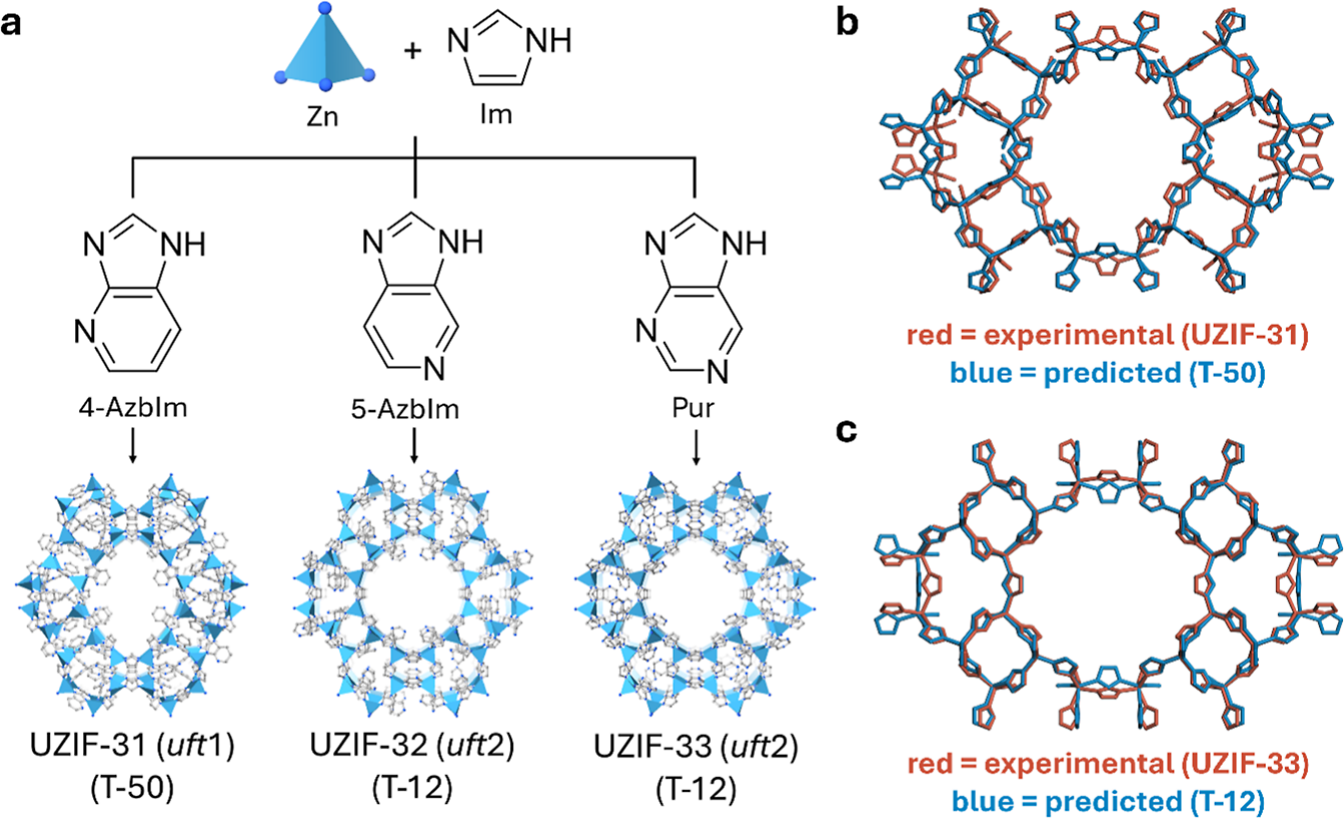
(a) Synthetic scheme of UZIF-31 (*uft*1), UZIF-32 (*uft*2), and UZIF-33 (*uft*2) involving a solvothermal reaction with Zn ion, Im, and AzbIm derivatives. The topologies for *uft*1 and *uft*2 are identical to T-50 and T-12 in Tier 1, respectively. Overlay of crystal structures between (b) synthesized UZIF-31 (red) versus predicted T-50 (blue) and (c) UZIF-33 (red) versus predicted T-12 (blue). Functional groups and hydrogens are omitted for clarity.

We also conducted energy calculations on 30 known ZIFs [converted to Zn(Im)_2_ formula] after optimization using the FF and then plotted the energy against the framework density (nm^–3^), *T*/*V* (Figure S10). Interestingly, ZIF topologies exhibited a similar trend to several DFT results,^45,64,65^ where the energy increased as the density decreased, with the most stable **zni** topology. We proceeded with the assumption that synthesizable ZIFs would likely align closely with real ZIFs and thus calculated a prediction distribution around the linear regression of known ZIF topologies. Intriguingly, the prediction interval within ±2σ encompassed 93% of known ZIFs (two exceptions; **ana** and **zni** within ±3σ). This analysis was further extended to the newly generated 420 f-ZIFs, which were plotted alongside the known ZIF set (Figure 2a). In crystal structure prediction, porous frameworks that possess lower energy at a given density are more likely to be achievable through synthesis.^35,66−70^ Based on this approach, we established a categorization: Tier 1 f-ZIFs with high synthesizability were located under the regression line (21%, 90 out of 420), Tier 2 f-ZIFs with moderate synthesizability were positioned between the regression line to the +1σ range (56%, 233 out of 420), and Tier 3 f-ZIFs with low synthesizability fell beyond the +1σ range (23%, 97 out of 420) (Figures 2b and S11). Remarkably, all Tier 1 f-ZIFs successfully passed the validation criteria based on the first descriptor, which involves assessing the O–T–O angle difference (ω < 30°), in contrast to several Tier 2 and 3 f-ZIFs that exceeded the established threshold for reliable O–T–O angle differences (Figures 2c and S13). This observation emphasizes the high efficiency of categorizing synthesizability by energy level in revealing chemically plausible structures. In essence, the ranking of synthesizability was established by proximity to the −3σ line (Figure 2d). Consequently, all 420 f-ZIFs were assigned names based on their synthesizability ranking, denoted as T-*n* (with T-1 being the most feasible ZIF and T-420 representing the least feasible). An overview of all 90 Tier 1 f-ZIFs (T-1 to T-90) is illustrated in Figure 3. Significantly, we were able to experimentally validate our rational strategy through the successful synthesis of two ZIF topologies, T-12 and T-50, both of which are in Tier 1 (Figures 2d and 4a).

### Synthetic Strategy

In ZIF chemistry, the link–link interactions,^50^ mainly van der Waals forces, between Im linkers stand as a crucial factor in stabilizing distinct cages and topologies, and introducing hydrogen bonds within ZIF crystals presents a challenging work.^71^ Our objective was to introduce weak hydrogen bonds^72^ into ZIF construction using derivatives of azabenzimidazole (AzbIm). A unique combination of Im and three AzbIm derivatives, namely, 4-azabenzimidazole (4-AzbIm), 5-azabenzimidazole (5-AzbIm), and purine (Pur), successfully facilitated the synthesis of new ZIF topologies (Figure 4a). Following more than 1600 synthesis attempts, UZIF-31 [Zn(Im)_0.88_(4-AzbIm)_1.12_], UZIF-32 [Zn(Im)_1.25_(5-AzbIm)_0.75_], and UZIF-33 [Zn(Im)_1.25_(Pur)_0.75_] were synthesized through solvothermal synthesis using a mixture of Zn(NO_3_)_2_·6H_2_O, Im, and the corresponding AzbIm derivative (4-AzbIm, 5-AzbIm, or Pur) in *N*,*N*-dimethylformamide (DMF) at 120 °C for 24–96 h with no additives (see the detail in experimental procedures, Supporting Information). In nearly 1600 experimental attempts, we primarily focused on varying the types and combinations of imidazole linkers while maintaining consistent conditions for the solvent (DMF), temperature (120 °C), and metal precursor (Zn(NO_3_)_2_·6H_2_O). Other variables included the metal-to-linker ratio (1:2–1:6) and reaction times (24–144 h). The experimental outcomes exhibited significant diversity, including cases with no solid formation, noncrystalline products, known ZIF structures, and undefined materials. Among the undefined materials, single-crystal X-ray diffraction (SCXRD) data were successfully obtained from three trials, which aligned well with our computational predictions. The bulk purity of all crystals was verified by comparing their powder X-ray diffraction patterns to simulated ones (Figures S22–S24).

### Structural Analysis

**Figure 5:**
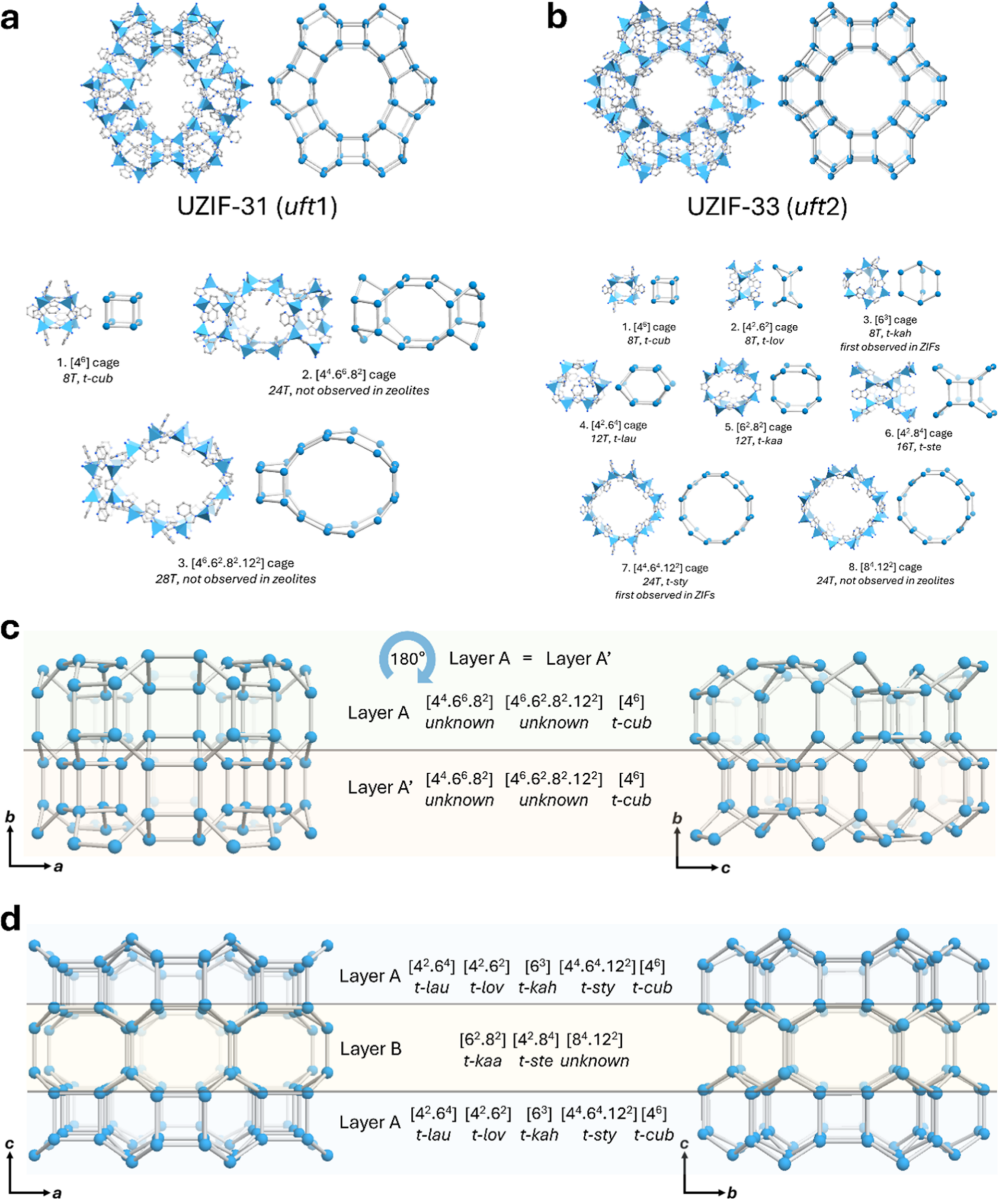
(a) Structural illustration of UZIF-31 (*uft*1) along the [010] direction and their constructed three types of cages, [4^6^] cage; *t-cub*, [4^4^.6^6^.8^2^] cage, and [4^6^.6^2^.8^2^.12^2^] cage. (b) Structural illustration of UZIF-33 (*uft*2) along the [001] direction and their constructing eight types of cages, [4^6^] cage; *t-cub*, [4^2^.6^2^] cage; *t-lov*, [6^3^] cage; *t-kah*, [4^2^.6^4^] cage; *t-lau*, [6^2^.8^2^] cage; *t-kaa*, [4^2^.8^4^] cage; *t-ste*, [4^4^.6^4^.12^2^] cage; *t-sty*, and [8^4^.12^2^] cage. Blue polyhedron and ball, Zn; gray, C; blue, N. (c) Schematic representation of UZIF-31 along the [001] and [100] directions as the assembly to layer stacking pattern, A–A′. (d) Schematic representation of UZIF-32 and -33 along the [010] and [100] directions as the assembly to the layer stacking pattern, A–B–A.

UZIF-31 (*uft*1) with Im and 4-AzbIm crystallized in the orthorhombic *Imma* (no. 74) space group (Table S6). The whole crystal structure of UZIF-31 was determined by SCXRD with help from the confirmed composition by using ^1^H NMR spectroscopy (Figure S28). The topology of UZIF-31 exhibited an unprecedented 4,4,4-c topology with the point symbol of (4^2^.6^2^.8^2^)(4^3^.6.8^2^)(4^3^.6^2^.8) named *uft*1.^73^ This novel topology, *uft*1, was documented in the personal topology repository^74^ and not observed in any materials even zeolites. Notably, the topology of *uft*1 exhibits the same topological network as PCOD 8095196, also known as 4,4,4T-3872HZ, which is among hypothetical zeolites proposed by Deem et al.^19,75^ Interestingly, the topology of UZIF-31 is exactly matched with one of Tier 1, T-50 (Figures 4b and S16). Despite a slight distortion of Im, the difference in cell volume between UZIF-31 and the predicted structure was merely +6.14%. During the topological analysis of *uft*1, three types of tiles compose UZIF-31 (Figure 5a). One of the tiles is a [4^6^] cage, which is known as the *t*-*cub*, and the others ([4^4^.6^6^.8^2^] and [4^6^.6^2^.8^2^.12^2^] cages) are not observed in any zeolites. The largest pore opening with a 12-membered ring (MR) was observed within the surrounding [4^6^.6^2^.8^2^.12^2^] cage.

UZIF-32 and -33 (*uft*2) crystallized within the orthorhombic *Cmmm* (no. 65) space group. The composition of the crystals was confirmed by SCXRD and ^1^H NMR (Tables S7 and S8 and Figures S29 and S30). Thus, far, due to the identical net, the topological analysis of UZIF-32 has been considered the same as that of UZIF-33 for clarity. UZIF-33 also presented a novel 4,4,4-c topology with the point symbol of (4.6^4^.8)(4^2^.6^3^.8)(4^3^.6^2^.8) named *uft*2,^73^ not encountered in any other porous materials, including zeolite structures. Similar to *uft*1, the topology of *uft*2 shows the identical topological network with PCOD 8096930, also referred to as 4,4,4T-3884HZ, in hypothetical zeolites suggested by Deem et al.^19,75^ The topology of UZIF-33 is completely matched to one of Tier 1, T-12, with a slight cell volume difference of +4.68% (Figures 4c and S19). Eight types of tiles ([4^6^] cage as *t-cub*, [4^2^.6^2^] cage as *t-lov*, [6^3^] cage as *t-kah*, [4^2^.6^4^] cage as *t-lau*, [6^2^.8^2^] cage as *t-kaa*, [4^2^.8^4^] cage as *t-ste*, [4^4^.6^4^.12^2^] cage as *t-sty*, and [8^4^.12^2^] cage) compose UZIF-33 (Figure 5b). To the best of our knowledge, UZIF-33 has the highest number of tiles (8 types) within known ZIF topologies. Among these tiles, the [6^3^] cage as *t-kah* and [4^4^.6^4^.12^2^] cage as *t-sty* were first observed in ZIF structures, while the [8^4^.12^2^] cage was novel and not even present in zeolites. 12-MRs as the largest pore opening were enclosed by alternating the [4^4^.6^4^.12^2^] cage (*t-sty*) and novel [8^4^.12^2^] cage.

The unique structural configurations of UZIF-31 and UZIF-33 emerge because of how the layers stack together, which are made up of different tiles. Specifically, UZIF-31 exhibits an A–A′ stacking pattern (Figure 5c), whereas UZIF-33 displays an A-B-A stacking pattern (Figure 5d). Furthermore, there is a notable difference in the structural arrangement due to the ordering pattern of the *t-cub* ([4^6^] cage) tile, illustrating the dissimilarity between UZIF-31 and UZIF-33 (Figures S35 and S36). The framework density, denoted as *T*/*V* (the number of T sites per volume), for UZIF-31 (*T*/*V* = 2.33 nm^–3^) and UZIF-33 (*T*/*V* = 2.42 nm^–3^, identical to UZIF-32) is comparable to that of an iconic ZIF, ZIF-8 (*T*/*V* = 2.45 nm^–3^).

**Figure 6:**
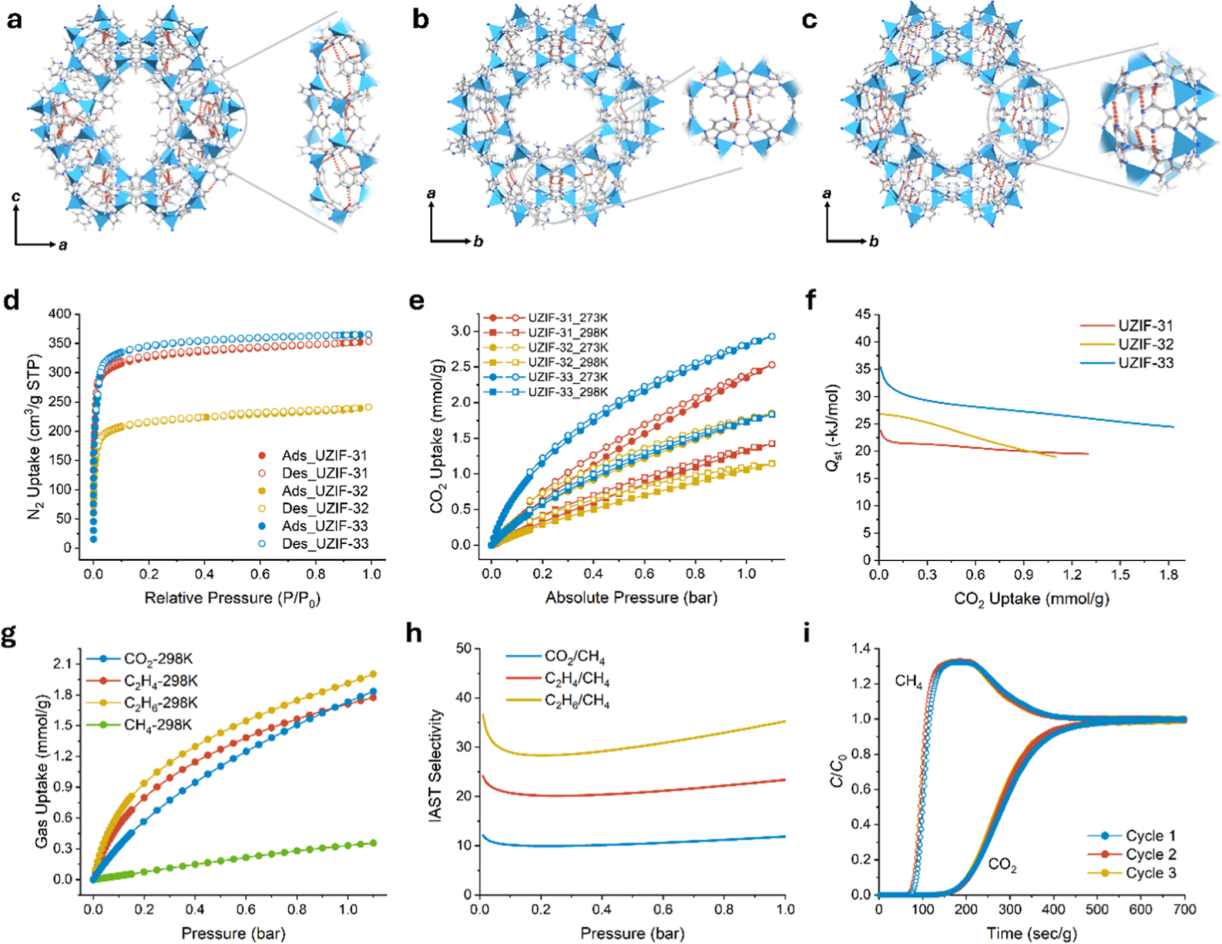
Hydrogen bonds (red dotted line; within 3.0 Å) in the crystal structure of new ZIFs. (a) The infinite chain of hydrogen bonds along the [010] direction is highlighted in UZIF-31. (b) The hydrogen bond between the adjacent *t-cub* cages is highlighted in UZIF-32. (c) A cluster of hydrogen bonds in a *t-lau* cage is highlighted in UZIF-33. Blue polyhedron, Zn; gray, C; blue, N; white, H. (d) N_2_ sorption isotherms for ZIFs measured at 77 K. (e) CO_2_ sorption isotherms for ZIFs measured at 273 K (circle symbol) and 298 K (square symbol). (f) The *Q*_st_ of CO_2_ for ZIFs. (g) Experimental adsorption isotherms of CO_2_, C_2_H_4_, C_2_H_6_, and CH_4_ for UZIF-33 measured at 298 K. (h) Predicted IAST selectivities of CO_2_/CH_4_, C_2_H_4_/CH_4_, and C_2_H_6_/CH_4_ for UZIF-33 with equimolar mixtures (50/50) at 298 K. (i) Dynamic breakthrough curves for 50/50 mixtures of CO_2_ and CH_4_ at 298 K and 1 bar in a column packed with UZIF-33.

The crystal structures of the new ZIFs confirm the link–link interactions attributed to hydrogen bonds. Notably, weak hydrogen bonds^72^ between C(sp^2^)–H as donors and N(C=N–C) as acceptors within 3.0 Å were monitored in coordinated Im linkers (Figure S37). In UZIF-31, an infinite hydrogen bond chain was evident along the [010] direction, contributing to a total of 60 hydrogen bonds in a unit cell (the number of hydrogen bonds per volume; *H*/*V* = 2.91 nm^–3^) (Figures 6a and S38). The pair of hydrogen bonds between adjacent *t-cub* cages along the [010] direction was observed in UZIF-32 (*H*/*V* = 0.81 nm^–3^) (Figures 6b and S39). In UZIF-33 (*H*/*V* = 1.82 nm^–3^), a cluster of hydrogen bonds within a *t-lau* cage bolstered the crystal structure (Figures 6c and S40). The highest *H*/*V* value and infinite hydrogen bond chain among the three ZIFs were found in UZIF-31, influencing its chemical stability under varying aqueous conditions with pH levels (Figures S25–S27).

Although the frameworks of T-50 and T12 are energetically stable, the calculations were performed by using unsubstituted imidazolates, which do not align with the synthetic results of UZIFs that consist of a combination of Im and AzbIm. To support our synthetic results, we conducted further DFT optimization for exchanged AzbIm, specifically 4-AzbIm and 5-AzbIm, in UZIF-31 with *uft*1 topology (Table S14). The resulting frameworks were named UZIF-31_4-AzbIm and UZIF-31_5-AzbIm, respectively. These frameworks have the same chemical formula (Zn_48_C_432_N_240_H_336_), but their ligand compositions differ. The calculated total energy per atom (eV/atom) for the UZIF-31_4-AzbIm framework is lower than that for UZIF-31_5-AzbIm (−6.913 eV/atom vs −6.905 eV/atom). This result supports the notion that 4-AzbIm is the key linker for the synthesis of UZIF-31 (*uft*1). A similar approach was applied to UZIF-32 with an *uft*2 topology, resulting in UZIF-32_4-AzbIm and UZIF-32_5-AzbIm. The framework energy of UZIF-32_4-AzbIm is slightly lower than that of UZIF-32_5-AzbIm (−6.829 eV/atom vs −6.826 eV/atom). However, the energy difference for UZIF-32 (0.003 eV/atom) is smaller than that for UZIF-31 (0.008 eV/atom), which could explain the formation of UZIF-32 (*uft*2) from 5-AzbIm. A faster computational screening process with varying linker compositions may lead to more effective targeted synthetic protocols.

### Porosity and Selective Adsorption

The permanent porosity of new ZIFs, along with their microporosity attributed to well-defined 12-MR channel-type pores, was validated through the N_2_ sorption at 77 K (Figure 6d). The Brunauer–Emmett–Teller (BET) surface areas were measured as 1291(2), 832(2), and 1371(6) m^2^ g^–1^ for UZIF-31, −32, and −33, respectively (Figures S41–S43). The pore size distribution indicated the presence of pores with diameters of 1.19 nm in UZIF-31, 1.27 nm in UZIF-32, and 1.36 nm in UZIF-33 (Figures S44 and S45). Notably, among the trio of new ZIFs, UZIF-33 stood out with the highest BET surface area of 1376 m^2^ g^–1^, surpassing all previously reported AzbIm-based ZIFs (Table S16).

To explore the potential applications in separating harmful gases, we examined the adsorption behavior of CO_2_ gas on ZIFs under ambient conditions (Figures 6e and S47–S49). Among these three ZIFs, UZIF-33 exhibited the highest capacity for CO_2_ adsorption, followed by UZIF-31 and then UZIF-32. This sequence directly correlated with the number of exposed nitrogen atoms per unit volume, denoted as *N*/*V* (nm^–3^): UZIF-32 (1.82 nm^–3^), UZIF-31 (2.62 nm^–3^), and UZIF-33 (3.63 nm^–3^). The highest *N*/*V* value in UZIF-33 suggests a strong interaction between the frameworks and CO_2_ gas molecules due to a more polar pore surface originating from a large number of uncoordinated nitrogen atoms. UZIF-33 also showed the highest isosteric heat of adsorption (*Q*_st_ = −35.1 kJ mol^–1^) for CO_2_ compared to those of the other two ZIFs (Figure 6f). Further experiments were conducted to explore selective adsorption behaviors, particularly in separating CO_2_ from CH_4_, a vital industrial process for enhancing natural gas quality through carbon capture, contributing to a carbon-neutral society. As anticipated, UZIF-33 demonstrated significantly enhanced uptake capacities for CO_2_ (1.73 mmol g^–1^), C_2_H_4_ (1.71 mmol g^–1^), and C_2_H_6_ (1.91 mmol g^–1^), which were around five times higher than that for CH_4_ (0.33 mmol g^–1^) (Figures 6g and S50). Based on the ideal adsorbed solution theory (IAST),^76^ the selectivity of equimolar (50/50) CO_2_/CH_4_, C_2_H_4_/CH_4_, and C_2_H_6_/CH_4_ mixtures at 298 K and 1 bar were determined as 11.2, 23.4, and 35.3, respectively (Figure 6h). The outstanding separation efficiency of UZIF-33 in isolating C_2_ gases and CO_2_ from CH_4_ was attributed to its highly polar pore environment. Intriguingly, the CO_2_/CH_4_ selectivity value of 11.9 is the highest among those of various reported ZIFs (Table S23). To demonstrate the capture of CO_2_ of UZIF-33 for practical applications, we conducted dynamic gas adsorption breakthrough experiments using CO_2_/CH_4_ gas mixtures at 298 K and 1 bar with a flow rate of 5 mL min^–1^ (see the details in the Supporting Information). The adsorption performance of UZIF-33 was assessed through breakthrough curve measurements conducted over three cycles. Notably, these breakthrough curves revealed no significant decline in adsorption capacity even after 120 s g^–1^ of CO_2_ retention time (Figure 6i). Consequently, the dynamic breakthrough experiments demonstrated the remarkable ability of UZIF-33 to effectively separate CO_2_ from CH_4_.

## Conclusions

In conclusion, we have developed an effective search algorithm for exploring new ZIFs by using a combination of structural descriptors, including the O–T–O angle difference, vertex symbol, and T–O–T angle. Through this method, we have assessed the synthesizability of 420 trinodal ZIFs, establishing a collection of ZIFs with a high level of feasibility (90 ZIFs in Tier 1), from over 4 million zeolite structures. By introducing hydrogen bonds as link–link interactions between Im linkers, we successfully synthesized three novel ZIFs, UZIF-31 (*uft*1), UZIF-32 (*uft*2), and UZIF-33 (*uft*2), with novel topologies that are classified within Tier 1 synthesizable ZIFs. Notably, UZIF-33 exhibited an exceptional ability to separate CO_2_ from CH_4_, making it a potential candidate for applications in carbon neutrality due to its highly polar pore surface. This data-driven discovery, which combines the digitization of chemical intuitions with targeted synthesis, has the potential to contribute significantly to the development of new functional materials and unravel challenges in the field of porous materials. In the forthcoming years, the automation of synthesis processes^77^ is expected to further accelerate the creation and evaluation of new ZIFs, merging computational predictions with real-world applications.^78^

## Methods

### Measurement of T–O–T Angles in ZIF

T–O–T angles in ZIFs were measured from single-crystal data of a representative 38 ZIFs in 38 topologies. Tetrahedral metals such as Zn and centroids of imidazolate were regarded as T and O, respectively.

### Analysis of the Zeolite Structures

All handling zeolite crystal structures were downloaded as cif files from http://www.iza-structure.org/databases/ (for IZA zeolites) and http://www.hypotheticalzeolites.net (for hypothetical zeolites). These zeolites are composed of SiO_2_, and their frameworks are energy-minimized structures. O–T–O and T–O–T angles of all zeolites were measured from the cif files.

### Building ZIF Structures

A total of 420 hypothetical ZIF structures were generated using the ToBaCCo code^59^ based on 420 parent zeolite topologies. The topological information for crystal construction comprises tetrahedral coordination symmetry, number of vertices and edges types, unit cell vectors, and fractional coordinates of vertices and edges. The unit cell vectors are parent zeolite unit cell vectors, and the fractional coordinates of the vertices and edges are the coordinates of Si and O, respectively, in the parent zeolite. Zn as the metal node and Im as the organic linker were used as the building blocks.

### Energy Calculation of ZIFs

As generated, the hypothetical ZIFs are not in their minimum-energy structures (”geometries”). Along with the experimentally known ZIFs, they were subjected to molecular mechanics (MM) optimization as implemented in the LAMMPS package^61^ with the force field MOF–FF whose parameters were fit to the ZIF.^60^ The optimization was done while simultaneously relaxing the unit cell parameters and the atomic coordinates within each unit cell, with the convergence criterion set for the 2-norm (length) of the global (all-atom) force vector being smaller than 10^–4^ kcal mol^–1^ Å^–1^. In line with the procedure of Lewis et al.,^64^ the data is plotted as the MM energies per Zn atom (relative to that of the MM-optimized ZIF with the zni topology) and the number of Zn atoms per volume of the optimized cell.

### Synthesis of UZIF-31 (***uft*1**)

Zinc(II) nitrate hexahydrate (29.7 mg, 0.1 mmol), Im (30.0 mg, 0.44 mmol), and 4-AzbIm (13.2 mg, 0.11 mmol) were dissolved in 2.0 mL of DMF in a 5 mL vial. The vial was sealed and heated at 120 °C for 72 h. Transparent crystals were obtained and washed with DMF and acetone 3 times, respectively.

### Synthesis of UZIF-32 (***uft*2**)

Zinc(II) nitrate hexahydrate (29.7 mg, 0.1 mmol), Im (30.0 mg, 0.44 mmol), and 5-AzbIm (12.0 mg, 0.1 mmol) were dissolved in 2.0 mL of DMF in a 5 mL vial. The vial was sealed and heated at 120 °C for 24 h. Clear solution of the mixture was stored at room temperature for 48 h. Transparent hexagonal prismatic crystals were obtained and washed with DMF and acetone 3 times, respectively.

### Synthesis of UZIF-33 (***uft*2**)

Zinc(II) nitrate hexahydrate (29.7 mg, 0.1 mmol), Im (26.6 mg, 0.39 mmol), and Pur (13.2 mg, 0.11 mmol) were dissolved in 1.5 mL of DMF in a 5 mL vial. The vial was sealed and heated to 120 °C for 96 h. Transparent hexagonal prismatic crystals were obtained and washed with DMF and acetone 3 times, respectively.

## Data Availability

Crystallographic information files (T-*n*_zeolitecode.cif) of optimized 420 hypothetical ZIFs are available in the Supporting Information. Crystallographic data for structures reported in this article have been deposited at the Cambridge Crystallographic Data Centre (CCDC), under deposition numbers 2285519 (UZIF-31), 2285520 (UZIF-32), and 2285521 (UZIF-33). These data can be obtained free of charge from The Cambridge Crystallographic Data Centre via www.ccdc.cam.ac.uk/data_request/cif.

## Supporting Information Available

Materials and general procedures; detailed experimental and computational methods; and characterization of UZIFs including ^1^H NMR, PXRD, SCXRD, and gas adsorption (PDF)
UZIF-31 (CIF)
UZIF-32 (CIF)
UZIF-33 (CIF)
CIF files for hypothetical ZIFs (ZIP)

## Supplementary Material

au5c00077_si_001.pdf

au5c00077_si_002.cif

au5c00077_si_003.cif

au5c00077_si_004.cif
