# Supplementary material for: Data-Driven Search Algorithm for Discovery of Synthesizable Zeolitic Imidazolate Frameworks
Source: JACS Au. 2025 Mar 7;5(3):1460–70. doi: 10.1021/jacsau.5c00077 (PMC11938011; doi:10.1021/jacsau.5c00077)
Supplement: Supplementary file 1 — au5c00077_si_001.pdf [file au5c00077_si_001.pdf]

Supporting Information

Data-Driven Search Algorithm for Discovery of Synthesizable Zeolitic Imidazolate Frameworks

Soochan Lee[1], Hyein Jeong[1], Sungyeop Jung[1], Yeongjin Kim[1], Eunchan Cho[1], Joohan Nam[1], D. ChangMo Yang[1], Dong Yun Shin[2], Jung-Hoon Lee[2,3], Hyunchul Oh[1,4], and Wonyoung Choe*[,1,4,5]

[1]Department of Chemistry, Ulsan National Institute of Science and Technology, Ulsan 44919, Republic of Korea.

[2]Computational Science Research Center, Korea Institute of Science and Technology (KIST), Seoul 02792, Republic of Korea

[3]KU-KIST Graduate School of Converging Science and Technology, Korea University, Seoul 02841, Republic of Korea

[4]Graduate School of Carbon Neutrality, Ulsan National Institute of Science and Technology, Ulsan 44919, Republic of Korea.

[5]Graduate School of Artificial Intelligence, Ulsan National Institute of Science and Technology, Ulsan 44919, Republic of Korea.

*Corresponding author: choe@unist.ac.kr

Supporting experimental procedures

Measurement of T-O-T angles in ZIF. T-O-T angles in ZIFs were measured from single-crystal data of representative 38 ZIFs in 38 topologies. Tetrahedral metal such as Zn and centroid of imidazolate were regarded as T and O, respectively.

Analysis of the zeolite structures. All handling zeolite crystal structures were downloaded as cif files from http://www.iza-structure.org/databases/ (for IZA zeolites) and http://www.hypotheticalzeolites.net (for hypothetical zeolites). These zeolites are composed of SiO_2, and their frameworks are energy-minimized structures. O-T-O and T-O-T angles of all zeolites were measured from the cif files.

Building ZIF structures. A total of 420 hypothetical ZIF structures were generated using ToBaCCo code[1] based on 420 parent zeolite topologies. The topological information for crystal construction comprises tetrahedral coordination symmetry, number of vertices and edges types, unit cell vectors, and fractional coordinates of vertices and edges. The unit cell vectors are parent zeolite unit cell vectors, and fractional coordinates of the vertices and edges are the coordinates of Si and O, respectively, in the parent zeolite. Zn as the metal node and Im as the organic linker were used as the building blocks.

Energy calculation of ZIFs. As generated, the hypothetical ZIFs are not in their minimum-energy structures (”geometries”). Along with the experimentally known ZIFs, they were subjected to molecular mechanics (MM) optimization as implemented in the LAMMPS package[2] with the force field MOF-FF whose parameters were fit to ZIF[3]. The optimization was done while simultaneously relaxing the unit cell parameters and the atomic coordinates within each unit cell, with the convergence criterion set for the 2-norm (length) of the global (all-atom) force vector being smaller than 10^{-4} kcal mol^{-1} Å^{-1}. In line with the procedure of Lewis *et al.*[4], the data is plotted as the MM energies per Zn atom (relative to that of the MM-optimized ZIF with the **zni** topology) and the number of Zn atoms per volume of the optimized cell.

For the assessment of cell volume changes in known ZIFs, we applied both FF and density functional theory (DFT) calculations, as demonstrated by testing against experimental cell volumes. DFT calculations were done using the Vienna Ab initio Simulation Package (VASP)[5,6], where the electrons and nuclei are set to interact with the projector augmented-wave pseudopotentials[7]. The electronic orbitals were constructed using the plane-wave basis set, with kinetic energy cutoff at 600 eV. Due to the relatively low density of ZIFs, sampling the energy at a single k-point (Γ) was observed to be sufficient for convergence to within 0.1 kJ/mol per atom. For the energy functional, we used the Perdew-Becke-Ernzerhof (PBE) form[8,9], added with Grimme's D3 dispersion correction[10] and Becke-Johnson (BJ) damping[11-14].

Independently from the MM optimization, the experimentally known ZIFs were also subjected to optimization by the extended tight binding (xTB) method[15] with the GFN2 parametrization[16], as implemented in the DFTB+ package[17]. The energies were calculated (”sampled”) only at the Γ point of the Brillouin zone.

Here, the optimization is set to partially relax the unit cell parameters and the atomic coordinates while preserving space groups, using external routines provided by the Atomic Simulation Environment (ASE)[18]. The convergence criterion is set for the maximum value of the force magnitude on every atom being smaller than 10^{-4} atomic units (Hartrees per Bohr radius).

To assess the investigate thermodynamic stability of functionalized ZIFs, we performed first-principles DFT calculations using the projector augmented wave (PAW) method[7,19] the PBE functional[8] with the VASP[5,6,20,21]. To include the effect of the van der Waals (vdW) dispersive interactions on energetics, we performed structural relaxations with Grimme's D3 correction as implemented in the VASP[14]. For all calculations, we used the following computational parameters: (i) a 450 eV plane-wave cutoff energy, (ii) a 1 x 2 x 2 k-point grid for UZIF-31_4-AzbIm (*uft*1) and UZIF-31_5-AzbIm (*uft*1), and (iii) a 2 x 1 x 2 k-point grid for UZIF-32_4-AzbIm (*uft*2) and UZIF-32_5-AzbIm (*uft*2). We explicitly treated twelve valence electrons for Zn ($3d^{10}4s^2$), five for N ($2s^22p^3$), four for C ($2s^22p^2$), and one for H($1s^1$). Using above input parameters, we relaxed internal coordinates while fixing the lattice parameters. The ions were relaxed until the force is less than 0.01 eV $Å^{-1}$.

Pore geometry screening. The geometrical surface area, pore-limiting diameter (PLD), and largest cavity diameter (LCD) of the ZIFs were calculated using Zeo++[22] with a probe radius of 1.82 Å, equivalent to the kinetic diameter of N_2.

Materials & Instruments. All reagents were accessible from the commercials and used without any further purification. *N*,*N*-Dimethylformamide (DMF) was purchased from JUNSEI chemicals. Zinc(II) nitrate hexahydrate was purchased from Sigma-Aldrich. Imidazole (Im), 4-azabenzimidazole (4-AzbIm), and 5-azabenzimidazole (5-AzbIm) were purchased from Alfa Aesar. Purine (Pur) was purchased from Acros Organics. Powder X-ray diffraction (PXRD) data were acquired by Bruker D2 phaser diffractometer (Cu Kα, 1.54184 Å), with a step size of 0.02° in 2θ. Thermogravimetric analysis (TGA) data were collected by an SDT Q600 from TA Instrument. Gas sorption isotherms were collected on Micromeritics ASAP 2020 instrument. ^{1}H-NMR spectra were collected on a 400 MHz Agilent Fourier transform-nuclear magnetic resonance (FT-NMR) spectrometer.

Synthesis of UZIF-31 (*uft*1). Zinc(II) nitrate hexahydrate (29.7 mg, 0.1 mmol), Im (30.0 mg, 0.44 mmol), and 4-AzbIm (13.2 mg, 0.11 mmol) were dissolved in 2.0 mL of DMF in a 5 mL vial. The vial was sealed and heated at 120 °C for 72 h. Transparent crystals were obtained and washed with DMF and acetone 3 times, respectively.

Synthesis of UZIF-32 (*uft*2). Zinc(II) nitrate hexahydrate (29.7 mg, 0.1 mmol), Im (30.0 mg, 0.44 mmol), and 5-AzbIm (12.0 mg, 0.1 mmol) were dissolved in 2.0 mL of DMF in a 5 mL vial. The vial was sealed and heated at 120 °C for 24 h. Continuous heating over 48 h resulted in the formation of the ZIF with **cag**

topology. Clear solution of the mixture was stored at room temperature for 48 h. Transparent hexagonal prismatic crystals were obtained and washed with DMF and acetone 3 times, respectively.

Synthesis of UZIF-33 (*uft2*). Zinc(II) nitrate hexahydrate (29.7 mg, 0.1 mmol), Im (26.6 mg, 0.39 mmol), and Pur (13.2 mg, 0.11 mmol) were dissolved in 1.5 mL of DMF in a 5 mL vial. The vial was sealed and heated at 120 °C for 96 h. Transparent hexagonal prismatic crystals were obtained and washed with DMF and acetone 3 times, respectively.

Gas sorption experiments. N_2 sorption isotherms were collected at 77 K. CO_2, C_2H_4, C_2H_6, and CH_4 sorption isotherms were collected at 273 K and 298 K. The synthesized samples (~50 mg) were washed with DMF (10 mL, 3 times) for 2 d and exchanged with acetone (10 mL, 3 times) for 2 d. The solvent-exchanged samples were activated at 120 °C for 12 h under ultra-high vacuum. Apparent Brunauer-Emmett-Teller (BET) surface area of all ZIFs were calculated using N_2 adsorption data in a chosen relative pressure range to fulfil the criteria suggested by Rouquerol et al[23].

NMR analysis. Activated samples were completely digested to detect ^{1}H-NMR spectra in 0.6 mL of a deuterated solvent (580 μL of DMSO-d_6 and 20 μL of DCl solution (35 wt. % in D_2O)).

Thermogravimetric analysis (TGA). TGA experiments for activated ZIFs were performed with scan rate 10 °C min^{-1} under air flow.

Single-Crystal X-ray Diffraction Data. X-ray diffraction data of single crystals of UZIF-31, UZIF-32, and UZIF-33 were obtained at the Pohang Accelerator Laboratory (PAL), Korea. Data of UZIF-31 was collected at 6D beamline (2019-1st-6D-A018) at 100 K with synchrotron radiation source (λ = 0.65303 Å). Data of UZIF-32 and UZIF-33 were collected at 11C beamline (2020-3rd-11C-033) at 100 K with synchrotron radiation source (λ = 0.68880 Å). All crystal structures were solved by the direct method and refined by SHELXTL[24] in the Olex2[25] program package. All hydrogen atoms were refined using the riding model. Due to highly disordered nature in the crystal structures, imidazolates were treated with rigid bond constraints. AzbIms and Pur as ligand exhibited the positional disorder between C and N atoms in the six-membered rings. EXYZ and EADP commands were treated to these atoms. Solvent masking in the Olex2 program package was employed to account for occupied highly disordered solvent molecules in the cavities of the ZIF[26,27]. The data can be obtained free of charge from the Cambridge Crystallographic Data Centre with number of CCDC 2285519 (UZIF-31), 2285520 (UZIF-32), and 2285521 (UZIF-33).

Structural information of zeolites and ZIFs

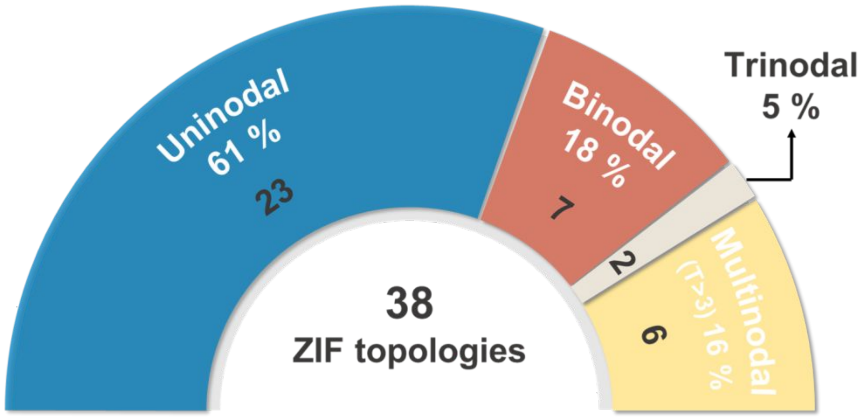

Figure S1. The number of topologies corresponding to node numbers in 38 ZIFs.

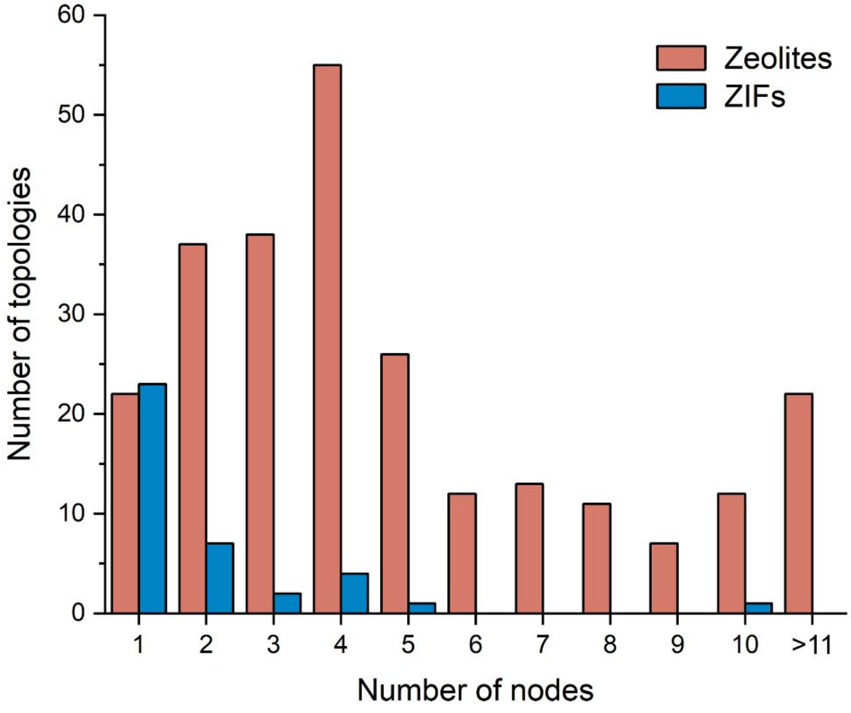

Figure S2. The number of topologies corresponding to node numbers in 255 IZA zeolites (red) and 38 ZIFs (blue).

Table S1. Zeolite codes for 38 ZIF topologies from experimental IZA and hypothetical zeolite databases.

Topology name	Number of nodes	Zeolite code
**pcb**	1	ACO(66_2_3799)
**lta**	1	LTA(225_1_1)
**neb**	1	5_2_24374
**rho**	1	RHO(217_1_4)
**cag**	1	61_2_8903
**cha**	1	CHA(166_2_148)
**gis**	1	GIS(88-2_1_28)
**kfi**	1	KFI(224_2_1379)
**dia**	1	19_1_4
**gme**	1	GME(190_2_2033)
**can**	1	CAN(62_3_6071122)
**ict**	1	167-2_1_1322
**mer**	1	MER(137_2_149)
**dft**	1	DFT(136_1_3)
**zni**	1	88_2_35192
**dia-c**[1]	1	-
**sod**	1	SOD(161_2_5322)
**crb**	1	BCT(82_1_15)
**lcs**	1	167-2_1_761
**qtz**	1	145_1_30
**afi**	1	AFI(175_2_314)
**ana**	1	ANA(206_1_151)
**atn**	1	ATN(12_2_26630)
**bam**	2	191_2_7
**moc**[2]	2	-
**gcc**	2	194_4_41205
**frl**	2	55_3_73108
**zec**	2	74_3_2009088
**afx**	2	AFX(194_2_31)
**mog**	2	15_2_196702
**zeb**	3	74_3_1465413
**ucb**	3	225_3_10
**zea**	4	-
**ykh**	4	-
**coi**	4	-
**poz**	4	139_4_52351
**nog**	5	-
**moz**[2]	10	-

[1]interpenetrated **dia** net

[2]partially 3-connected net

Table S2. Vertex symbols in 38 ZIF topologies.

Topology name	Number of nodes	Vertex symbols
**pcb**	1	4.8(2).4.8(2).4.8(2)
**lta**	1	4.6.4.6.4.8
**neb**	1	6.6.6.6(2).6(2).6(2)
**rho**	1	4.4.4.6.8.8
**cag**	1	4.6(2).6.6.6.6
**cha**	1	4.4.4.8.6.8
**gis**	1	4.4.4.8(2).8.8
**kfi**	1	4.4.4.8.6.8
**dia**	1	6(2).6(2).6(2).6(2).6(2).6(2)
**gme**	1	4.4.4.8.6.8
**can**	1	4.6.4.6.6.6
**ict**	1	6.6(2).6.6(2).6.8(4)
**mer**	1	4.4.4.8(2).8.8
**dft**	1	4.4.6(2).8(3).6(2).8(3)
**zni**	1	4.6.6.6(3).6(2).12(40)
**dia-c**	1	6(2).6(2).6(2).6(2).6(2).6(2)
**sod**	1	4.4.6.6.6.6
**crb**	1	4.6(2).6.6.6.6
**lcs**	1	6.6.6(2).6(2).6(2).6(2)
**qtz**	1	6.6.6(2).6(2).8(7).8(7)
**afi**	1	4.6(2).6.6(3).6(2).6(3)
**ana**	1	4.4.6.6.8(4).8(4)
**atn**	1	4.6.4.6.6.8
**bam**	2	4.4.4.8.6.8 / 4.4.4.8.4.8
**moc**	2	4.8(4).8(3).8(3).8(3).8(3) / 4.8(2).8(2)
**gcc**	2	4.6.4.8.4.8 / 4.4.4.8.4.8
**frl**	2	6.6.6(2).6(2).8(2).8(2) / 4.6.4.6.6.8(2)
**zec**	2	6.6.6(2).6(2).6(2).6(2) / 4.6.4.6(3).6.10(2)
**afx**	2	4.4.4.8.6.8 / 4.4.4.8.6.8
**mog**	2	4.4.6(2).6(2).8(2).8(2) / 4.8(6).6.6.6.6

**zeb**	3	4.4.4.6.4.10 / 4.6.4.10(3).4.10(5) / 4.4.4.6.10.12
**ucb**	3	4.4.4.8.6.6 / 4.4.4.12.6.6 / 4.4.4.6.4.12
**zea**	4	3.4.4.12(3).10.12(2) / 3.4.4.10.12(2).16 / 3.12(5).4.4.12(2).16 / 4.4.4.4.10.10
**ykh**	4	3.4.4.10.12(2).14(3) / 3.12(4).4.4.12(2).14(3) / 3.4.4.12(2).10.12(2) / 4.4.4.4.10.10
**coi**	4	4.8(3).4.8(4).8(9).10 / 4.8(5).4.8(7).8(4).10(3) / 4.4.4.8(7).8.8(2) / 4.4.4.10(5).8.8
**poz**	4	3.8.4.4.10.12(2) / 3.4.4.10(2).10.12(2) / 3.4.4.10(3).10.12(2) / 4.4.4.10(2).4.12(4)
**nog**	5	5.5.6.8.6.8(2) / 5.5.5.6.8.8(6) / 4.6.5.5.5.8(3) / 4.8(3).5.5(2).8(2).8(2) / 5.5.5.5.8.8(2)
**moz**	10	3.4.4.12(2).12(2).12(2) / 3.12(3).4.4.12(2).12(2) / 3.4.4.12(3).12(2).* / 3.12(2).4.4.12.12(2) / 3.4.4.12(2).12.12 / 3.4.4.12(2).12(2).* / 4.4.4.4.12.* / 3.12(5).4.4.12(2).* / 3.4.4.12(3).12.12(2) / 3.4.12(2)

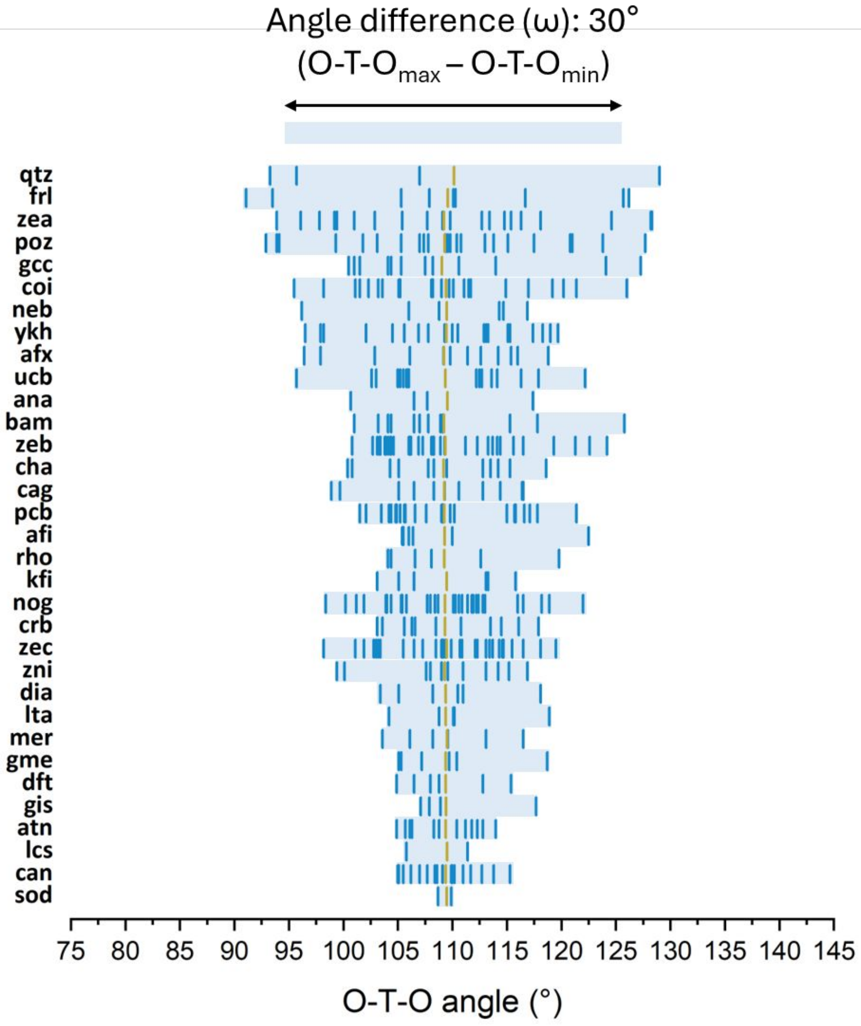

Figure S3. O−T−O (T = tetrahedral metal nodes in ZIFs) angle distributions of experimental ZIFs. Defined ω is an O−T−O angle difference between O−T−O_{max} and O−T−O_{min} (°) of tetrahedral node, respectively. Cut-off is defined as ω < 30°.

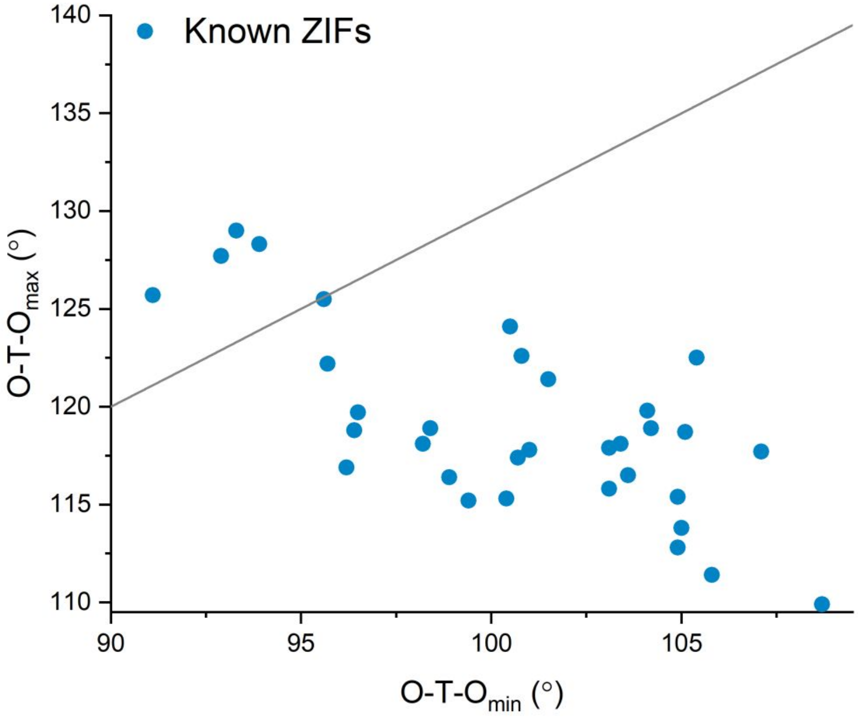

Figure S4. O−T−O angle difference (ω) in 33 known ZIFs. Defined grey line is defined as ω = 30°; under the line (ω < 30°) and upper the line (ω > 30°).

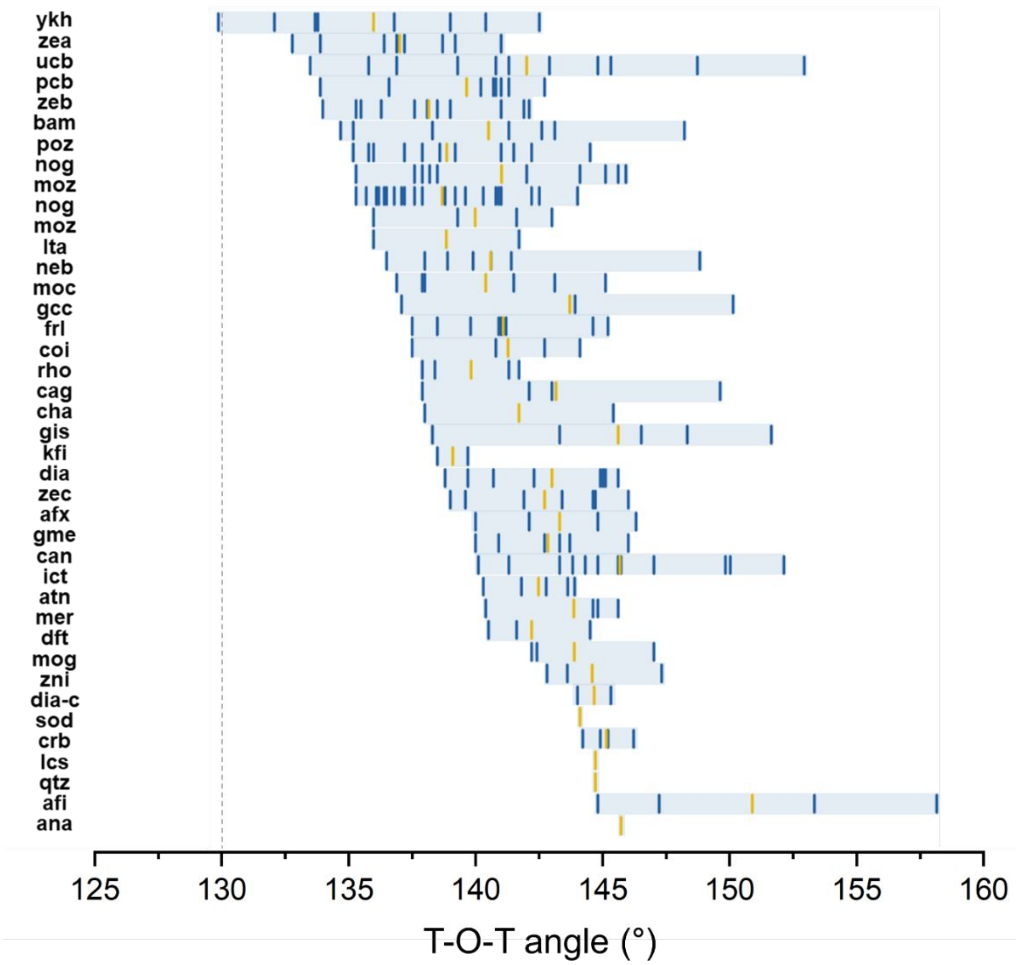

Figure S5. T−O−T (T = tetrahedral metal nodes in ZIFs) angle distribution of ZIFs collected in 38 topologies. The yellow bar represents the average T−O−T angle. Graph and data were taken from ref. 28.

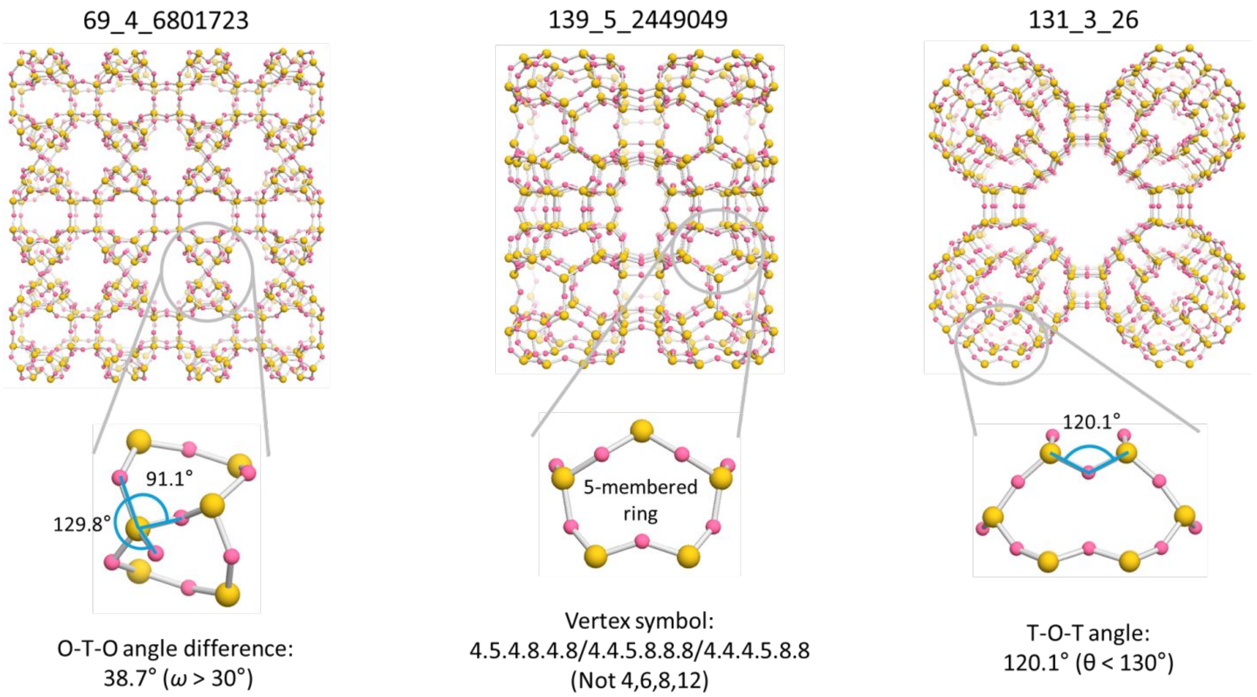

Figure S6. The illustration of representative zeolites that do not meet each criterion: O-T-O angle difference, vertex symbol, and T-O-T angle.

Computational screening and energy calculations

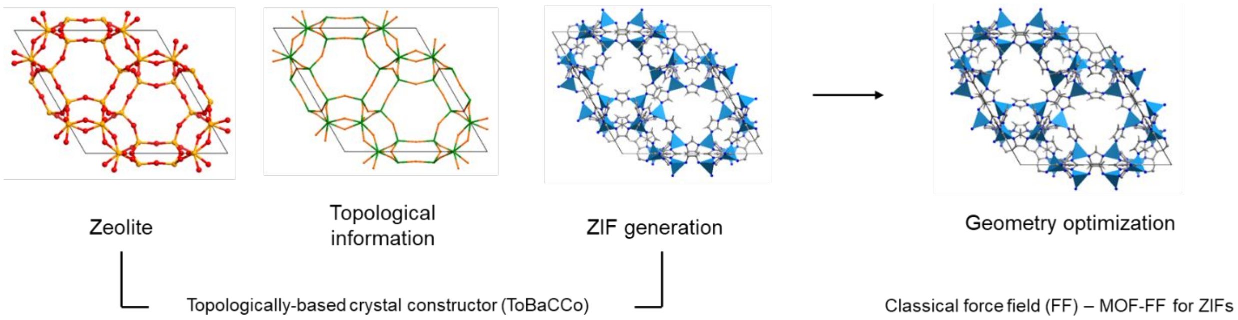

Figure S7. Graphical illustration of *in silico* design of hypothetical ZIF and geometry optimization.

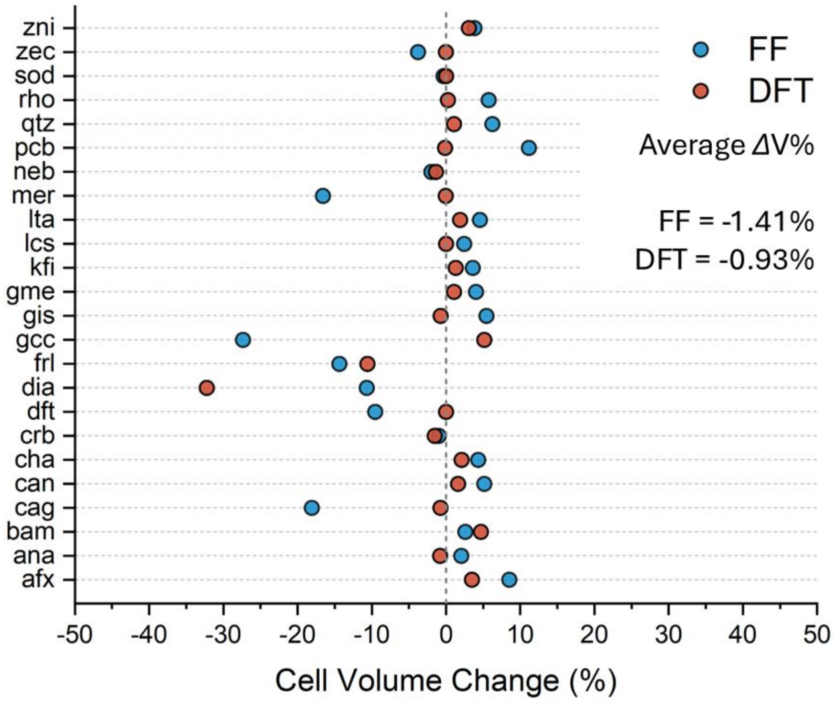

Figure S8. The percentage of cell volume change from experimental cell (dotted gray line) using geometry optimization by FF (MOF-FF for ZIFs) and DFT (VASP). DFT data was taken from ref. 28.

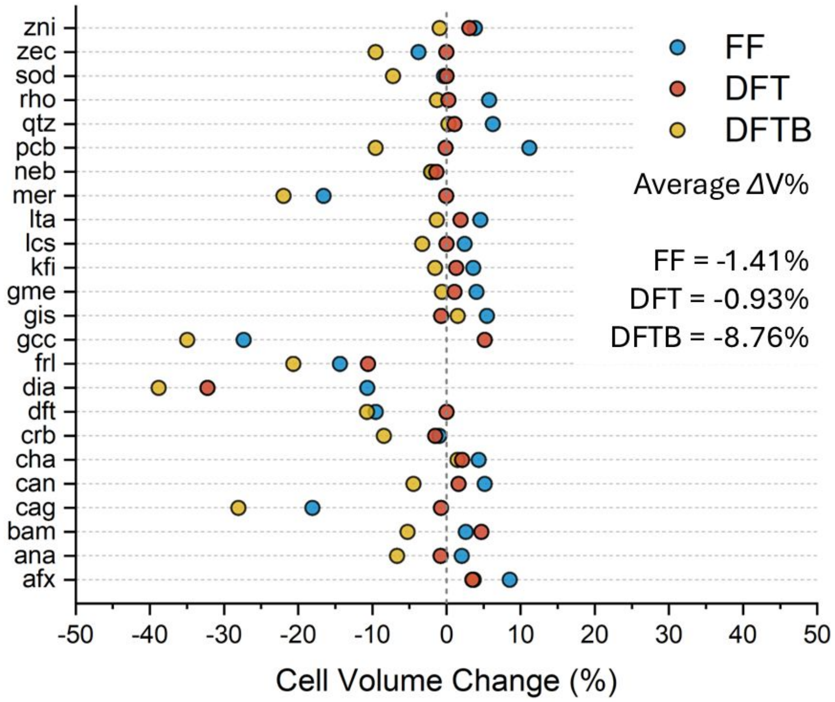

Figure S9. The percentage of cell volume change from experimental cell (dotted gray line) using geometry optimization by FF (MOF-FF for ZIFs), DFT (VASP), and DFTB (GFN2-xTB). DFT data was taken from ref. 28.

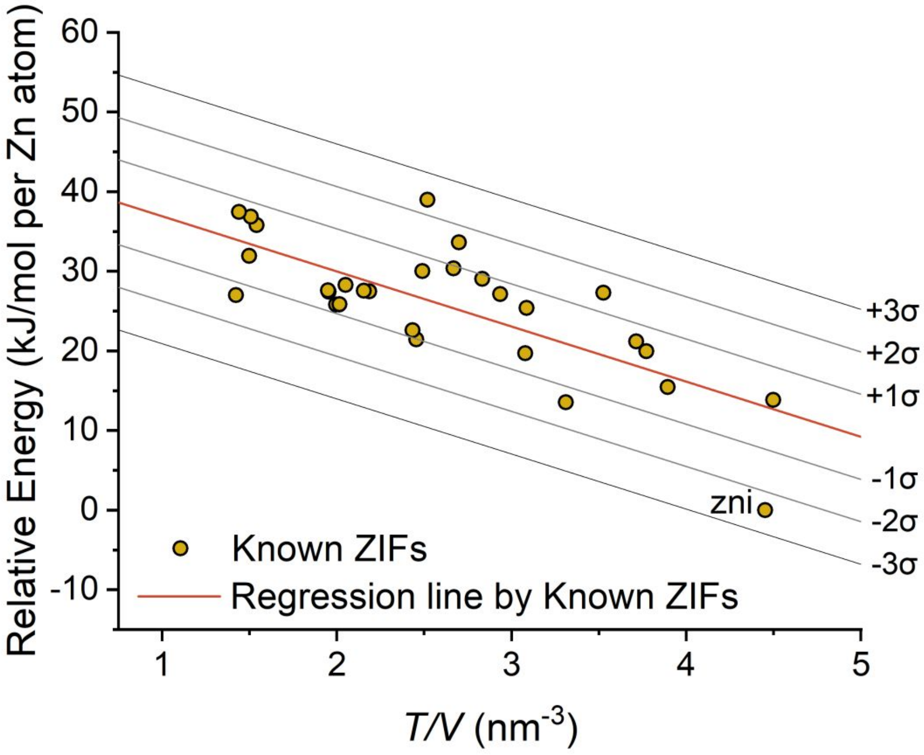

Figure S10. Plot of relative framework energy versus density for 30 known ZIF topologies optimized by MOF-FF for ZIFs. The red line represents the regression line set by known ZIF topologies for prediction intervals.

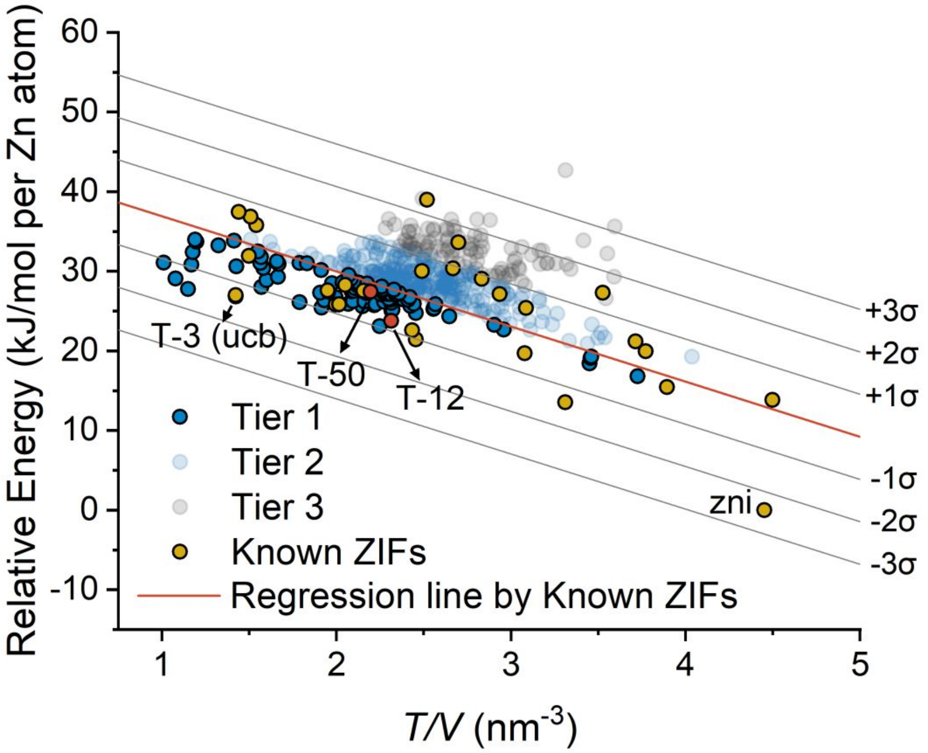

Figure S11. Plot of framework energy versus density for 420 f-ZIFs and 30 known ZIF topologies optimized by MOF-FF for ZIFs. Tier 1 (90/420; T-1 to T-90), Tier 2 (233/420; T-91 to T-323), and Tier 3 (97/420; T-324 to T-420) were divided in the section between the prediction intervals. Realized topologies in this study, T-12, and T-50, are denoted as red dots. T-3 is identical to the realized **ucb** topology.

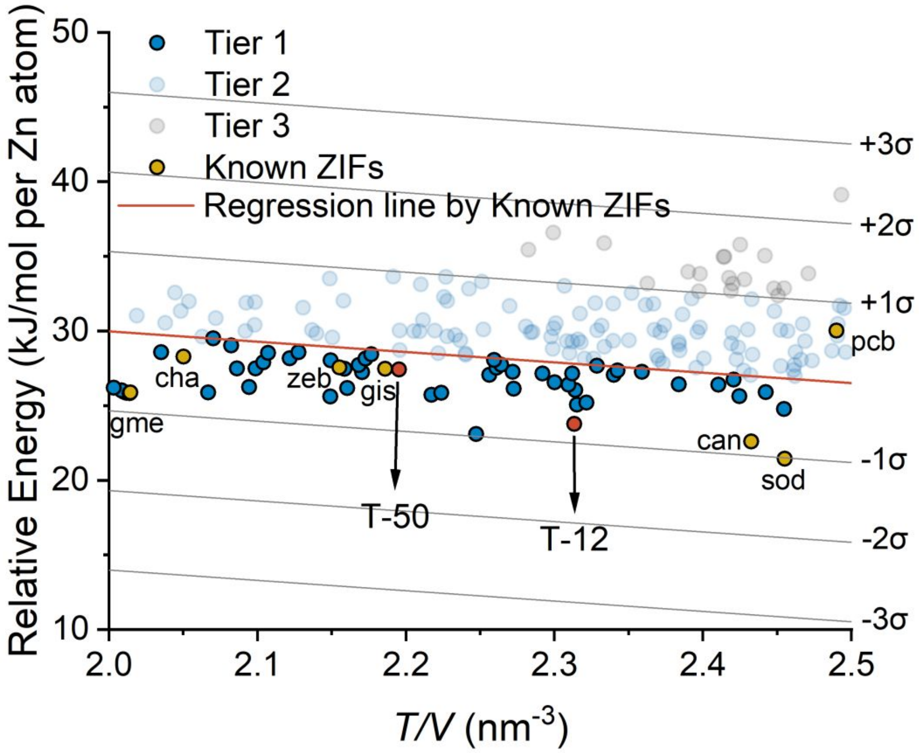

Figure S12. Highlighted “synthesis zone” (2.0 < *T/V* < 2.5 nm^{-3}) with realized ZIF topologies, T-12 and T-50, and known ZIF topologies, **gme**, **cha**, **zeb**, **gis**, **can**, **sod**, and **pcb**.

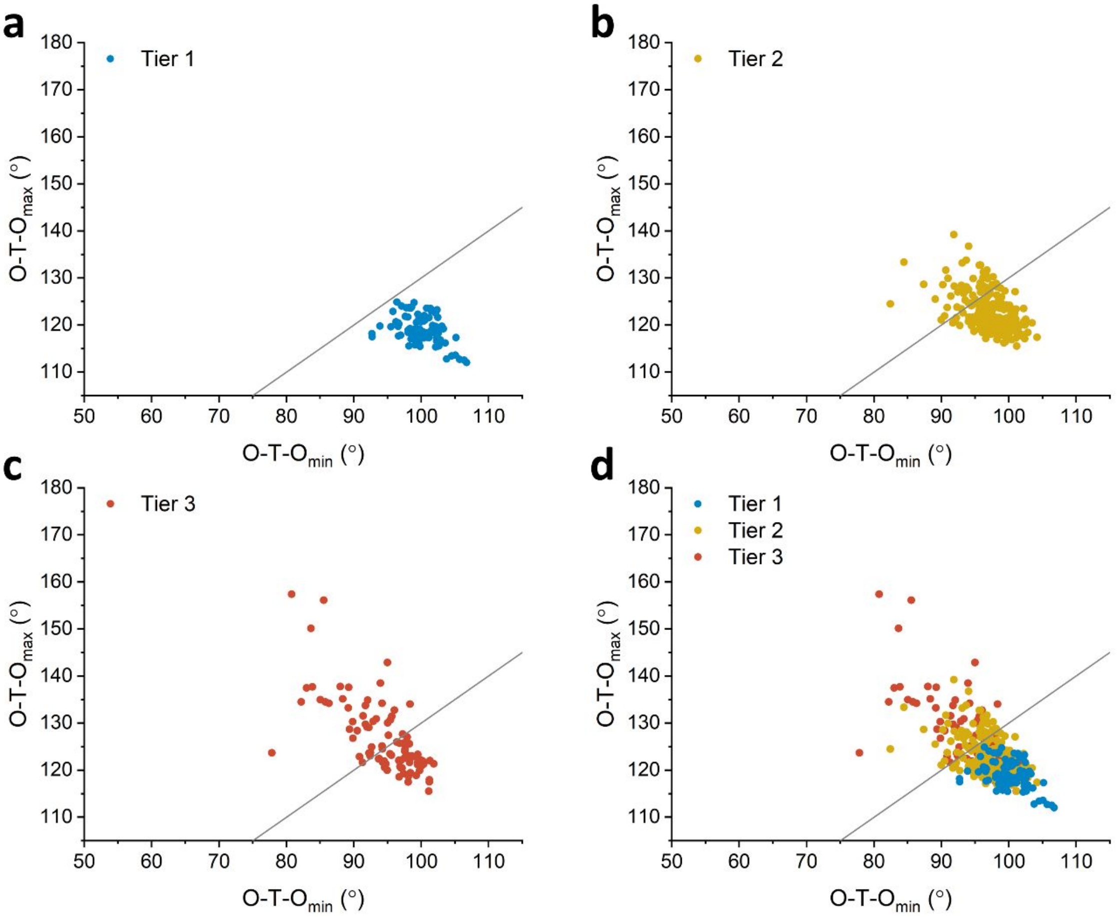

Figure S13. The O−T−O angle difference (ω) in 420 f-ZIFs with synthesizable Tiers. (a) 90 Tier 1 ZIFs are involved in ω < 30°. (b) Tier 2, (c) Tier 3, and (d) combined Tier 1, 2, and 3.

Table S3. 23 f-ZIFs (17 in Tier 1) with topologies in international zeolite association (IZA) zeolite and reticular chemistry structure resource (RCSR) database.

Name	Topology code (IZA or RCSR)	Synthesizability ranking (Tier)	Name	Topology code (IZA or RCSR)	Synthesizability ranking (Tier)
T-3	**ucb**[a]	3(1)	T-45	MSO	45(1)
T-5	**reo-t**	5(1)	T-57	UEI	57(1)
T-6	ATS(**rad**)	6(1)	T-75	AFV	75(1)
T-7	BPH	7(1)	T-84	OWE	84(1)
T-10	AFS	10(1)	T-85	OSI	85(1)
T-13	AFT	13(1)	T-99	**cmc**	99(2)
T-14	AEI	14(1)	T-186	**cfd**	186(2)
T-16	SAV	16(1)	T-223	AWO	223(2)
T-21	SFW	21(1)	T-235	AEL	235(2)
T-22	**gcd**	22(1)	T-364	AFG	364(3)
T-36	PWN	36(1)	T-389	THO	389(3)
T-44	**tdc**	44(1)			

[a] Synthesized ZIF (ZIF-412, -413, -414)

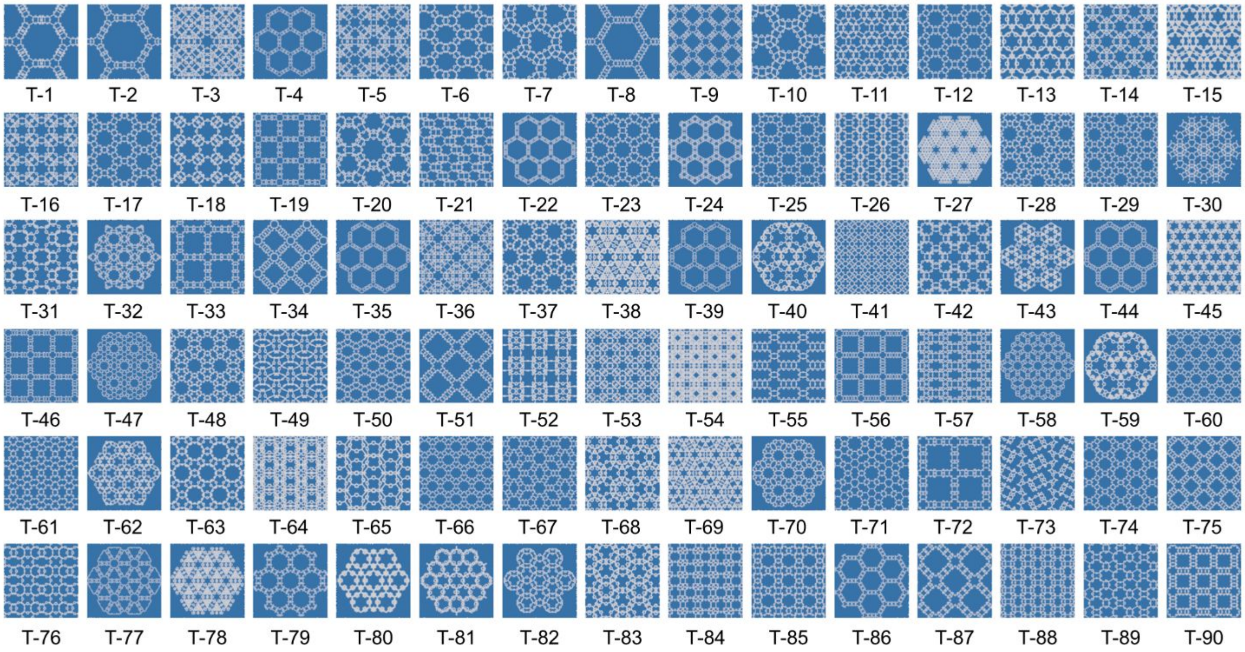

Figure S14. Tiling projections of 90 f-ZIFs in Tier 1 with the topology label.

Table S4. Structural information for 90 f-ZIFs in Tier 1.

Name	Space group	*a* (Å)	*b* (Å)	*c* (Å)	*α* (°)	*β* (°)	*γ* (°)	*T/V* (nm^{-3})
T-1_194_3_1351	P63/mmc	51.0511	51.0511	27.7471	90	90	120	1.15
T-2_194_3_58	P63/mmc	61.9245	61.9245	20.1159	90	90	120	1.08
T-3_225_3_10(**ucb**)	Fm-3m	73.9729	73.9729	73.9729	90	90	90	1.42
T-4_191_3_104	P6/mmm	63.3599	63.3599	20.4874	90	90	120	1.01
T-5_225_6_33496(**reo-t**)	Pm-3m	43.1873	43.1873	43.1873	90	90	90	1.79
T-6_62_3_6069507(ATS)	Pnma	9.9287	25.9336	41.4763	90	90	90	2.25
T-7_189_3_2152(BPH)	Amm2	25.3675	44.9006	25.6935	90	90	90	1.91
T-8_191_3_111	P6/mmm	60.2852	60.2852	19.5582	90	90	120	1.17
T-9_139_3_1383	I4/mmm	46.7589	46.7589	27.9797	90	90	90	1.57
T-10_AFS	Amm2	50.9305	44.5601	25.5814	90	90	90	1.93
T-11_194_4_47046	P63/mmc	26.8754	26.8754	76.2884	90	90	120	2.01
T-12_65_3_1955	Cmmm	26.4885	44.8641	17.4597	90	90	90	2.31
T-13_194_3_854(AFT)	P63/mmc	26.7917	26.7917	57.6226	90	90	90	2.01
T-14_AEI	Cmcm	26.8847	24.5719	36.1749	90	90	90	2.01
T-15_166_3_8971	R-3m	32.7968	32.7968	72.4907	90	90	120	1.60
T-16_SAV	P4/nmn	36.2595	36.2595	18.2241	90	90	90	2.00
T-17_194_3_73	P63/mmc	46.746	46.746	18.4076	90	90	120	2.07
T-18_139_3_1386	I4/mmm	32.2106	32.2106	48.543	90	90	90	1.91
T-19_123_3_66	P4/mmm	39.9762	39.9762	21.0564	90	90	90	1.43
T-20_191_4_3263	P6/mmm	43.6251	43.6251	27.1017	90	90	120	2.15
T-21_166_3_6775(SFW)	R-3m	26.8049	26.8049	86.9604	90	90	120	2.00
T-22_191_4_3084(**gcd**)	P6/mmm	57.5665	57.5665	28.4145	90	90	120	1.18
T-23_191_3_143	P6/mmm	45.8933	45.8933	18.8477	90	90	120	2.09

T-24_191_3_260	P6mm	40.2585	40.2585	25.6868	90	90	120	1.66
T-25_74_3_1465659	Imma	18.0152	25.2137	47.6602	90	90	90	2.22
T-26_69_3_40373	Fmmm	26.6974	34.5518	44.9538	90	90	90	2.32
T-27_166_3_10705	R-3m	33.4334	33.4334	51.642	90	90	120	2.16
T-28_191_4_3293	P6/mmm	41.978	41.978	28.2877	90	90	120	2.22
T-29_191_3_123	P6/mmm	43.5736	43.5736	18.8621	90	90	120	2.32
T-30_194_3_1130	P63/mmc	36.3626	36.3626	39.742	90	90	120	1.58
T-31_65_4_131648	Cmmm	34.1198	27.637	27.6494	90	90	90	2.45
T-32_164_3_235888	P-3m1	34.2474	34.2474	12.9968	90	90	120	2.27
T-33_139_3_411	I4/mmm	54.8999	54.8999	20.3564	90	90	90	1.56
T-34_138_3_38065129	P42/ncm	47.8256	47.8256	10.059	90	90	90	2.09
T-35_194_3_189	P63/mmc	60.8567	60.8567	18.7339	90	90	120	1.20
T-36_229_3_4866(PWN)	Im-3m	48.5393	48.5393	48.5393	90	90	90	2.10
T-37_164_3_161005	P3m1	24.8089	24.8089	29.1877	90	90	120	2.31
T-38_166_3_12659	R-3m	33.9869	33.9869	54.6564	90	90	120	1.98
T-39_191_3_489	P6/mmm	55.461	55.461	18.9236	90	90	120	1.19
T-40_194_5_3920386	P63mc	25.9972	25.9972	85.0117	90	90	120	2.17
T-41_138_3_37206266	P-1	37.0227	36.987	10.164	89.1312	90.9103	89.9852	3.45
T-42_139_3_1138p	I4/mmm	35.9336	35.9336	32.1922	90	90	90	2.31
T-43_194_6_110020894	P63/mmc	25.7683	25.7683	116.01	90	90	120	2.16
T-44_191_3_495(**tdc**)	P6/mmm	52.9544	52.9544	18.6517	90	90	120	1.32
T-45_166_5_74221169(MSO)	R-3m	33.0434	33.0434	78.5112	90	90	120	2.42
T-46_131_3_855	P42/mmc	39.661	39.661	18.2964	90	90	90	1.67
T-47_191_3_110	P6/mmm	45.0977	45.0977	19.4289	90	90	120	2.10
T-48_194_5_2762662	P63/mmc	35.441	35.441	47.9633	90	90	120	2.30

T-49_61_3_35312779	Pbca	9.8477	32.3757	20.2052	90	90	90	3.73
T-50_74_3_1812225	Imma	43.7623	18.9822	26.3214	90	90	90	2.20
T-51_139_4_47869	P4/nbm	53.2031	53.2031	14.4194	90	90	90	1.57
T-52_51_3_48024	Pnma	38.696	17.3962	13.476	90	90	90	2.65
T-53_139_4_58492	I4/mmm	33.8816	33.8816	54.7894	90	90	90	2.04
T-54_227_3_2510	Fd-3m	59.6979	59.6979	59.6979	90	90	90	2.26
T-55_65_3_475	Cmmm	37.6125	19.3733	30.3805	90	90	90	2.17
T-56_123_3_64	P4/mmm	38.1034	38.1034	19.9258	90	90	90	1.66
T-57_UEI	Fmm2	38.4698	18.4577	27.6799	90	90	90	2.44
T-58_191_3_117	P6/mmm	45.2271	45.2271	19.1567	90	90	120	2.12
T-59_194_6_110020888	P63/mmc	25.7144	25.7144	116.998	90	90	120	2.15
T-60_74_3_1492759	Pnna	17.0045	26.714	44.3268	90	90	90	2.38
T-61_59_5_22446366	P2	18.2667	24.3361	35.2003	90	90.0025	90	2.56
T-62_194_3_1684	P63/mmc	24.9375	24.9375	49.0354	90	90	120	2.27
T-63_194_3_858	P63/mmc	24.9644	24.9644	58.207	90	90	120	2.29
T-64_53_3_464381	Pmna	25.347	10.1268	27.0573	90	90	90	3.46
T-65_63_4_19632810	Cmcm	18.7656	19.7556	67.4523	90	90	90	2.56
T-66_74_3_1832918	Imma	44.6263	16.4256	27.1679	90	90	90	2.41
T-67_194_3_339	P63/mmc	44.8206	44.8206	19.6402	90	90	120	2.11
T-68_194_3_860	P63/mmc	24.9944	24.9944	57.5606	90	90	120	2.31
T-69_166_3_9998	R-3m	35.3674	35.3674	33.7054	90	90	120	2.96
T-70_191_5_63722	P6/mmm	35.016	35.016	49.9823	90	90	120	2.26
T-71_194_3_263	P63/mmc	44.8083	44.8083	19.0573	90	90	120	2.17
T-72_131_3_206	P42/mmc	39.3114	39.3114	20.0283	90	90	90	1.55
T-73_59_3_209	P21/c	10.5183	14.3352	46.837	90	100.7578	90	3.46
T-74_74_3_1803477	Imma	45.0178	16.7408	27.2182	90	90	90	2.34

T-75_AFV	P-3m1	26.5752	26.5752	23.0543	90	90	120	2.13
T-76_55_3_49834	Pbam	10.0053	50.3616	16.396	90	90	90	2.90
T-77_191_3_146	P6/mmm	47.4025	47.4025	19.3403	90	90	120	1.91
T-78_166_5_57369096	R-3m	34.2647	34.2647	39.0951	90	90	120	2.26
T-79_191_3_278	P6mm	38.0156	38.0156	26.7939	90	90	120	1.79
T-80_166_5_30848386	R-3m	26.1285	26.1285	73.1045	90	90	120	2.08
T-81_194_4_46846	P63/mmc	34.8479	34.8479	37.7097	90	90	120	2.42
T-82_194_3_1139	P63/mmc	25.7054	25.7054	57.807	90	90	120	2.18
T-83_194_3_868	P63/mmc	24.5921	24.5921	58.6841	90	90	120	2.34
T-84_63_4_21980043(OWE)	Cmcm	28.4471	36.0781	26.4352	90	90	90	2.36
T-85_63_4_1928932(OSI)	P21/m	25.2461	51.5576	9.7411	90	100.616	90	2.57
T-86_164_3_101294	P-3m1	52.4594	52.4594	10.6855	90	90	120	1.41
T-87_139_3_4161	I4mm	50.1459	50.1459	10.4216	90	90	90	1.83
T-88_119_5_51897673	I4mm	26.8905	26.8905	48.97	90	90	90	2.26
T-89_141_3_10599	I41/amd	18.6883	18.6883	118.048	90	90	90	2.33
T-90_139_5_2290331	P4/nbm	39.1419	39.1419	25.2221	90	90	90	2.07

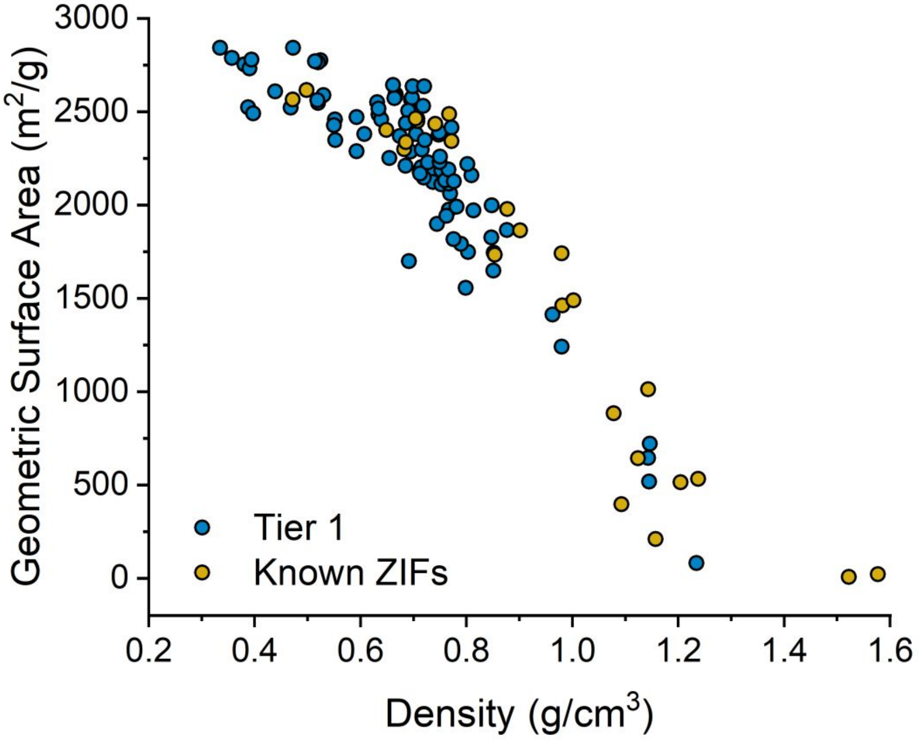

Figure S15. Plot of framework density versus geometric surface area (calculated using Zeo++) for 90 Tier 1 f-ZIFs and known ZIF topologies.

Table S5. Pore geometry information for 90 Tier 1 f-ZIFs.

Name	Density ($g\ cm^{-3}$)	Geometric surface area ($m^2\ g^{-1}$)	LCD (Å)	PLD (Å)
T-1_194_3_1351	0.38092	2752.94	37.99	37.12
T-2_194_3_58	0.35711	2787.35	48.05	47.87
T-3_225_3_10(**ucb**)	0.47188	2565.30	38.24	13.83
T-4_191_3_104	0.33493	2841.87	51.13	49.92
T-5_225_6_33496(**reo-t**)	0.59232	2471.38	32.08	7.77
T-6_62_3_6069507(ATS)	0.74459	1898.96	14.22	13.50
T-7_189_3_2152(BPH)	0.63401	2485.73	18.84	11.33
T-8_191_3_111	0.38754	2522.30	48.12	46.71
T-9_139_3_1383	0.51995	2545.92	20.00	19.33
T-10_AFS	0.63919	2458.52	17.95	12.42
T-11_194_4_47046	0.66655	2592.06	15.37	6.77
T-12_65_3_1955	0.76650	1973.56	14.39	13.26
T-13_194_3_854(AFT)	0.66599	2590.35	15.27	6.88
T-14_AEI	0.66551	2578.12	14.25	7.02
T-15_166_3_8971	0.52992	2588.80	20.23	13.04
T-16_SAV	0.66376	2571.13	16.67	7.70
T-17_194_3_73	0.68482	2209.95	17.59	14.80
T-18_139_3_1386	0.63155	2551.13	19.30	11.15
T-19_123_3_66	0.47262	2841.63	28.39	26.72
T-20_191_4_3263	0.71209	2173.25	21.44	14.86
T-21_166_3_6775(SFW)	0.66131	2642.64	15.22	6.98
T-22_191_4_3084(**gcd**)	0.39005	2731.62	45.00	43.57
T-23_191_3_143	0.69392	2286.58	18.45	14.72
T-24_191_3_260	0.55139	2458.79	30.09	26.44
T-25_74_3_1465659	0.73463	2176.38	13.94	11.76
T-26_69_3_40373	0.76706	1974.29	14.73	6.35
T-27_166_3_10705	0.71580	2296.42	15.50	5.08
T-28_191_4_3293	0.73682	2122.59	19.45	15.16
T-29_191_3_123	0.76918	2061.34	18.48	14.73

T-30_194_3_1130	0.52421	2774.91	23.10	9.97
T-31_65_4_131648	0.81331	1969.90	12.55	6.31
T-32_164_3_235888	0.75294	2110.05	17.01	13.67
T-33_139_3_411	0.51843	2561.37	25.83	24.64
T-34_138_3_38065129	0.69124	1699.55	20.04	19.77
T-35_194_3_189	0.39703	2489.80	47.69	47.06
T-36_229_3_4866(PWN)	0.69533	2564.65	19.82	7.42
T-37_164_3_161005	0.76669	2113.71	13.33	6.18
T-38_166_3_12659	0.65447	2251.43	21.93	7.03
T-39_191_3_489	0.39437	2779.27	42.45	41.48
T-40_194_5_3920386	0.71916	2146.67	14.52	7.71
T-41_138_3_37206266	1.14295	644.78	5.28	4.01
T-42_139_3_1138p	0.76521	2137.57	13.97	8.50
T-43_194_6_110020894	0.71520	2203.17	13.98	7.12
T-44_191_3_495(**tdc**)	0.43889	2608.92	39.98	38.95
T-45_166_5_74221169(MSO)	0.80335	1748.53	15.31	3.75
T-46_131_3_855	0.55260	2347.60	27.71	27.05
T-47_191_3_110	0.69712	2573.61	18.43	14.90
T-48_194_5_2762662	0.76206	1941.60	19.83	14.54
T-49_61_3_35312779	1.23440	81.91	4.29	3.07
T-50_74_3_1812225	0.72736	2226.99	13.73	12.26
T-51_139_4_47869	0.51954	2763.48	26.52	23.67
T-52_51_3_48024	0.87658	1864.71	9.14	7.37
T-53_139_4_58492	0.67429	2369.08	19.10	7.55
T-54_227_3_2510	0.74753	2376.44	20.34	4.94
T-55_65_3_475	0.71841	2529.53	12.31	7.11
T-56_123_3_64	0.54974	2426.29	26.84	24.31
T-57_UEI	0.80917	2158.93	10.35	7.58
T-58_191_3_117	0.70298	2440.70	18.43	14.72
T-59_194_6_110020888	0.71214	2167.79	14.89	7.01
T-60_74_3_1492759	0.78983	1790.87	13.81	13.08
T-61_59_5_22446366	0.84696	1826.37	10.27	6.57

T-62_194_3_1684	0.75278	2184.17	13.56	6.12
T-63_194_3_858	0.75936	2132.43	13.49	11.48
T-64_53_3_464381	1.14496	518.31	5.11	3.42
T-65_63_4_19632810	0.84799	1997.84	8.17	5.47
T-66_74_3_1832918	0.79861	1555.68	14.89	13.57
T-67_194_3_339	0.69817	2634.61	14.13	12.78
T-68_194_3_860	0.76604	2190.57	12.69	6.24
T-69_166_3_9998	0.98005	1241.05	9.64	3.18
T-70_191_5_63722	0.74914	2229.71	17.06	14.74
T-71_194_3_263	0.71992	2634.91	15.30	13.35
T-72_131_3_206	0.51383	2769.88	26.55	25.88
T-73_59_3_209	1.14613	720.42	6.05	2.11
T-74_74_3_1803477	0.77532	1816.90	15.02	12.83
T-75_AFV	0.70493	2379.41	13.61	7.13
T-76_55_3_49834	0.96251	1412.95	6.41	4.81
T-77_191_3_146	0.63387	2516.10	18.59	15.20
T-78_166_5_57369096	0.75017	2258.91	14.90	6.02
T-79_191_3_278	0.59282	2288.28	26.33	24.75
T-80_166_5_30848386	0.68992	2505.15	14.58	6.98
T-81_194_4_46846	0.80204	2218.08	9.67	6.40
T-82_194_3_1139	0.72116	2348.31	13.09	7.24
T-83_194_3_868	0.77616	2125.62	12.66	5.95
T-84_63_4_21980043(OWE)	0.78159	1990.33	11.45	6.66
T-85_63_4_1928932(OSI)	0.85077	1648.25	12.18	11.66
T-86_164_3_101294	0.46837	2521.06	38.42	38.33
T-87_139_3_4161	0.60687	2379.31	22.40	22.02
T-88_119_5_51897673	0.74856	2388.97	13.72	7.29
T-89_141_3_10599	0.77150	2414.73	9.70	5.65
T-90_139_5_2290331	0.68594	2437.11	17.92	14.06

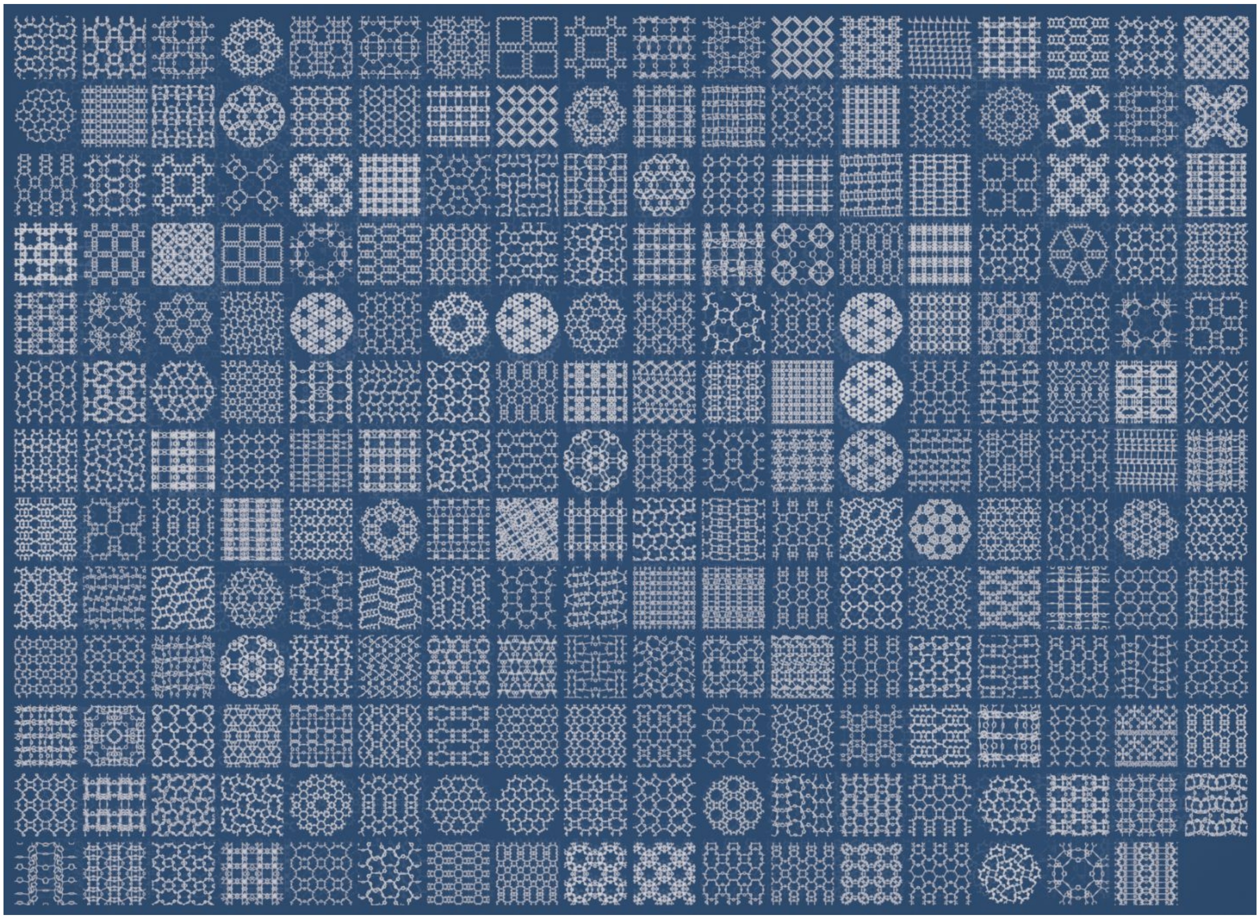

Figure S16. Schematic illustrations with tiling projections of the generated 233 f-ZIFs (from T-91 to T-323, left to right) belong to Tier 2 synthesizability

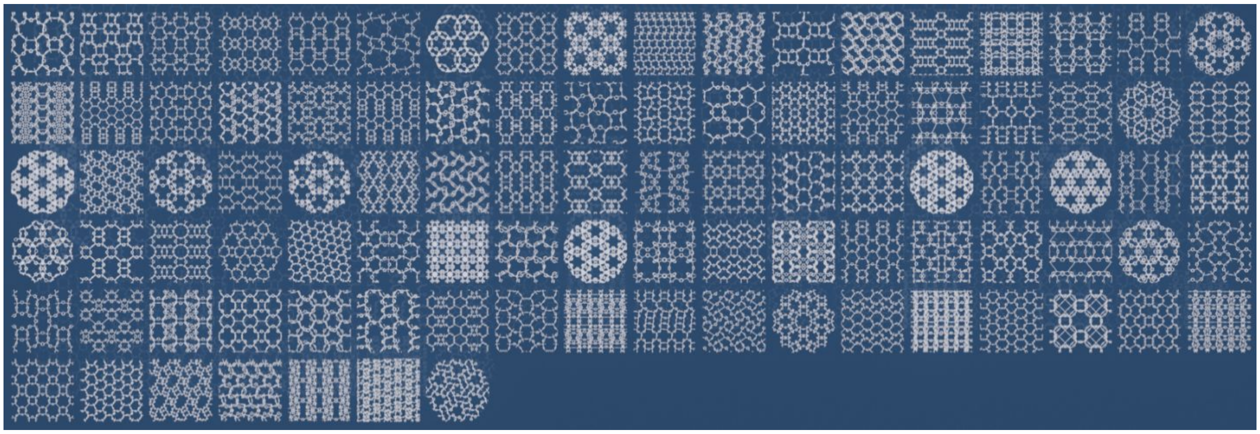

Figure S17. Schematic illustrations with tiling projections of the generated 97 f-ZIFs (from T-324 to T-420, left to right) belong to Tier 3 synthesizability

Crystal structures

Table S6. Crystallographic information of UZIF-31.

	UZIF-31
Molecular formula	$Zn_{12}N_{61}H_{79}C_{111}$
Temperature	100(2)K
Crystal system	Orthorhombic
Space group	*Imma*
Unit cell dimensions	*a* = 41.400(8) Å<br>*b* = 18.917(4) Å<br>*c* = 26.304(5) Å
V (Å^3)	20600(7) Å^3
Z	4
ρ_{calc} (g·cm^{-3})	0.984
μ (mm^{-1})	1.123
R_1, $I > 2\sigma(I)$	0.1180
wR_2, $I > 2\sigma(I)$	0.3727

Table S7. Crystallographic information of UZIF-32.

	UZIF-32
Molecular formula	$Zn_{12}N_{63}H_{71}C_{104}O_3$
Temperature	100(2) K
Crystal system	Orthorhombic
Space group	*Cmmm*
Unit cell dimensions	a = 26.899(5) Å<br>b = 43.436(9) Å<br>c = 16.964(3) Å
V (Å^3)	19821(7) Å^3
Z	4
ρ_{calc} (g·cm^{-3})	1.017
μ (mm^{-1})	1.350
R_1, $I > 2\sigma(I)$	0.1066
wR_2, $I > 2\sigma(I)$	0.3346

Table S8. Crystallographic information of UZIF-33.

	UZIF-33
Molecular formula	$Zn_{12}N_{67}H_{62}C_{92}O_3$
Temperature	100(2) K
Crystal system	Orthorhombic
Space group	*Cmmm*
Unit cell dimensions	a = 26.804(5) Å<br>b = 43.849(9) Å<br>c = 16.865(3) Å
V (Å^3)	19822(7) Å^3
Z	4
ρ_{calc} (g·cm^{-3})	0.985
μ (mm^{-1})	1.349
R_1, $I > 2\sigma(I)$	0.1175
wR_2, $I > 2\sigma(I)$	0.4114

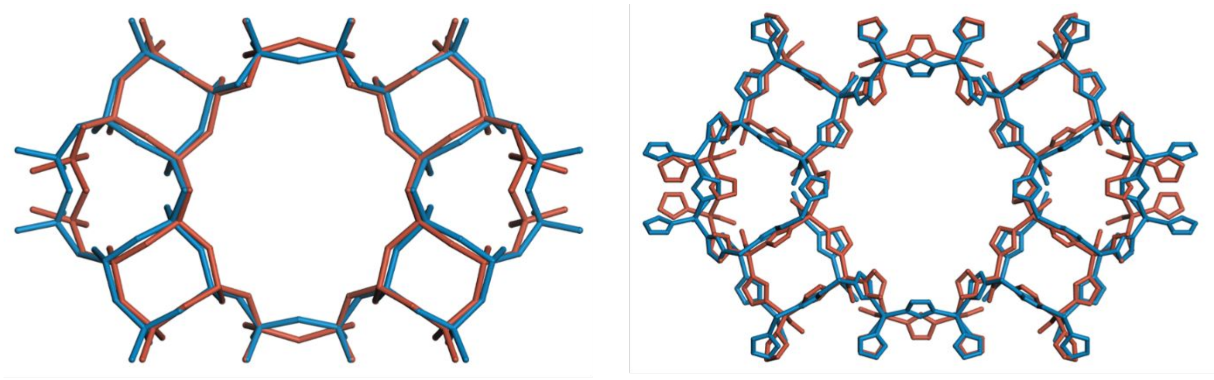

$\Delta V\% = +6.14\%$

Experimental(red)	Optimized(blue)
Imma	*Imma*
a = 41.400	*a* = 43.762
b = 18.917	*b* = 18.982
c = 26.304	*c* = 26.321
V = 20600.3	*V* = 21865.3

Figure S18. Overlay of crystal structures between experimental UZIF-31 (red) and optimized T-50 (blue). Functional groups and hydrogens are omitted for clarity.

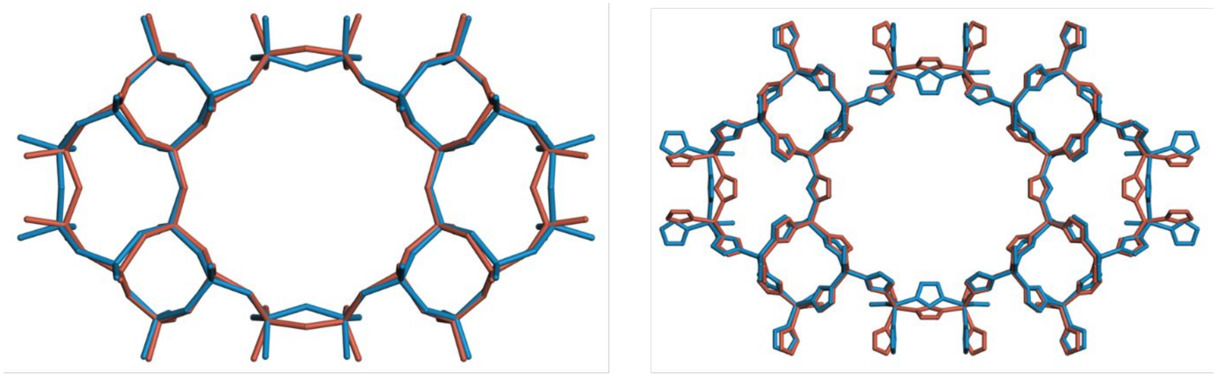

ΔV% = +4.68%

Experimental(red)	Optimized(blue)
Cmmm	*Cmmm*
a = 26.804	*a* = 26.489
b = 43.849	*b* = 44.864
c = 16.865	*c* = 17.460
V = 19821.9	*V* = 20748.8

Figure S19. Overlay of crystal structures between experimental UZIF-33 (red) and optimized T-12 (blue). Functional groups and hydrogens are omitted for clarity.

PXRD and chemical stability

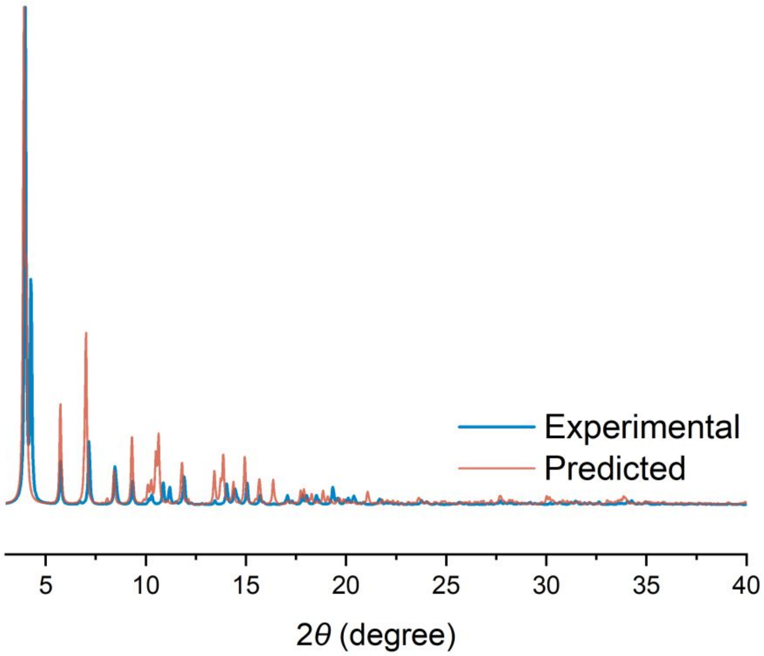

Figure S20. Comparison of PXRD diffraction patterns of experimental UZIF-31 and predicted T-50.

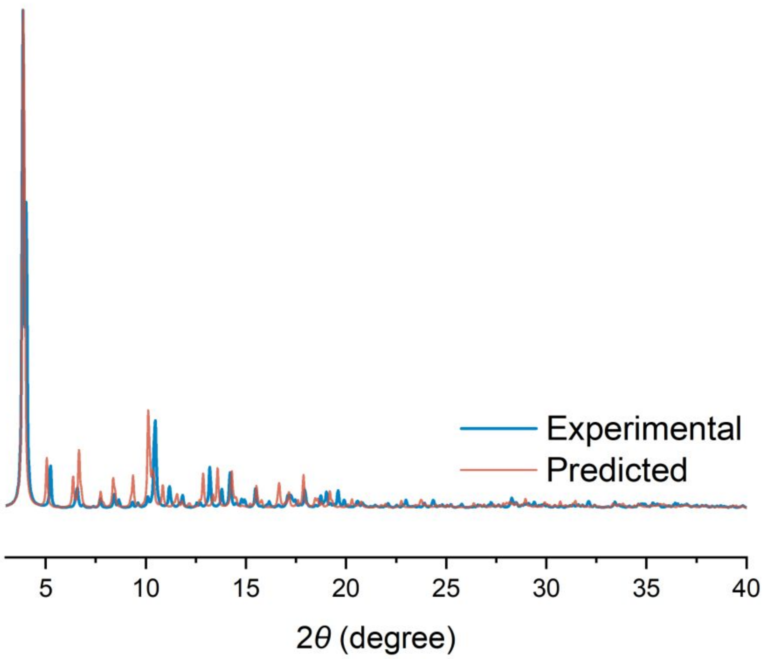

Figure S21. Comparison of PXRD diffraction patterns of experimental UZIF-33 and predicted T-12.

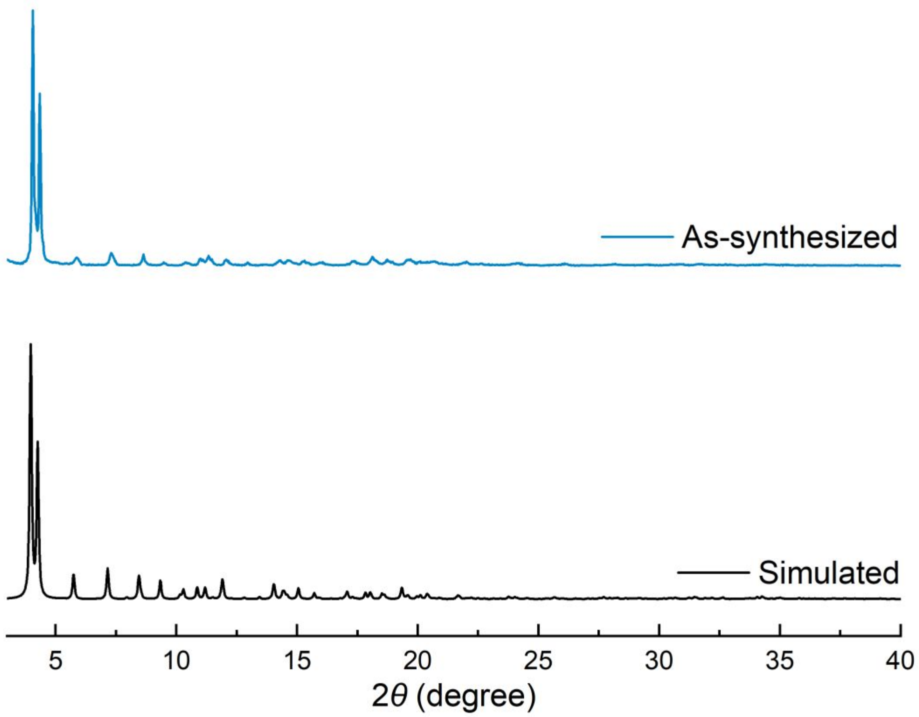

Figure S22. Comparison of PXRD diffraction patterns of as-synthesized and simulated UZIF-31.

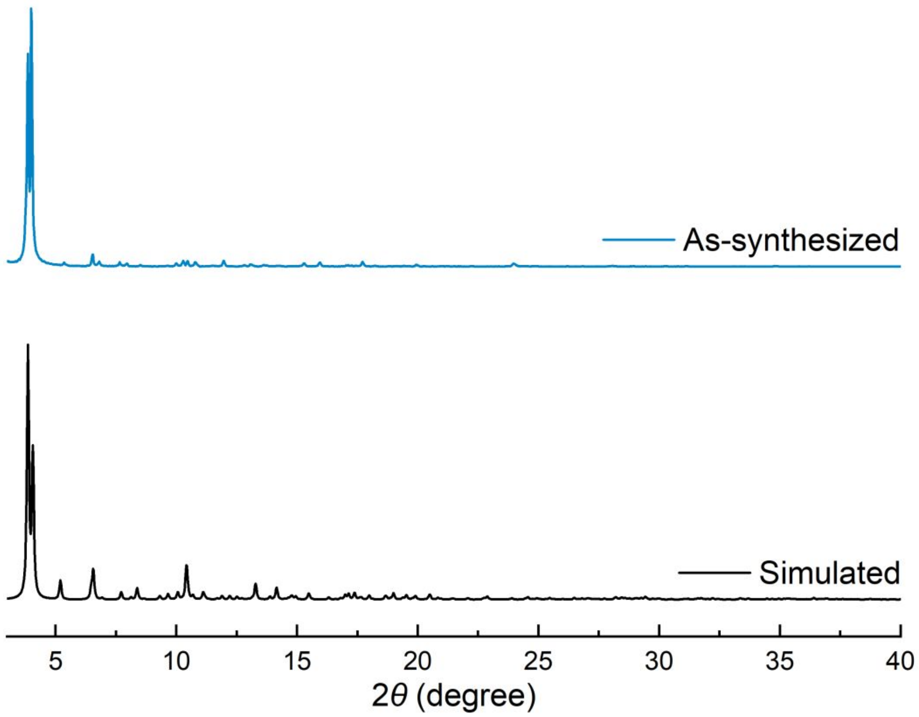

Figure S23. Comparison of PXRD diffraction patterns of as-synthesized and simulated UZIF-32.

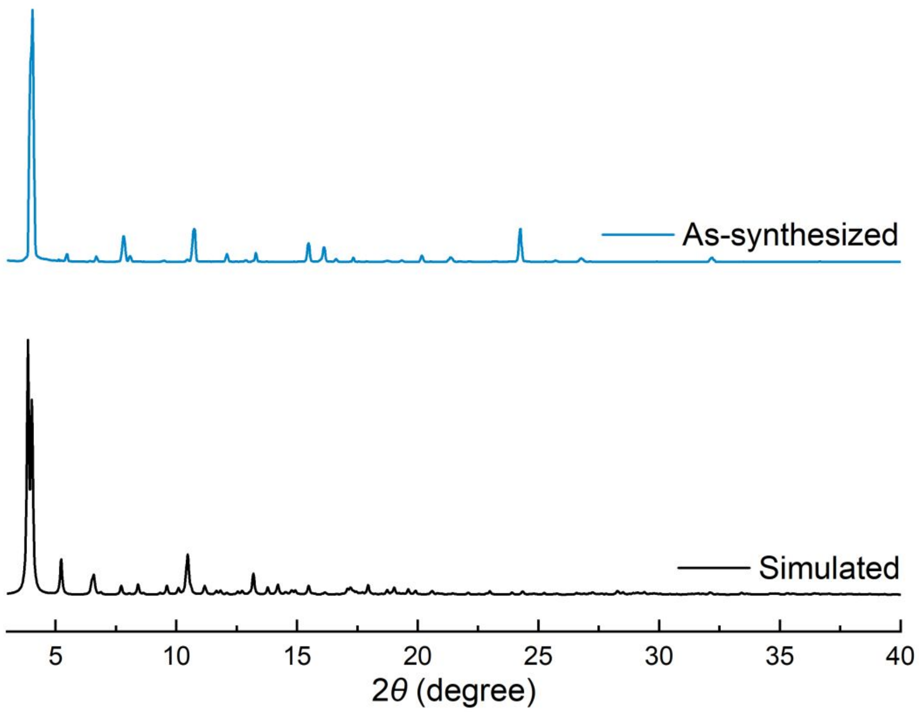

Figure S24. Comparison of PXRD diffraction patterns of as-synthesized and simulated UZIF-33.

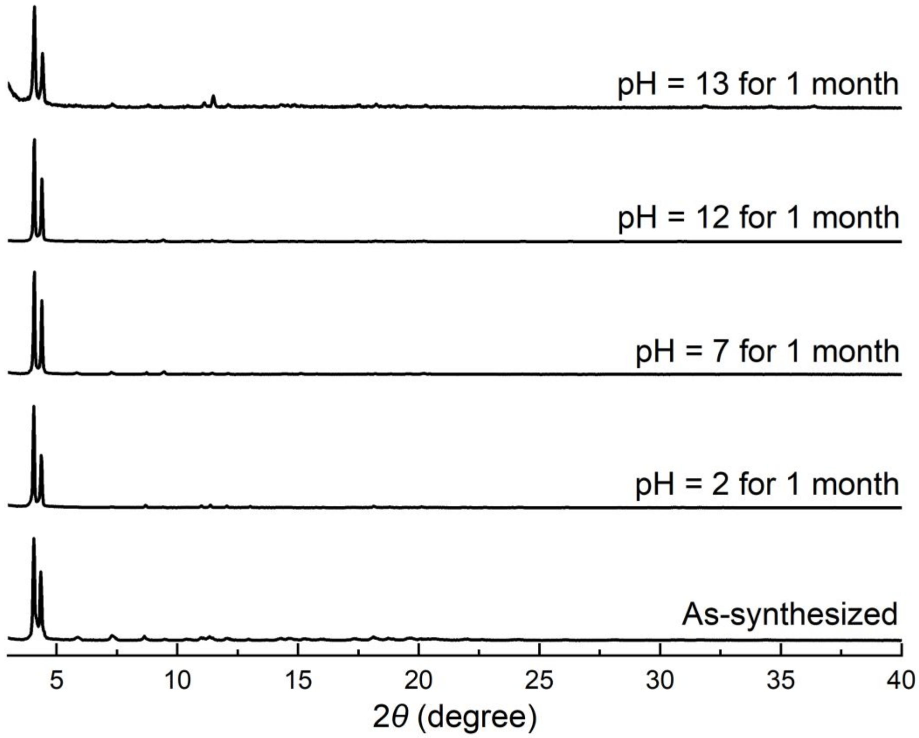

Figure S25. Comparison of PXRD diffraction patterns of as-synthesized and immersed in various pH aqueous solutions (NaOH and HCl were used to adjust pH) after 1 month for UZIF-31.

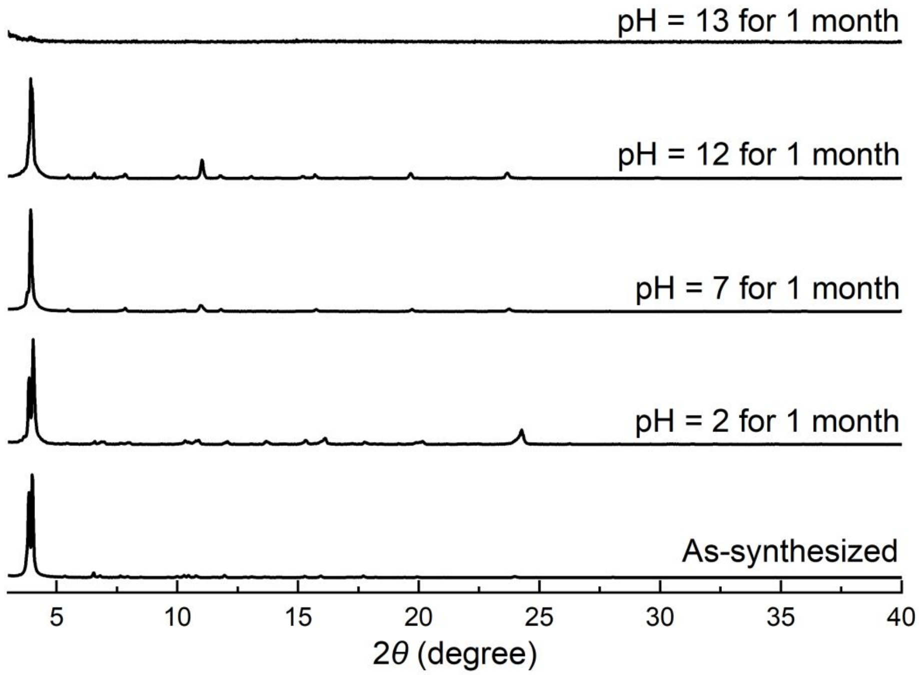

Figure S26. Comparison of PXRD diffraction patterns of as-synthesized and immersed in various pH aqueous solutions (NaOH and HCl were used to adjust pH) after 1 month for UZIF-32.

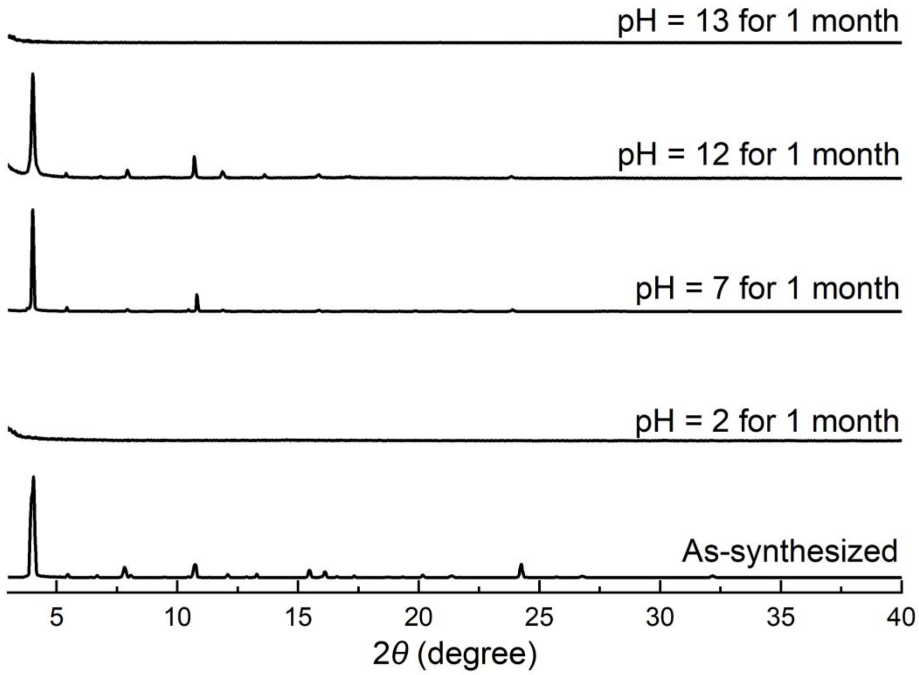

Figure S27. Comparison of PXRD diffraction patterns of as-synthesized and immersed in various pH aqueous solutions (NaOH and HCl were used to adjust pH) after 1 month for UZIF-33.

NMR spectroscopy

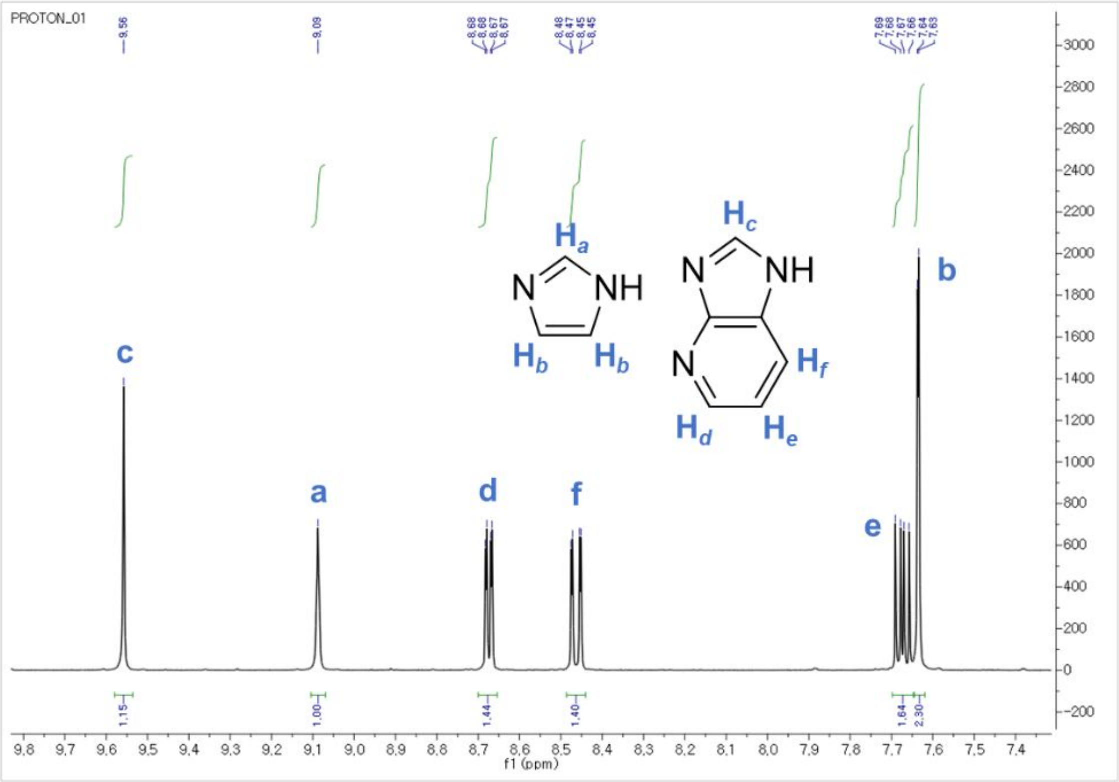

Figure S28. ^{1}H-NMR spectrum of the acid digested UZIF-31.

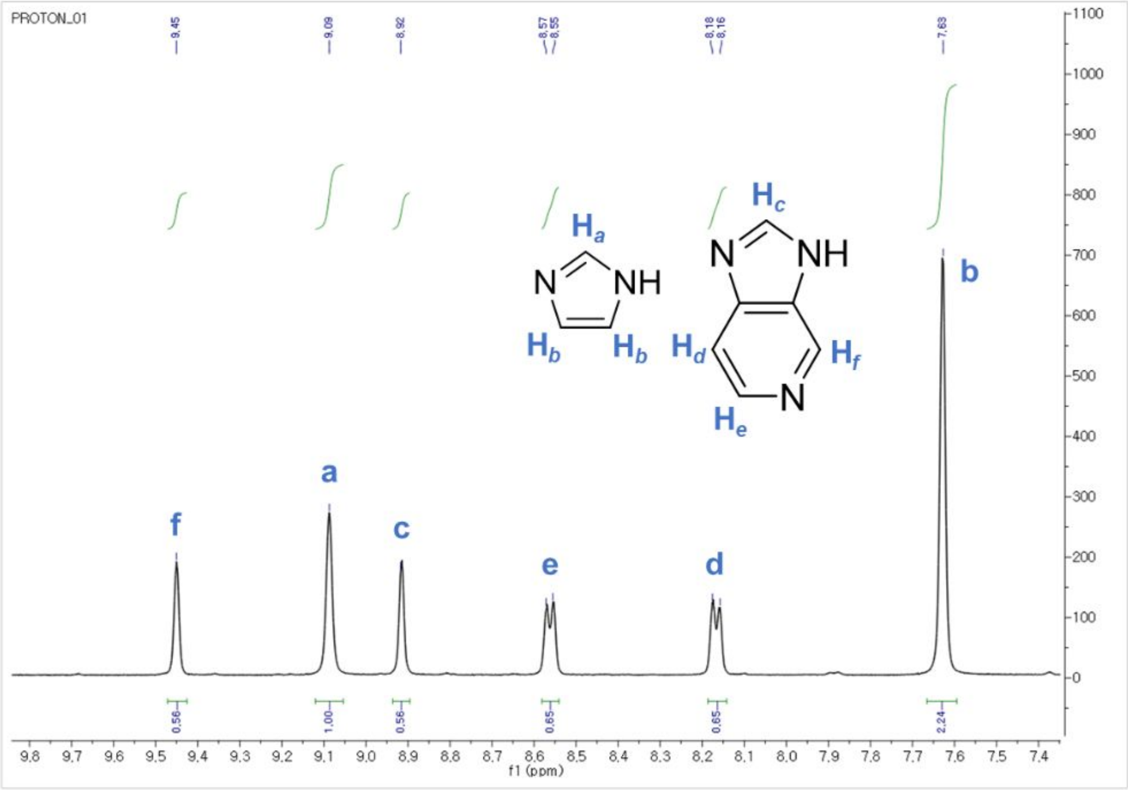

Figure S29. ^{1}H-NMR spectrum of the acid digested UZIF-32.

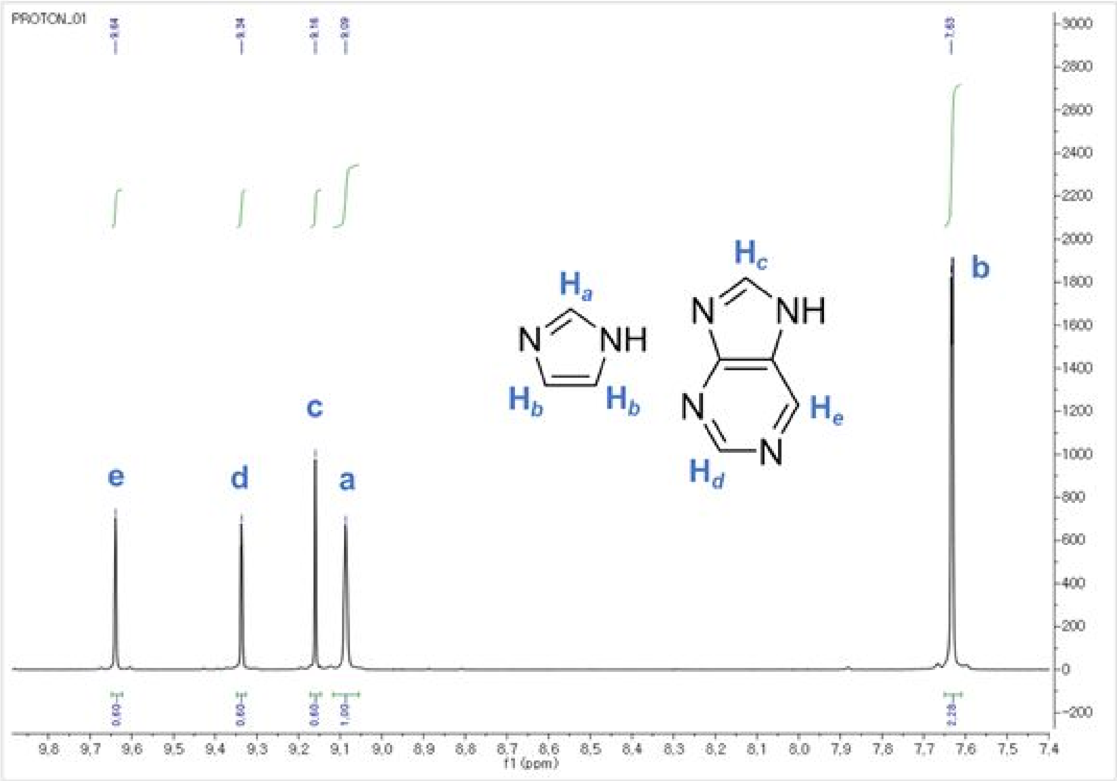

Figure S30. ^{1}H-NMR spectrum of the acid digested UZIF-33.

Thermal stability

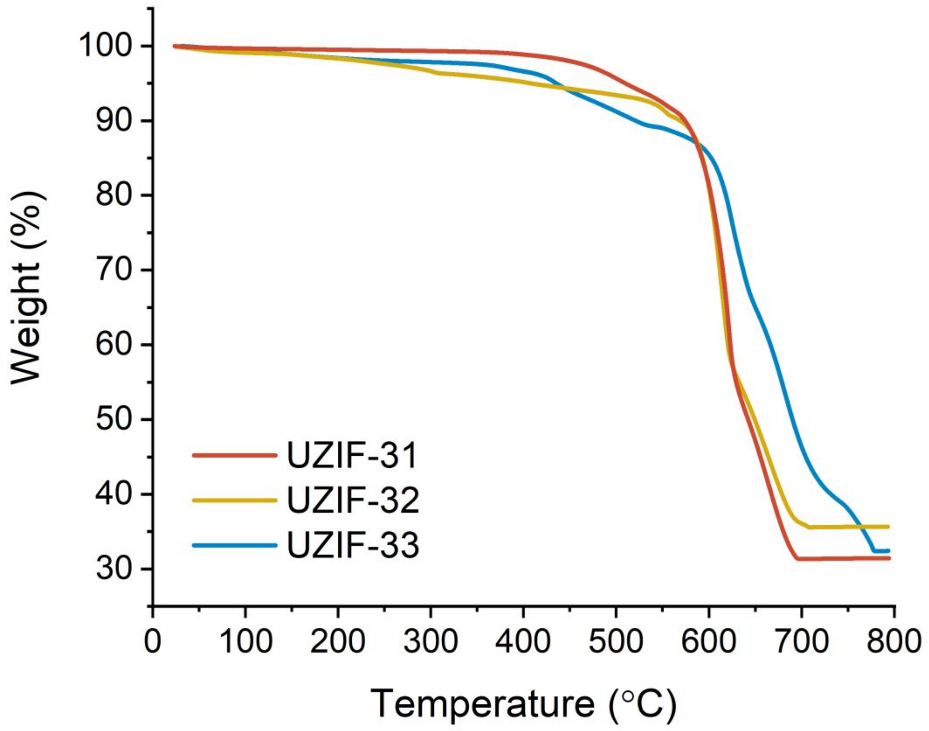

Figure S31. TGA curves of UZIF-31, UZIF-32, and UZIF-33 under air flow.

Topology and structure analysis

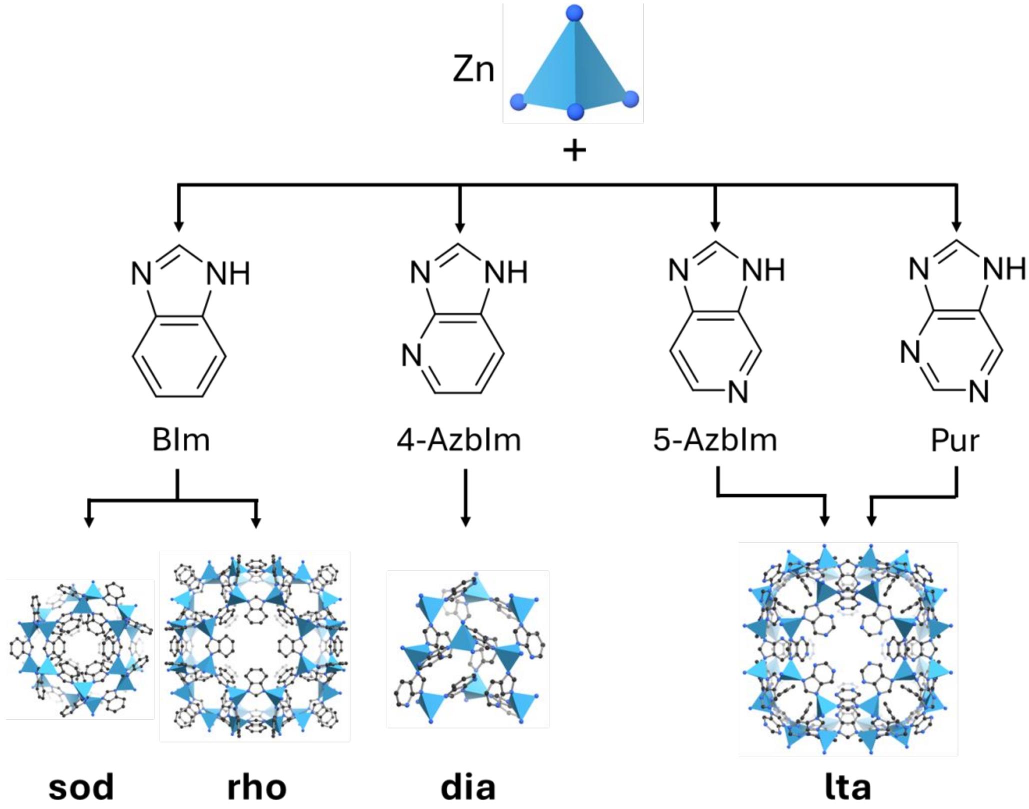

Figure S32. Resulting topologies in ZIF synthesis using single AzbIm derivatives. ZIF-7: **sod**, ZIF-11: **rho**, ZIF-23: **dia**, and ZIF-20, -22: **lta**.

Table S11. Coordination sequences and vertex symbols of *uft*1.

	cs1	cs2	cs3	cs4	cs5	cs6	cs7	cs8	cs9	cs10	Vertex symbol
T1(16.1)	4	10	20	32	47	69	95	122	153	189	$4.6_2.4.8_3.6.8_2$
T2(16.1)	4	9	18	32	48	68	95	124	153	187	4.6.4.6.4.8
T3(16.1)	4	9	18	31	47	69	96	123	152	188	$4.6.4.8.4.8_3$

Table S12. Coordination sequences and vertex symbols of *uft*2.

	cs1	cs2	cs3	cs4	cs5	cs6	cs7	cs8	cs9	cs10	Vertex symbol
T1(16.1)	4	9	18	33	52	72	96	129	166	200	$4.6.4.6_2.4.8$
T2(16.1)	4	11	21	31	48	74	103	130	155	194	$4.8_3.6.6_2.6_2.6_2$
T3(16.1)	4	10	20	34	52	71	94	128	167	202	$4.4.6.6_3.6_3.8_2$

Table S13. Tiling of *uft*1 and *uft*2.

Name	Tiling	Vertices	Edges	Faces	Tiles
*uft*1	$[4^6]+[4^4.6^6.8^2]+[4^6.6^2.8^2.12^2]$	3	9	9	3
*uft*2	$4[6^3]+4[4^2.6^2]+4[6^2.8^2]+[4^6]+2[4^2.6^4]$ $+[4^2.8^4]+[8^4.12^2]+[4^4.6^4.12^2]$	3	9	15	10

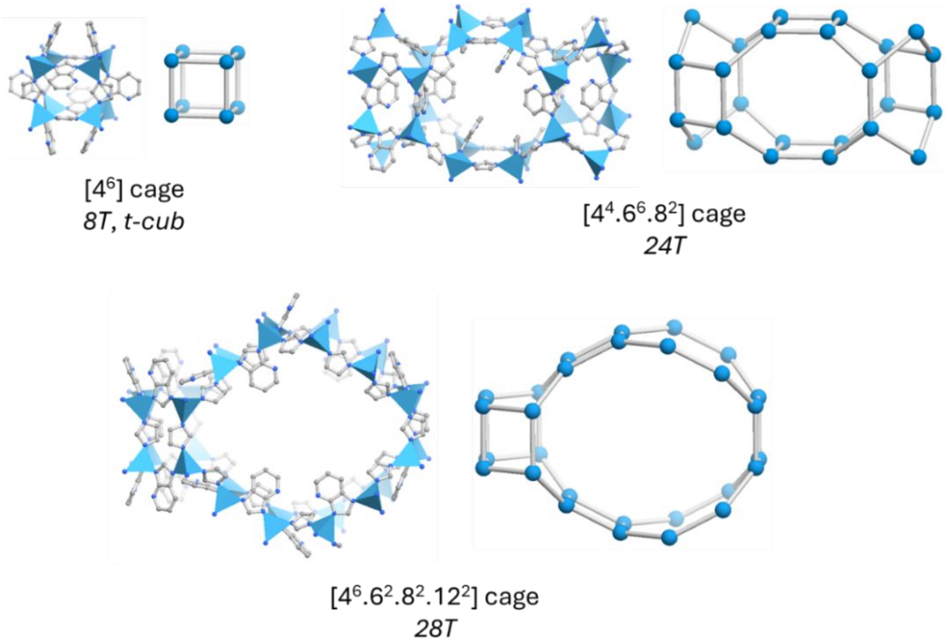

Figure S33. Three types of tile in UZIF-31 (*uft*1).

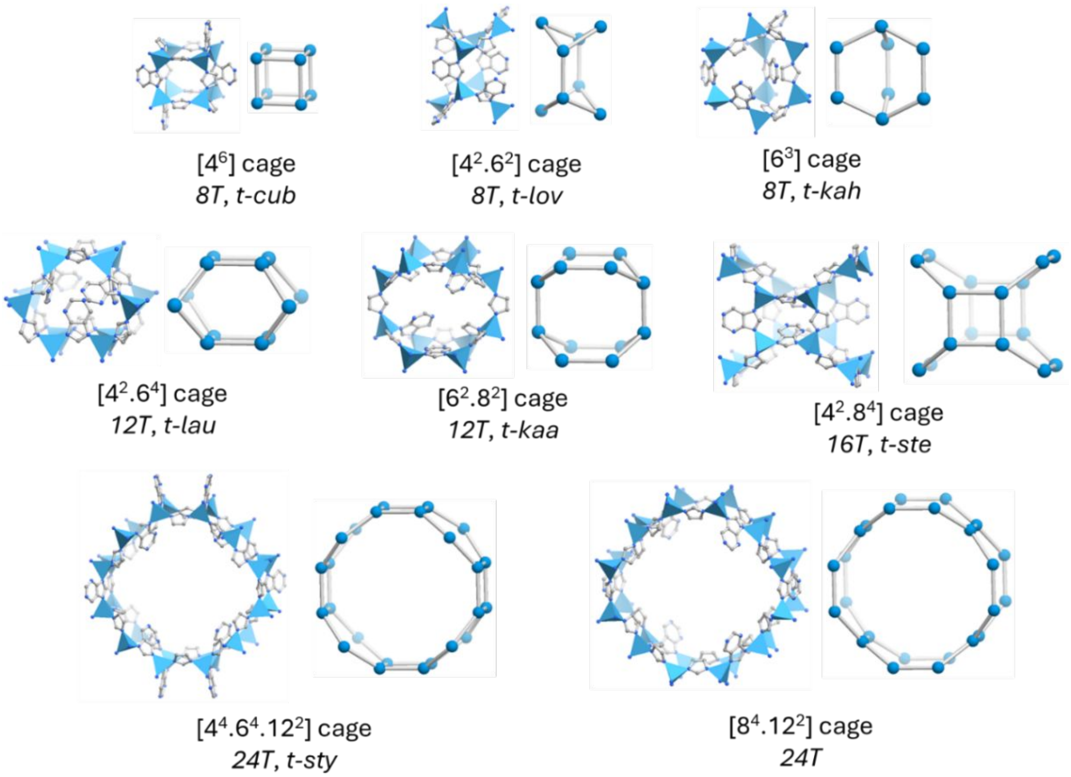

Figure S34. Eight types of tile in UZIF-32 and -33 (*uft*2).

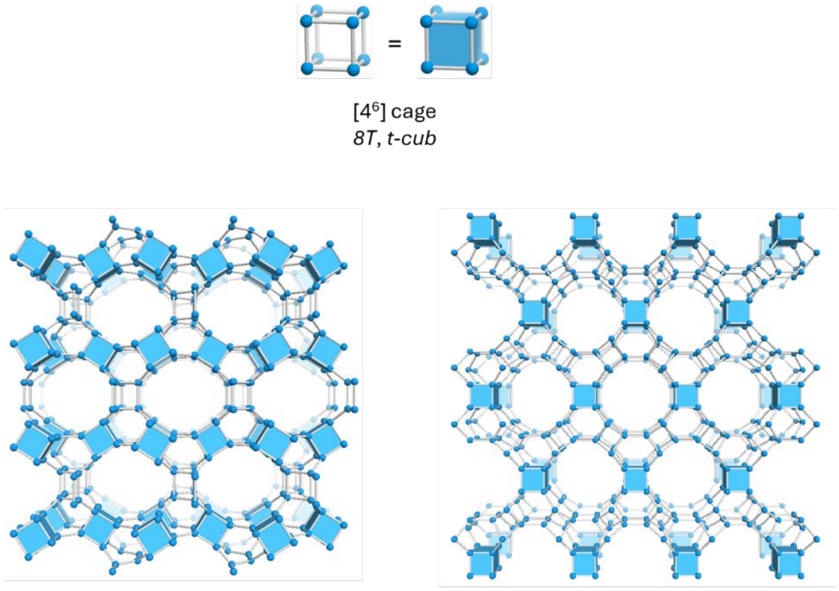

Figure S35. The arrangement of [4^6] cages (*t-cub*) in *uft*1 and *uft*2 topologies.

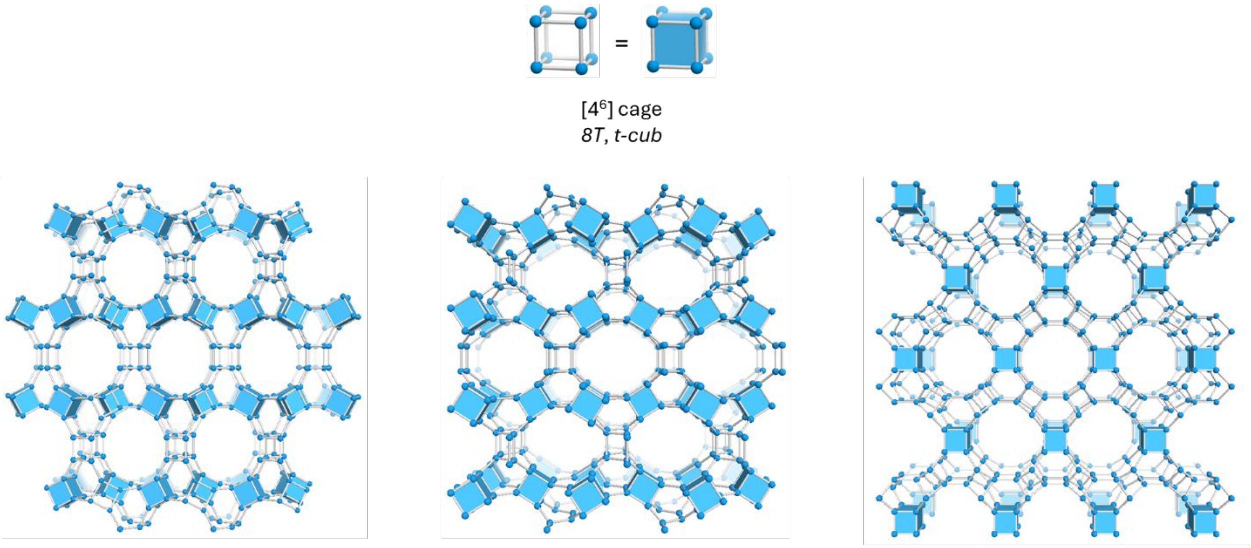

Figure S36. The arrangement of [4^6] cages (*t-cub*) in **zeb**, *uft*1, and *uft*2 topologies.

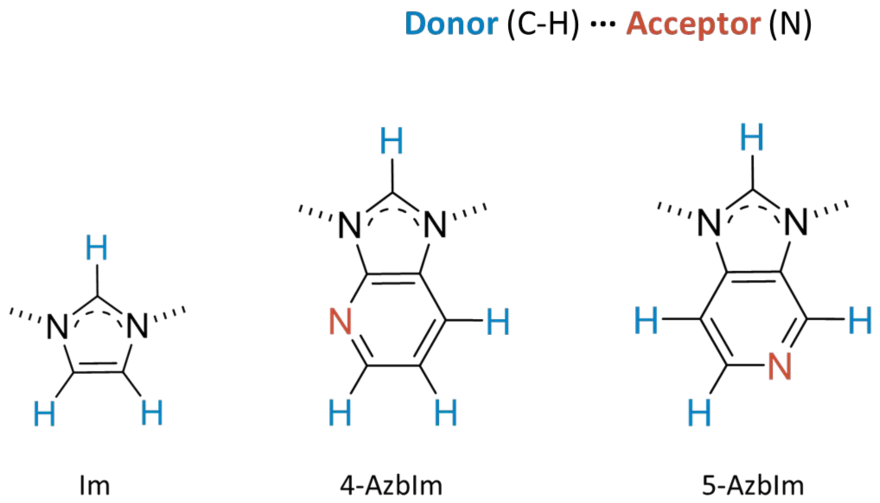

Figure S37. Hydrogen bond donor (C-H) and acceptor (N) atoms in imidazolates used in the synthesis of UZIF-31 (Im and 4-AzbIm), UZIF-32 (Im and 5-AzbIm), and UZIF-33 (Im and Pur).

View along *b*-axis

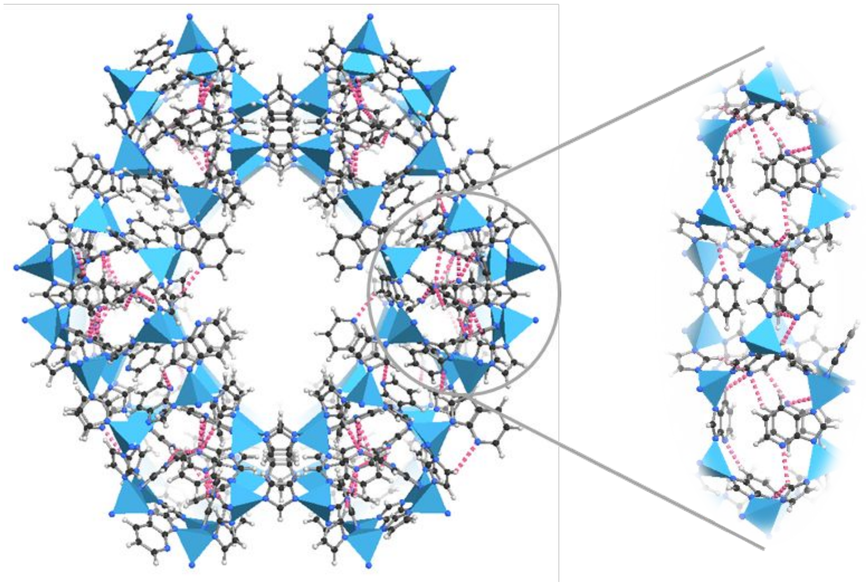

View along *a*-axis

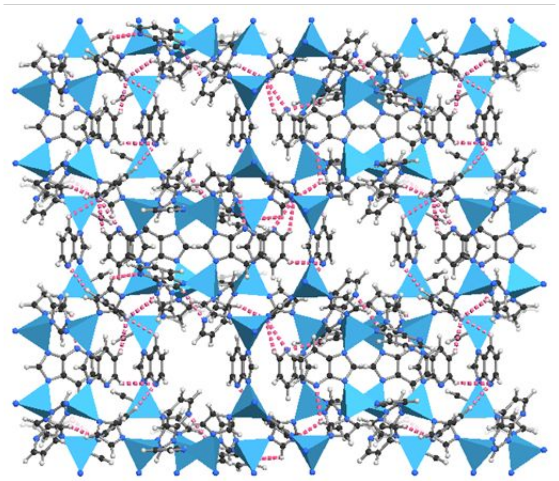

Figure S38. Hydrogen bonds (pink dotted line; within 3.0 Å) are represented along *b*-axis and *a*-axis of UZIF-31. Infinite chains of hydrogen bonds along *b*-axis are observed. Density of hydrogen bonds, *H/V* (nm^{-3}), is 2.91 nm^{-3}.

View along *c*-axis

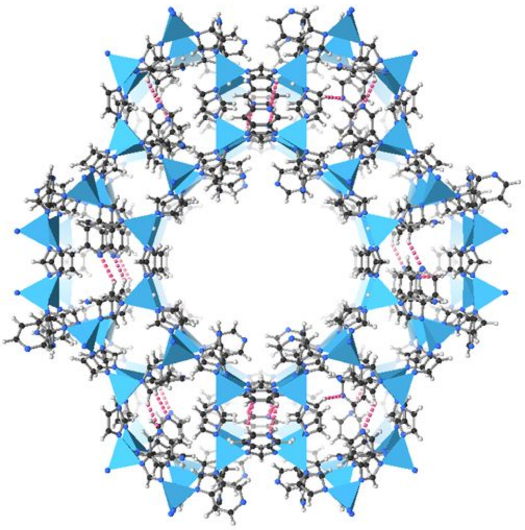

View along *b*-axis

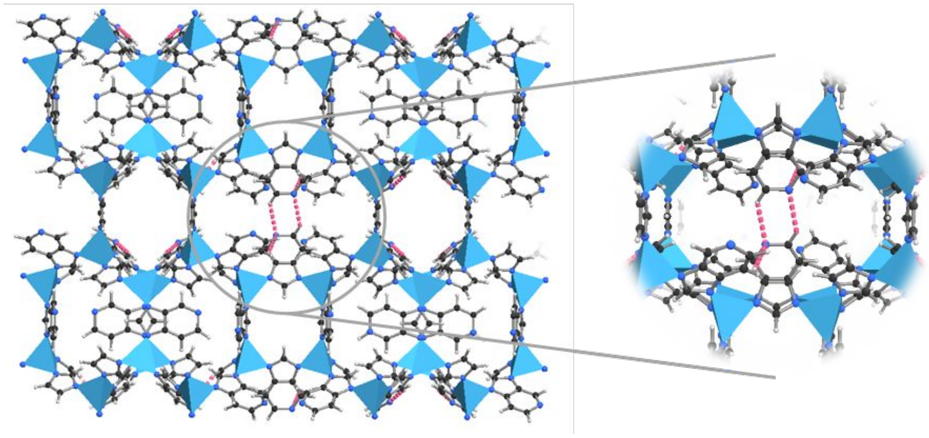

Figure S39. Hydrogen bonds (pink dotted line; within 3.0 Å) are represented along *c*-axis and *b*-axis of UZIF-32. Pair of hydrogen bonds between adjacent *t-cub* cages are observed. Density of hydrogen bonds, *H/V* (nm^{-3}), is 0.81 nm^{-3}.

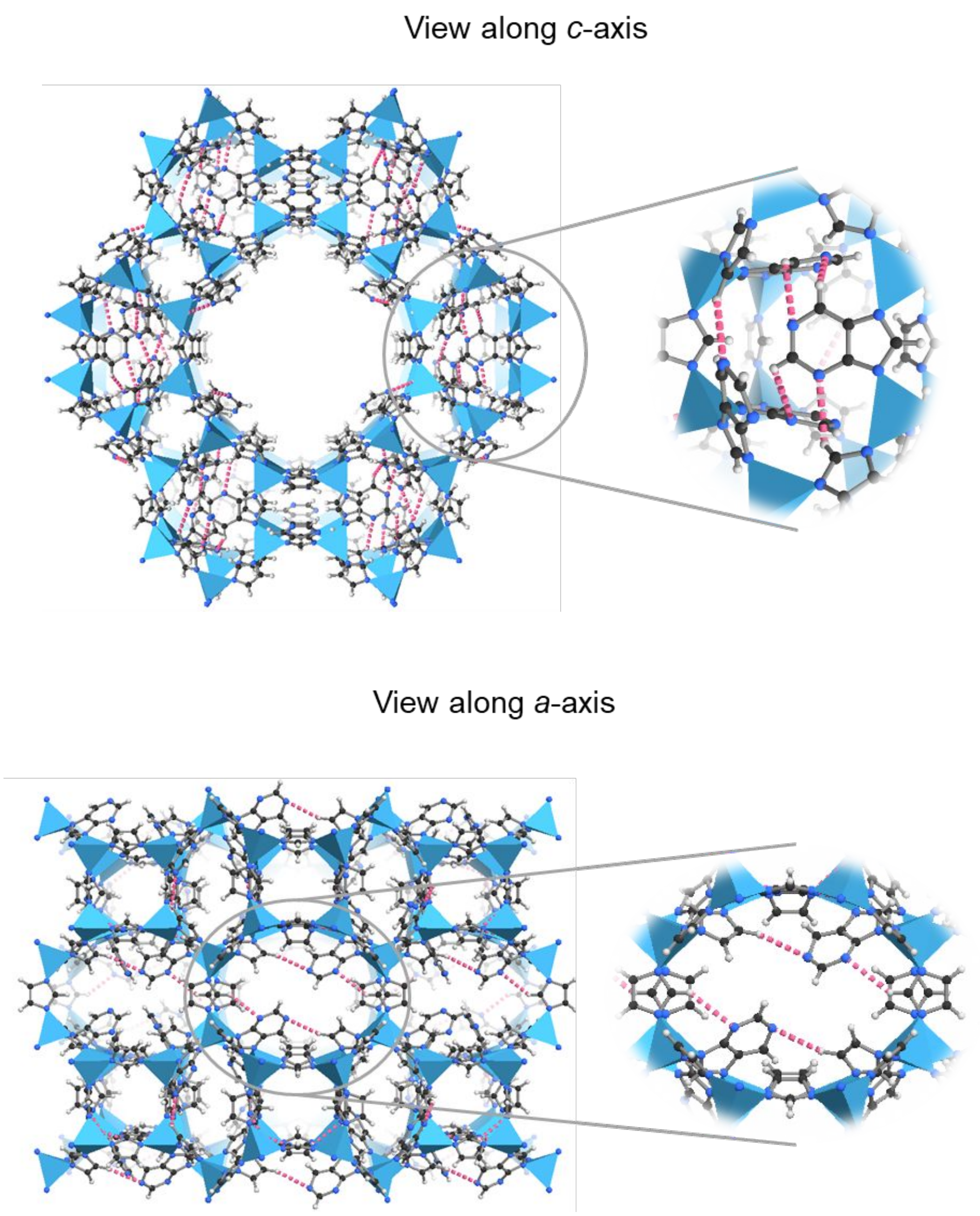

Figure S40. Hydrogen bonds (pink dotted line; within 3.0 Å) are represented along *c*-axis and *a*-axis of UZIF-33. Bundle of hydrogen bonds in a *t-lau* cage are observed when viewing to *c*-axis. Density of hydrogen bonds, *H/V* (nm^{-3}), is 1.82 nm^{-3}.

Table S14. Comparison of DFT calculated total energies for functionalized UZIF-31 and -32.

Name	Formula	Topology	Total energy (ev)	Total energy per atom (eV/atom)
UZIF-31_4-AzbIm	$Zn_{48}(Im)_{48}(4\text{-}AzbIm)_{48}$ $= Zn_{48}C_{432}N_{240}H_{336}$	*uft*1	-7299.697	-6.913
UZIF-31_5-AzbIm	$Zn_{48}(Im)_{48}(5\text{-}AzbIm)_{48}$ $= Zn_{48}C_{432}N_{240}H_{336}$	*uft*1	-7291.248	-6.905
UZIF-32_4-AzbIm	$Zn_{48}(Im)_{64}(4\text{-}AzbIm)_{32}$ $= Zn_{48}C_{384}N_{224}H_{320}$	*uft*2	-6665.570	-6.829
UZIF-32_5-AzbIm	$Zn_{48}(Im)_{64}(5\text{-}AzbIm)_{32}$ $= Zn_{48}C_{384}N_{224}H_{320}$	*uft*2	-6661.714	-6.826

Gas adsorption

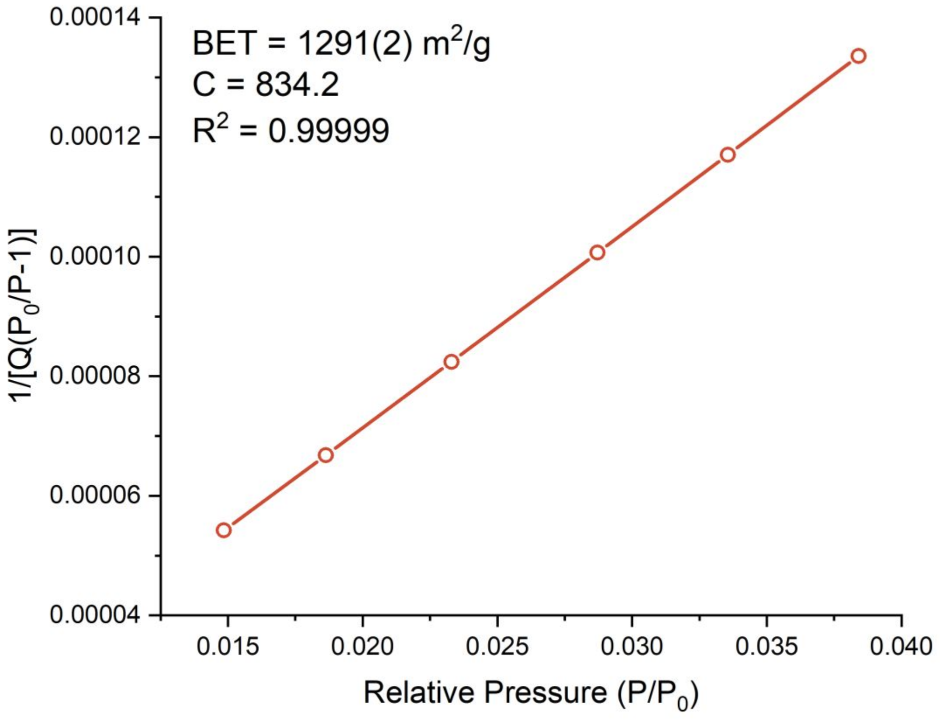

Figure S41. Plot of BET surface area of UZIF-31.

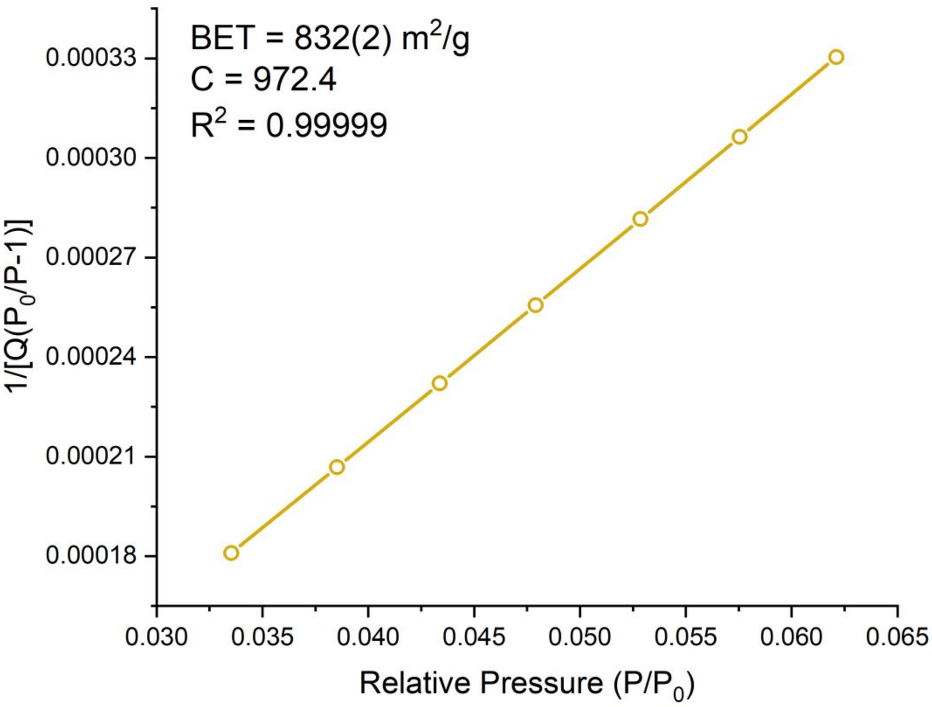

Figure S42. Plot of BET surface area of UZIF-32.

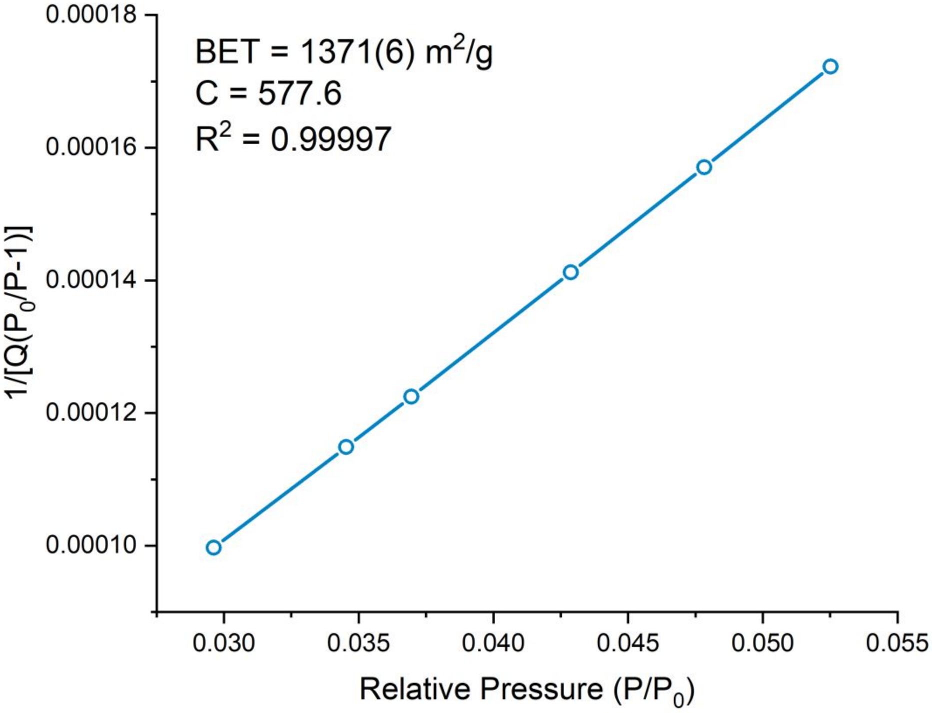

Figure S43. Plot of BET surface area of UZIF-33.

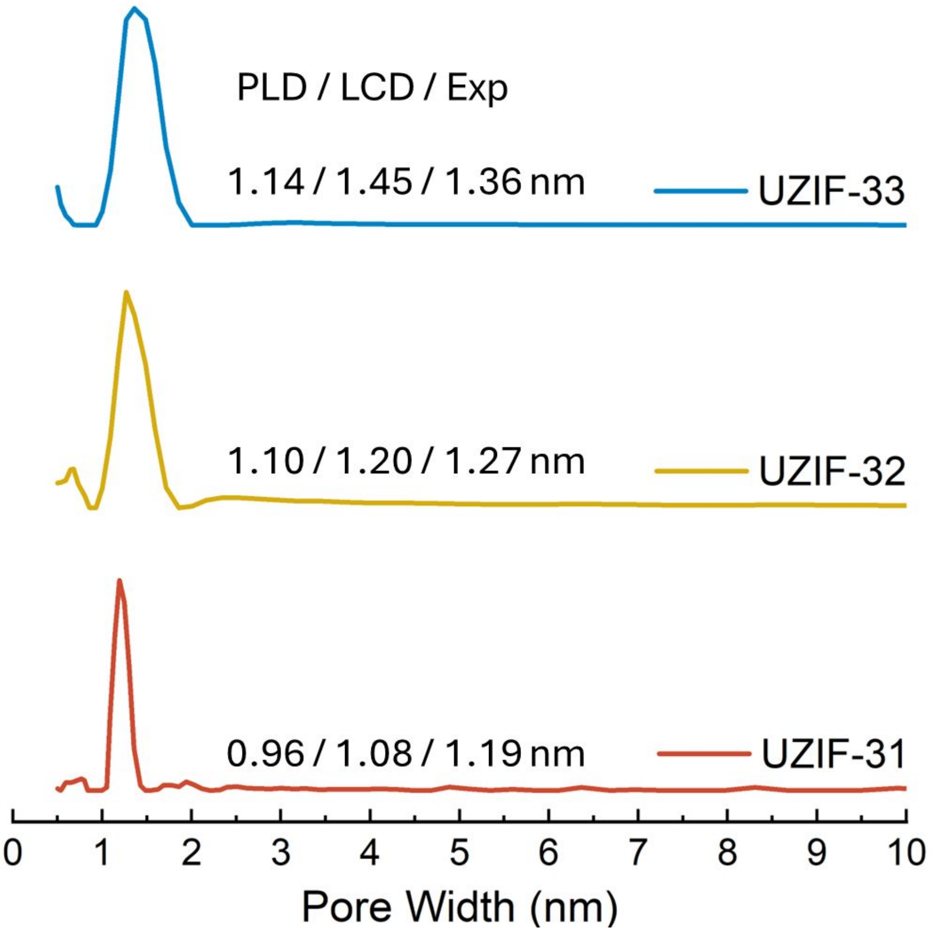

Figure S44. The pore size distribution of UZIF-31, UZIF-32, and UZIF-33 derived from N_2 adsorption at 77 K (PLD: pore-limiting diameter; LCD: largest cavity diameter; Exp: observed in pore size distribution).

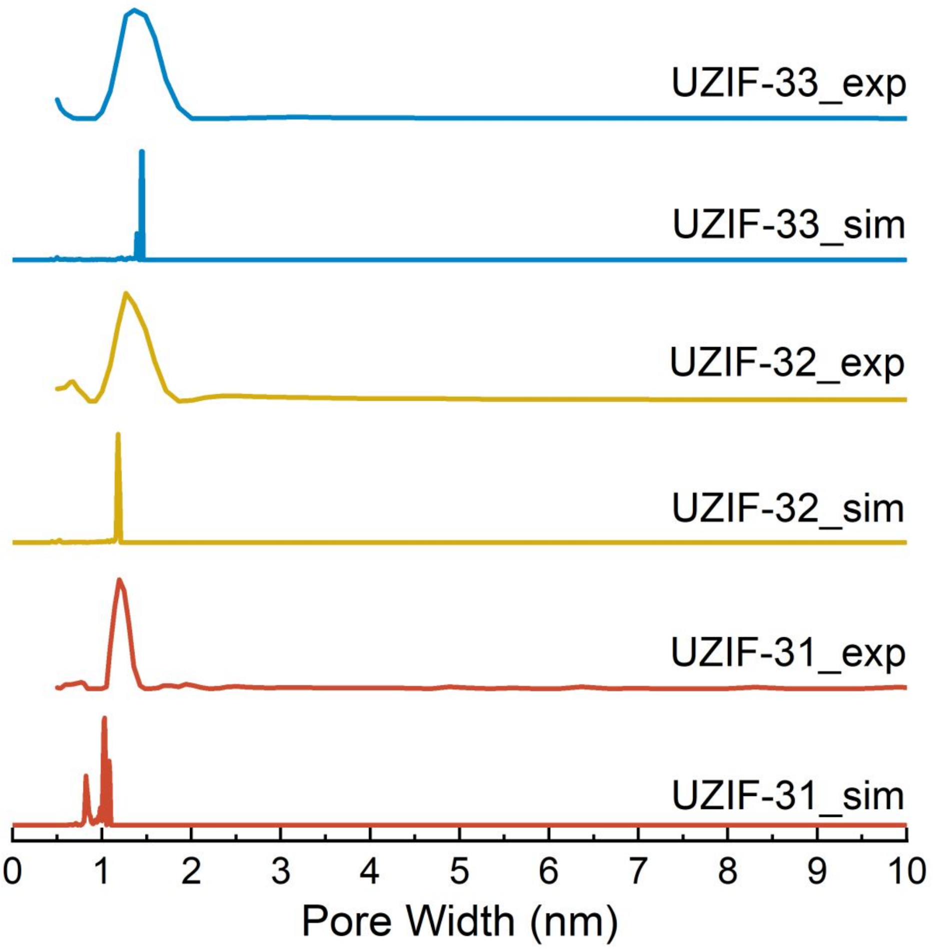

Figure S45. The comparison of experimental and simulated pore size distribution of UZIF-31, UZIF-32, and UZIF-33. Zeo++ was used to simulate the pore size distribution of ZIFs.

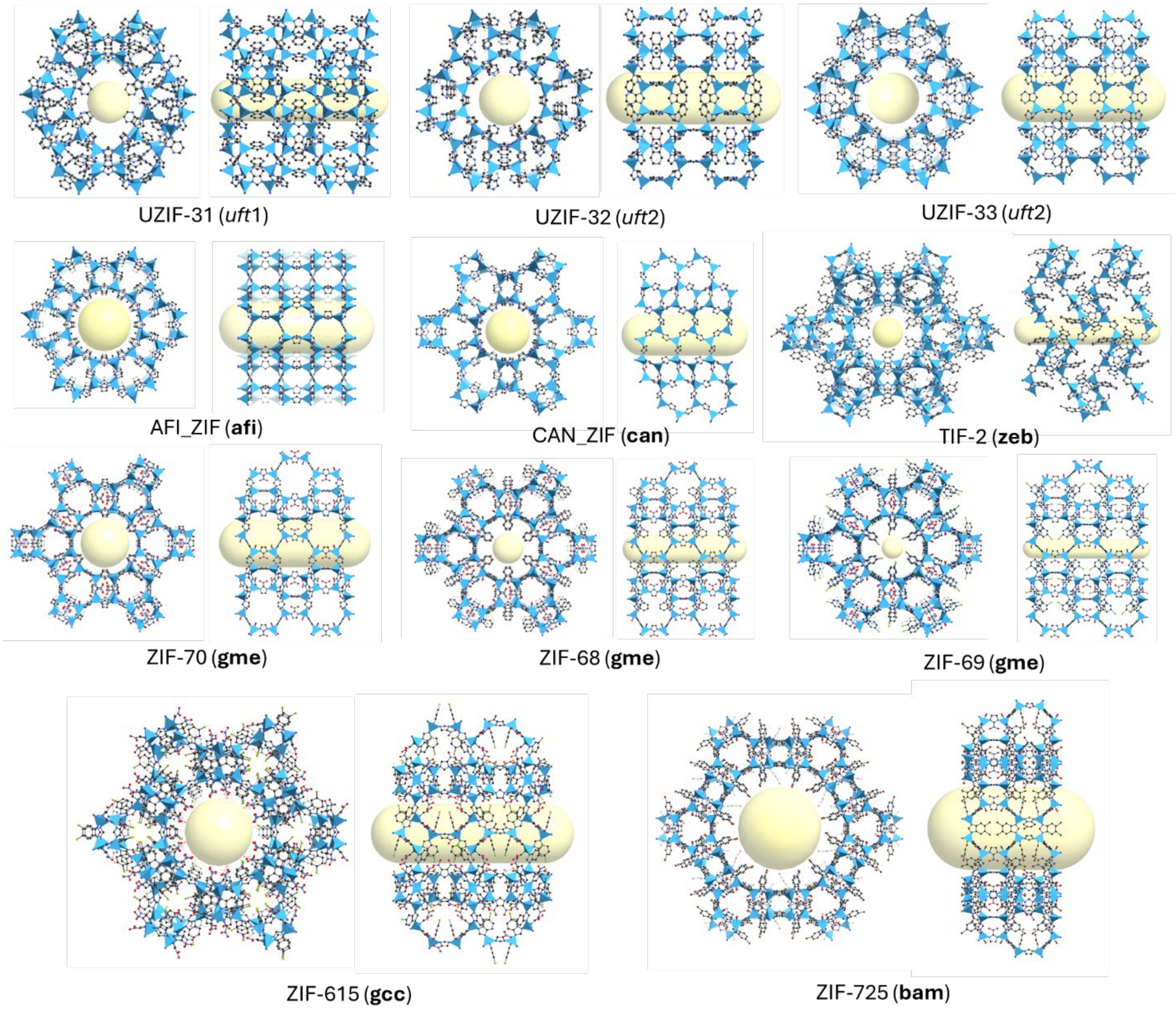

Figure S46. Schematic illustrations of reported channel-type pore ZIFs, including UZIF-31, UZIF-32, and UZIF-33. Pore (left) and intersection view (right).

Table S15. Comparison of reported channel-type pore ZIFs.

Name	Composition	Topology	T/V (nm^{-3})	R_{max}	PLD/LCD/Exp (nm)[b]	S_{BET} (m^2/g)	Ref.
UZIF-31	$Zn(Im)_{0.88}(4\text{-}AzbIm)_{1.12}$	*uft*1	2.33	12	0.96/1.08/1.19	1291	This work
UZIF-32	$Zn(Im)_{1.25}(5\text{-}AzbIm)_{0.75}$	*uft*2	2.42	12	1.10/1.20/1.27	832	This work
UZIF-33	$Zn(Im)_{1.25}(Pur)_{0.75}$	*uft*2	2.42	12	1.14/1.45/1.36	1376	This work
ZIF-70	$Zn(Im)_{1.13}(nIm)_{0.87}$	**gme**	2.10	12	1.31/1.59/N.A	1730	29
ZIF-68	Zn(nIm)(bIm)	**gme**	2.12	12	0.75/1.03/N.A	1090	29
ZIF-69	Zn(nIm)(cbIm)	**gme**	2.09	12	0.44/0.78/N.A	950	29
CAN_ZIF	$Zn(Im)_2$	**can**	2.56	12	1.12/1.18/1.27	1178	30
AFI_ZIF	$Zn(Im)_2$	**afi**	2.38	12	1.44/1.56/1.84	1386	30
TIF-2	$Zn(Im)_{1.1}(mbIm)_{0.9}$	**zeb**	2.31	12	0.85/1.09/N.A	618[a]	31
ZIF-615	$Zn(4\text{-}nIm)_{0.95}(cbIm)_{1.05}$	**gcc**	1.94	18	1.45/2.72/1.14	770	32
ZIF-725	$Zn(Im)_{0.25}(nIm)_{0.4}(bbIm)_{1.35}$	**bam**	1.54	24	2.25/3.90/3.11	720	32

[a] Langmuir surface area
[b] PLD: pore-limiting diameter; LCD: largest cavity diameter; Exp: observed in pore size distribution

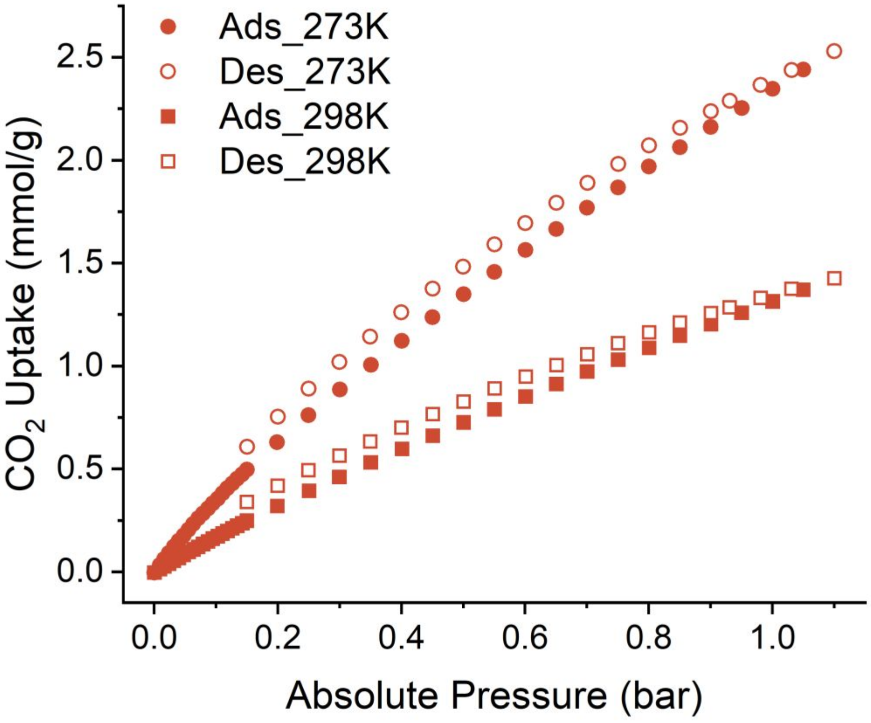

Figure S47. CO_2 adsorption isotherms at 273 and 298 K of UZIF-31.

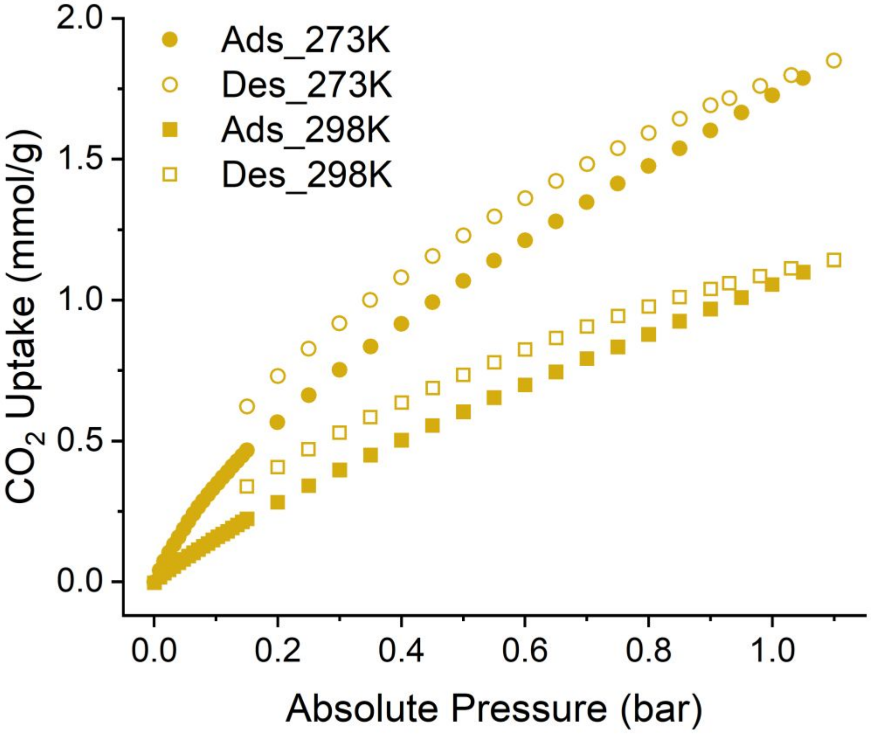

Figure S48. CO_2 adsorption isotherms at 273 and 298 K of UZIF-32.

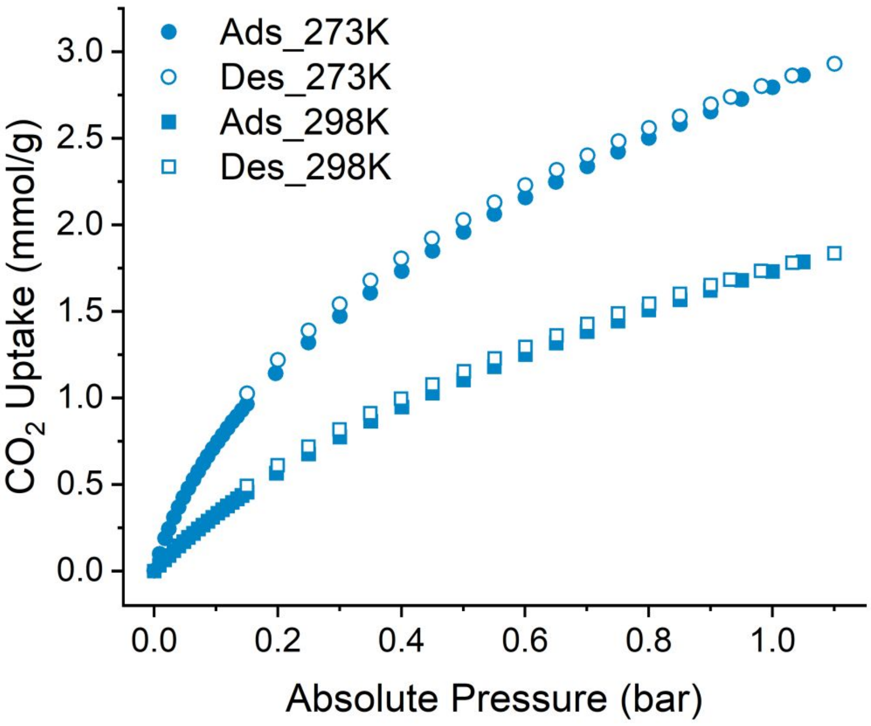

Figure S49. CO_2 adsorption isotherms at 273 and 298 K of UZIF-33.

Table S16. Comparison of azabenzimidazole-based ZIFs.

Name	Composition	Topology	*T/V* (nm^{-3})	R_{max}	S_{BET} (m^2/g)	Ref.
UZIF-31	$Zn(Im)_{0.88}(4\text{-AzbIm})_{1.12}$	*uft*1	2.33	12	1291	This work
UZIF-32	$Zn(Im)_{1.25}(5\text{-AzbIm})_{0.75}$	*uft*2	2.42	12	832	This work
UZIF-33	$Zn(Im)_{1.25}(Pur)_{0.75}$	*uft*2	2.42	12	1376	This work
ZIF-20	$Zn(Pur)_2$	**lta**	2.04	8	800[a]	33
ZIF-21	$Co(Pur)_2$	**lta**	2.04	8	N/A	33
ZIF-22	$Zn(5\text{-AzbIm})_2$	**lta**	2.02	8	N/A	33
ZIF-23	$Zn(4\text{-AzbIm})_2$	**dia**	3.32	6	N/A	33
-	Zn(nIm)(Pur)	**gme**	2.12	12	813	34
-	$Zn_{1.33}(O,OH)_{0.33}(nIm)_{1.67}(Pur)$	**rho**	1.96	8	578	34
UC-7	$Zn(Im)_{1.75}(Pur)_{0.25}$	**cag**	3.72	6	N/A	35
ZIF-7	$Zn(bIm)_2$	**sod**	2.49	6	N/A	36
ZIF-11	$Zn(bIm)_2$	**rho**	2.02	8	N/A	36

[a] Langmuir surface area

Heat of Adsorption (-Q_{st})

The heat of adsorption (-Q_{st}) of CO_2 for ZIFs were calculated based on Clausius-Clapeyron equation as

$$Q_{st} = -RT^2\left(\frac{\partial \ln P}{\partial T}\right)_q \quad (1)$$

from the single isotherm at 273 and 298 K. Each single isotherm was very precisely ($R^2 \geq 0.9999$) fitted by a dual-site Langmuir-Freundlich equation (DSLF) as

$$q = \frac{q_{sat,A} b_A p^{\alpha_A}}{1 + b_A p^{\alpha_A}} + \frac{q_{sat,B} b_B p^{\alpha_B}}{1 + b_B p^{\alpha_B}} \quad (2)$$

Table S17. Fitted DSLF parameters of CO_2 isotherms for UZIF-31.

	$q_{sat,A}$ (mmol g^{-1})	b_A (bar^{-1})	α_A	$q_{sat,B}$ (mmol g^{-1})	b_B (bar^{-1})	α_B	R^2
273K	0.24752	10.08736	1.12699	14.63814	0.16952	1.00164	1
298K	0.12845	9.35711	1.18819	8.46101	0.16509	1.05772	0.99999

Table S18. Fitted DSLF parameters of CO_2 isotherms for UZIF-32.

	$q_{sat,A}$ (mmol g^{-1})	b_A (bar^{-1})	α_A	$q_{sat,B}$ (mmol g^{-1})	b_B (bar^{-1})	α_B	R^2
273K	25.15078	0.06122	0.87182	0.30003	11.83333	1.14096	0.99999
298K	17.38172	0.01188	1.94989	2.11426	0.67361	0.93808	0.99999

Table S19. Fitted DSLF parameters of CO_2 isotherms for UZIF-33.

	$q_{sat,A}$ (mmol g^{-1})	b_A (bar^{-1})	α_A	$q_{sat,B}$ (mmol g^{-1})	b_B (bar^{-1})	α_B	R^2
273K	7.10447	0.3391	0.90661	1.19155	5.1091	0.93126	1
298K	1.77956	2.04166	0.9837	1.94017	0.38298	1.73477	1

IAST selectivity

The ideal adsorbed solution theory (IAST)[37] was used to calculate the selectivity for CO_2, C_2H_4, C_2H_6 over CH_4 from the single isotherms at 273 and 298 K of UZIF-33. Each single isotherm was very precisely ($R^2 \geq 0.9999$) fitted by a dual-site Langmuir-Freundlich equation (DSLF) as

$$q = \frac{q_{sat,A} b_A p^{\alpha_A}}{1+b_A p^{\alpha_A}} + \frac{q_{sat,B} b_B p^{\alpha_B}}{1+b_B p^{\alpha_B}} \tag{2}$$

In DSLF equation, q is the adsorbed amount per mass of adsorbent, $q_{sat,i}$ is the saturation loading for site i (i = A and B), b_i is the affinity constant for site i, p is the pressure of the bulk gas at equilibrium with the adsorbed phase and α_i is the deviation factor from the ideal homogeneous surface in simple Langmuir model. For CH_4, the isotherm was fitted with a single-site Langmuir-Freundlich equation.

From IAST, the adsorption selectivity of component 1 over component 2 in the binary mixture was defined as

$$S_{ads} = \frac{x_1/y_1}{x_2/y_2} = \frac{P_2^0}{P_1^0} \tag{3}$$

In equation 2, x_i (i = 1 and 2) is the adsorbed phase mole fraction, y_i is the gas phase mole fraction and P_i^0 is the equilibrium pressure of the single component gas.

Table S20. Fitted DSLF parameters of the isotherms for UZIF-33 at 273 K.

	$q_{sat,A}$ (mmol g^{-1})	b_A (bar^{-1})	α_A	$q_{sat,B}$ (mmol g^{-1})	b_B (bar^{-1})	α_B	R^2
CO_2	7.10447	0.3391	0.90661	1.19155	5.1091	0.93126	1
C_2H_4	18.96502	0.07866	0.76329	1.28138	10.21552	0.87622	0.99999
C_2H_6	1.26918	14.36249	0.93233	40.00425	0.04152	0.78881	0.99999
CH_4	1.97178	0.45681	0.99796	-	-	-	0.99997

Table S21. Fitted DSLF parameters for the isotherms for UZIF-33 at 298 K.

	$q_{sat,A}$ (mmol g^{-1})	b_A (bar^{-1})	α_A	$q_{sat,B}$ (mmol g^{-1})	b_B (bar^{-1})	α_B	R^2
CO_2	1.77956	2.04166	0.9837	1.94017	0.38298	1.73477	1
C_2H_4	0.84211	8.96319	1.00024	2.17749	0.77818	1.08983	1
C_2H_6	6.92387	0.15823	0.86625	1.09986	7.59203	0.96084	0.99999
CH_4	1.19127	0.3859	1.08762	-	-	-	0.99991

Table S22. Comparison of physicochemical property for UZIF-31, UZIF-32, and UZIF-33.

Name	Topology	*T/V* (nm^{-3})[a]	S_{BET} ($m^2\ g^{-1}$)[b]	*H/V* (nm^{-3})[c]	Chemical stability[d]	*N/V* (nm^{-3})[e]	CO_2 capacity ($cm^3\ g^{-1}$)[f]
UZIF-31	*uft*1	2.33	1291	2.91	pH 2 – pH 13	2.62	29.4
UZIF-32	*uft*2	2.42	832	0.81	pH 2 – pH 12	1.82	23.6
UZIF-33	*uft*2	2.42	1376	1.81	pH 7 – pH 12	3.63	38.8

[a] Number of tetrahedral nodes per volume
[b] BET surface area
[c] Number of hydrogen bonds per volume
[d] Conditions with maintaining PXRD pattern after 1 month
[e] Number of exposed nitrogen atoms per volume
[f] Measured at 298K and 1bar

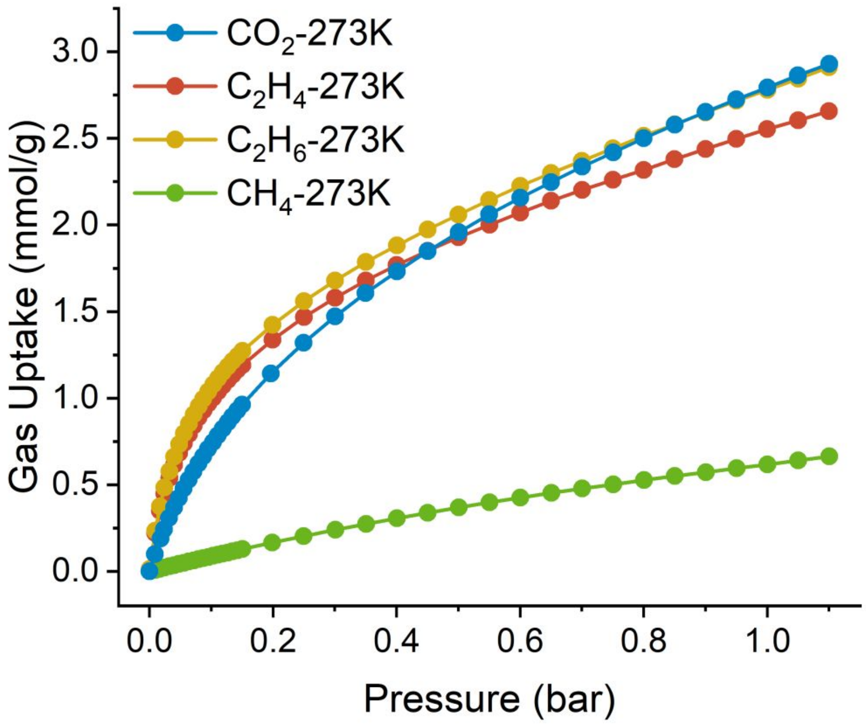

Figure S50. Experimental adsorption isotherms of CO_2, C_2H_4, C_2H_6, and CH_4 for UZIF-33 measured at 273 K.

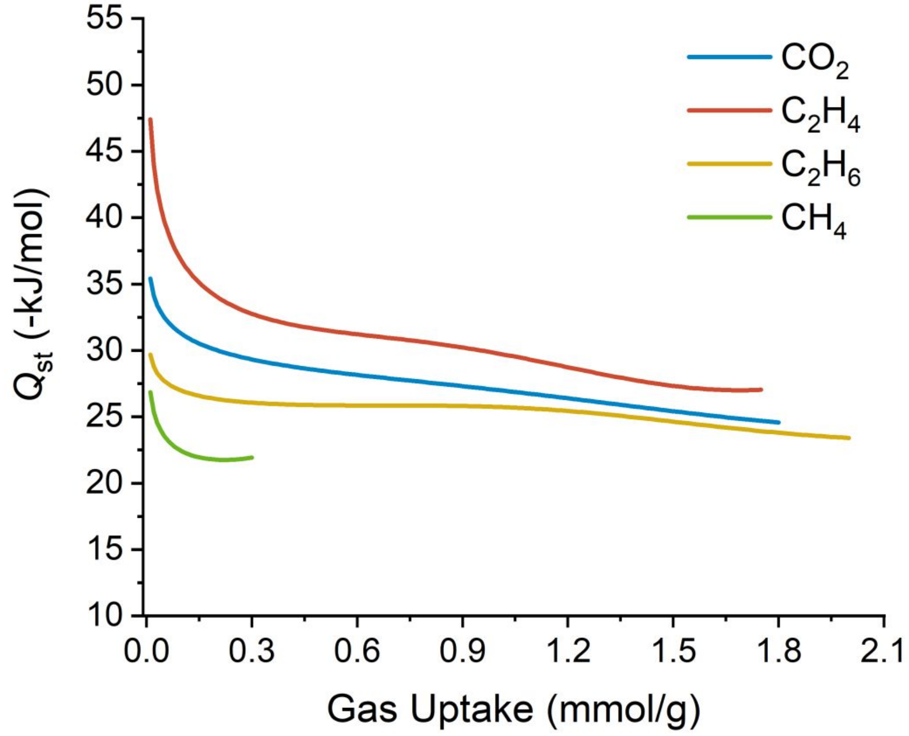

Figure S51. The Q_{st} value of CO_2, C_2H_4, C_2H_6, and CH_4 for UZIF-33.

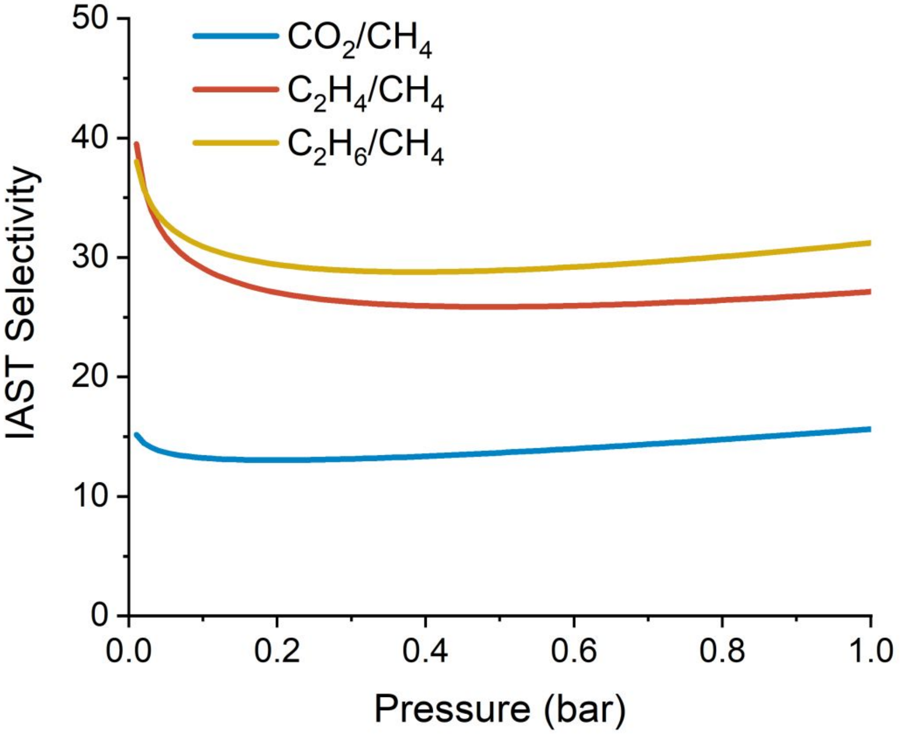

Figure S52. Predicted IAST selectivities of CO_2/CH_4, C_2H_4/CH_4, and C_2H_6/CH_4 for UZIF-33 with equimolar mixtures (50/50) at 273 K.

Table S23. Comparison of CO_2/CH_4 selectivity in ZIFs.

Name	Topology	S_{BET} (m^2/g)[a]	CO_2 capacity (cm^3/g)[b]	$s(CO_2/CH_4)$[c]	Reference
UZIF-33	*uft2*	1371	38.8	11.9	This work
ZIF-70	**gme**	1730	30.0	5.2[d]	29, 38
ZIF-78	**gme**	620	51.5	10.6[d]	29, 38
ZIF-95	**poz**	1050	18.0	4.3[d]	39
ZIF-100	**moz**	595	20.0	5.9[d]	39
COK-17	**sod**	500	56.0	4.5	40
ZIF-8	**sod**	1475	19.1	2.8	41
BPL carbon	**-**	985	44.8	4.0	41

[a] BET surface area
[b] Measured at 298K and 1bar
[c] IAST selectivity at 298K and 1bar
[d] Henry's law selectivity

Dynamic breakthrough experiments.

Single-component and binary (CO_2/CH_4) mixture breakthrough experiments were conducted using a home-built breakthrough device (Figure S53). Approximately 1,354 mg of UZIF-33 pelletized at 86.5 MPa was loaded into an ¼ inch stainless-steel tube with an inner diameter of 4 mm and length of 120 mm ($V_{column} = 1.85\ cm^3$). We utilized 4 mass flow controllers (MFCs) to ensure a steady gas flow. Two of the MFCs provided a flow of dry neon and helium (purity 99.999%) as a carrier gas and dead volume indicator during all experiment stages. This was done to establish a consistent baseline flow to the detector. The other two MFCs used static inline mixers to create gas mixtures for all feed compositions. A Hiden quantitative gas analyzer (QGA) was used to monitor the gas outlet composition. The 44 and 16 m/z signals were used for carbon dioxide and methane, respectively. Since carbon dioxide also contains a fragment of 16 m/z signal, a calibration run was conducted first to account for this signal contribution from methane. The manuscript explains how to calculate the uptakes of individual components from the breakthrough curves.

Each working gas data acquired from the experiment was normalized by dividing instantaneous intensity ($y_{ads(t)}$) by the intensity at the equilibrium point ($y_{eq,ads}$), and normalized data was plotted versus the time per gram plot. From the normalized intensity at the equilibrium point, the normalized intensity at the specific time was subtracted $\left(1-\frac{y_{ads(t)}}{y_{eq,ads}}\right)$. The integrity area under the breakthrough curve (t=0 to t) was calculated.

The measured dead volume using Helium was subtracted from the calculated value, expressed as (y_{inert}), which was calculated using the same formula as the reaction gas uptake. The x-axis was made time-domain independent by multiplying the mass of the adsorbent. To obtain the final value in units of mmol g^{-1}, the calculated value needs to be multiplied by the flow rate ($Q_{in,ads}$) in mL min^{-1} and then divided by 22.4. (1 mmol of gas at STP ~ 22.4 mL, ideal gas law)

Following is the equation for uptake calculation. n_{ads} represents the uptake of the working gas.

$$n_{ads} = \frac{Q_{in,ads}}{22.4}\left(\underbrace{\int_0^t\left(1-\frac{y_{ads(t)}}{y_{eq,ads}}\right)\cdot dt}_{\text{Working gas sorption}} - \underbrace{m\int_0^t\left(1-\frac{y_{inert}}{y_{inert.eq}}\right)\cdot dt}_{\text{Dead Volume Correction}}\right)$$

UZIF-33 was activated at 120℃ under a dynamic vacuum. After being cooled to the experiment temperature of 25°C (lasting at least 30 minutes), a dynamic flow of the adsorbate mixture was introduced. Before each analysis, the adsorbent underwent a 2-hour regeneration process under a dynamic high vacuum, which was consistently done between every analysis. The flow rate of the binary mixture working gas was CO_2:CH_4=1:1 with a total flow rate of 5 mL min^{-1}, along with an additional 3 mL min^{-1} of Neon as a carrier gas and 0.2 mL min^{-1} of Helium as a dead volume indicator. For single gas adsorption, the working gas flow rate was 5 mL min^{-1} with 3 mL min^{-1} of Neon as a carrier gas, and 0.2 mL min^{-1} of helium was used as dead volume indicator.

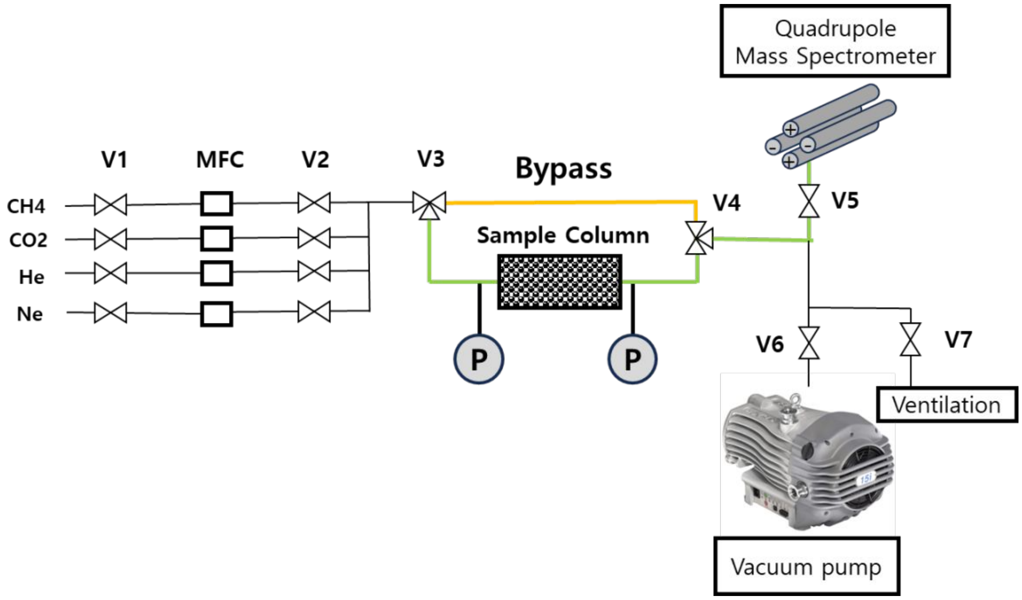

Figure S53. Setup of dynamic column gas adsorption breakthrough experiment.

References

1. Colón, Y.J., Gómez-Gualdrón, D.A., and Snurr, R.Q. (2017). Topologically Guided, Automated Construction of Metal–Organic Frameworks and Their Evaluation for Energy-Related Applications. Cryst. Growth Des. *17*, 5801–5810.

2. Thompson, A.P., Aktulga, H.M., Berger, R., Bolintineanu, D.S., Brown, W.M., Crozier, P.S., In 'T Veld, P.J., Kohlmeyer, A., Moore, S.G., Nguyen, T.D., et al. (2022). LAMMPS - a flexible simulation tool for particle-based materials modeling at the atomic, meso, and continuum scales. Comput. Phys. Commun. *271*, 108171.

3. Dürholt, J.P., Fraux, G., Coudert, F.-X., and Schmid, R. (2019). Ab Initio Derived Force Fields for Zeolitic Imidazolate Frameworks: MOF-FF for ZIFs. J. Chem. Theory Comput. *15*, 2420–2432.

4. Lewis, D.W., Ruiz-Salvador, A.R., Gómez, A., Rodriguez-Albelo, L.M., Coudert, F.-X., Slater, B., Cheetham, A.K., and Mellot-Draznieks, C. (2009). Zeolitic imidazole frameworks: structural and energetics trends compared with their zeolite analogues. CrystEngComm *11*, 2272.

5. Kresse, G.; Furthmüller, J. (1996). Efficiency of Ab-initio Total Energy Calculations for Metals and Semiconductors Using A Plane-wave Basis Set. Comput. Mater. Sci. *6*, 15–50.

6. Kresse, G.; Furthmüller, J. (1996). Efficient Iterative Schemes for Ab initio Total-energy Calculations using A Plane-wave Basis Set. Phys. Rev. B *54*, 11169–11186.

7. Kresse, G.; Joubert, D. (1999). From Ultrasoft Pseudopotentials to The Projector Augmented-wave Method, Phys. Rev. B *59*, 1758–1775.

8. Perdew, J.P.; Burke, K.; Ernzerhof, M. (1996). Generalized Gradient Approximation Made Simple. Phys. Rev. Lett, *77*, 3865–3868.
9. Perdew, J.P.; Burke, K.; Ernzerhof, M. (1997). Errata: Generalized Gradient Approximation Made Simple [Phys. Rev. Lett. 77, 1996, 3865]. Phys. Rev. Lett. *78*, 1396–1396.
10. Grimme, S.; Antony, J.; Ehrlich, S.; Krieg, H. (2010). A Consistent and Accurate Ab Initio Parametrization of Density Functional Dispersion Correction (DFT-D) for The 94 Elements H-Pu. J. Chem. Phys. *132*, 154104.
11. Johnson, E.R.; Becke, A.D. (2005). A Post-Hartree–Fock Model of Intermolecular Interactions. J. Chem. Phys. *123*, 024101.
12. Becke, A.D.; Johnson, E.R. (2005). A Density-functional Model of The Dispersion Interaction. J. Chem. Phys. *123*, 154101.
13. Johnson, E.R.; Becke, A.D. (2006). A Post-Hartree-Fock Model of Intermolecular Interactions: Inclusion of Higher-order Corrections. J. Chem. Phys. *124*, 174104.
14. Grimme, S.; Ehrlich, S.; Goerigk, L. (2011). Effect of The Damping Function in Dispersion Corrected Density Functional Theory. J. Comput. Chem. *32*, 1456–1465.
15. Grimme, S., Bannwarth, C., and Shushkov, P. (2017). A robust and accurate tight-binding quantum chemical method for structures, vibrational frequencies, and noncovalent interactions of large molecular systems parametrized for all spd-block elements (Z = 1–86). J. Chem. Theory Comput. *13*, 1989−2009.
16. Bannwarth, C., Ehlert, S., and Grimme, S. (2019). GFN2-xTB–an accurate and broadly parametrized self-consistent tight-binding quantum chemical method with multipole electrostatics and density-dependent dispersion contributions. J. Chem. Theory Comput. *15*, 1652−1671.
17. Hourahine, B., Aradi, B., Blum, V., Bonafé, F., Buccheri, A., Camacho, C., Cevallos, C., Deshaye, M.Y., Dumitrică, T., Dominguez, A., et al. (2020). DFTB+, a software package for efficient approximate density functional theory based atomistic simulations. J. Chem. Phys. *152*, 124101.
18. Hjorth Larsen, A., Jørgen Mortensen, J., Blomqvist, J., Castelli, I.E., Christensen, R., Dułak, M., Friis, J., Groves, M.N., Hammer, B., Hargus, C., et al. (2017). The atomic simulation environment—a Python library for working with atoms. J. Phys.: Condens. Matter *29*, 273002.
19. Blöchl, P.E. (1994). Projector Augmented-Wave Method. Phys. Rev. B *50*, 17953−17979.
20. Kresse, G., Hafner, J. (1993). Ab Initio Molecular Dynamics for Liquid Metals. Phys. Rev. B *47*, 558−561.
21. Kresse, G., Hafner, J. (1994). Ab Initio Molecular-Dynamics Simulation of the Liquid-Metal–Amorphous-Semiconductor Transition in Germanium. Phys. Rev. B *49*, 14251−14269.
22. Willems, T.F., Rycroft, C.H, Kazi, M., Meza, J.C., and Haranczyk, M. (2012). Algorithms and tools for high-throughput geometry-based analysis of crystalline porous materials. Microporous Mesoporous Mater. *149*, 134−141.
23. Rouquerol, J., Llewellyn, P., and Rouquerol, F. (2007) In *Studies in Surface Science and Catalysis*; Llewellyn, P. L., Rodriquez-Reinoso, F., Rouquerol, J., Seaton, N., Eds.; Elsevier: Amsterdam, Vol. 160, p 49.

24. Sheldrick, G.M. (2008). A short history of SHELX. Acta Cryst. A *64*, 112−122.

25. Dolomanov, O.V., Bourhis, L.J., Gildea, R.J., and Puschmann, H. (2009). OLEX2: a complete structure solution, refinement and analysis program. J. Appl. Cryst. *42*, 339−341.

26. Rees, B., Jenner, L., and Yusupov, M. (2005). Bulk-solvent correction in large macromolecular structures. Acta. Cryst. D *61*, 1299−1301.

27. Speck, A.L. (2009). Structure validation in chemical crystallography. Acta. Cryst. D *65*, 148−155.

28. Lee, S., Nam, D., Yang, D.C., and Choe, W. (2023). Unveiling Hidden Zeolitic Imidazolate Frameworks Guided by Intuition-Based Geometrical Factors. Small *19*, 2300036.

29. Banerjee, R., Phan, A., Wang, B., Knobler, C., Furukawa, H., O'Keeffe, M., and Yaghi, O.M. (2008). High-Throughput Synthesis of Zeolitic Imidazolate Frameworks and Application to CO_2 Capture. Science *319*, 939–943.

30. Shi, Q., Xu, W.-J., Huang, R.-K., Zhang, W.-X., Li, Y., Wang, P., Shi, F.-N., Li, L., Li, J., and Dong, J. (2016). Zeolite CAN and AFI-Type Zeolitic Imidazolate Frameworks with Large 12-Membered Ring Pore Openings Synthesized Using Bulky Amides as Structure-Directing Agents. J. Am. Chem. Soc. *138*, 16232–16235.

31. Wu, T., Bu, X., Zhang, J., and Feng, P. (2008). New Zeolitic Imidazolate Frameworks: From Unprecedented Assembly of Cubic Clusters to Ordered Cooperative Organization of Complementary Ligands. Chem. Mater. *20*, 7377–7382.

32. Yang, J., Zhang, Y.-B., Liu, Q., Trickett, C.A., Gutiérrez-Puebla, E., Monge, M.Á., Cong, H., Aldossary, A., Deng, H., and Yaghi, O.M. (2017). Principles of Designing Extra-Large Pore Openings and Cages in Zeolitic Imidazolate Frameworks. J. Am. Chem. Soc. *139*, 6448–6455.

33. Hayashi, H., Côté, A.P., Furukawa, H., O'Keeffe, M., and Yaghi, O.M. (2007). Zeolite A imidazolate frameworks. Nat. Mater. *6*, 501–506.

34. Kahr, J., Mowat, J.P.S., Slawin, A.M.Z., Morris, R.E., Fairen-Jimenez, D., and Wright, P.A. (2012). Synthetic control of framework zinc purinate crystallisation and properties of a large pore, decorated, mixed-linker RHO-type ZIF. Chem. Commun. *48*, 6690.

35. Bumstead, A.M., Castillo-Blas, C., Pakamorė, I., Thorne, M.F., Sapnik, A.F., Chester, A.M., Robertson, G., Irving, D.J.M., Chater, P.A., Keen, D.A., et al. (2023). Formation of a meltable purinate metal–organic framework and its glass analogue. Chem. Commun. *59*, 732–735.

36. Park, K.S., Ni, Z., Côté, A.P., Choi, J.Y., Huang, R., Uribe-Romo, F.J., Chae, H.K., O'Keeffe, M., and Yaghi, O.M. (2006). Exceptional chemical and thermal stability of zeolitic imidazolate frameworks. Proc. Natl. Acad. Sci. U.S.A. 103, 10186–10191.

37. Myers, A.L., and Prausnitz, J.M. (1965). Thermodynamics of mixed-gas adsorption. AIChE J. 11, 121–127.

38. Banerjee, R., Furukawa, H., Britt, D., Knobler, C., O'Keeffe, M., and Yaghi, O.M. (2009). Control of Pore Size and Functionality in Isoreticular Zeolitic Imidazolate Frameworks and their Carbon Dioxide Selective Capture Properties. J. Am. Chem. Soc. *131*, 3875–3877.

39. Wang, B., Côté, A.P., Furukawa, H., O'Keeffe, M., and Yaghi, O.M. (2008). Colossal cages in zeolitic imidazolate frameworks as selective carbon dioxide reservoirs. Nature *453*, 207–211.
40. Wee, L.H., Vandenbrande, S., Rogge, S.M.J., Wieme, J., Asselman, K., Jardim, E.O., Silvestre-Albero, J., Navarro, J.A.R., Van Speybroeck, V., Martens, J.A., et al. (2021). Chlorination of a Zeolitic-Imidazolate Framework Tunes Packing and van der Waals Interaction of Carbon Dioxide for Optimized Adsorptive Separation. J. Am. Chem. Soc. *143*, 4962–4968.
41. McEwen, J., Hayman, J.-D., and Ozgur Yazaydin, A. (2013). A comparative study of CO_2, CH_4 and N_2 adsorption in ZIF-8, Zeolite-13X and BPL activated carbon. Chem. Phys. *412*, 72–76.
